# Logic‐Based Strategy for Spatiotemporal Release of Dual Extracellular Vesicles in Osteoarthritis Treatment

**DOI:** 10.1002/advs.202403227

**Published:** 2024-05-05

**Authors:** Shiyu Li, Weihan Zheng, Wenfeng Deng, Ziyue Li, Jiaxin Yang, Huihui Zhang, Zhenning Dai, Weiwei Su, Zi Yan, Wanting Xue, Xinyi Yun, Siqi Mi, Jianlin Shen, Xiang Luo, Ling Wang, Yaobin Wu, Wenhua Huang

**Affiliations:** ^1^ Guangdong Medical Innovation Platform for Translation of 3D Printing Application The Third Affiliated Hospital of Southern Medical University Southern Medical University Guangzhou 510630 China; ^2^ Guangdong Engineering Research Center for Translation of Medical 3D Printing Application Guangdong Provincial Key Laboratory of Digital Medicine and Biomechanics National Key Discipline of Human Anatomy School of Basic Medical Sciences Southern Medical University Guangzhou 510515 China; ^3^ Department of Obstetrics and Gynecology The First Affiliated Hospital of Guangzhou Medical University Guangzhou Medical University Guangzhou 510120 China; ^4^ Department of Burns Nanfang Hospital Southern Medical University Guangzhou 510515 China; ^5^ Department of Stomatology Guangdong Provincial Key Laboratory of Research and Development in Traditional Chinese Medicine Guangdong Second Traditional Chinese Medicine Hospital Guangzhou 510095 China; ^6^ Department of Orthopedics Affiliated Hospital of Putian University Putian 351100 China; ^7^ Guangxi Clinical Research Center for Digital Medicine and 3D Printing Guigang City People's Hospital Guigang 537000 China; ^8^ Biomaterials Research Center School of Biomedical Engineering Southern Medical University Guangzhou 510515 China

**Keywords:** controlled release, extracellular vesicle, logic‐based, osteoarthritis, stimulus‐response

## Abstract

To effectively treat osteoarthritis (OA), the existing inflammation must be reduced before the cartilage damage can be repaired; this cannot be achieved with a single type of extracellular vesicles (EVs). Here, a hydrogel complex with logic‐gates function is proposed that can spatiotemporally controlled release two types of EVs: interleukin 10 (IL‐10)^+^ EVs to promote M2 polarization of macrophage, and SRY‐box transcription factor 9 (SOX9)^+^ EVs to increase cartilage matrix synthesis. Following dose‐of‐action screening, the dual EVs are loaded into a matrix metalloporoteinase 13 (MMP13)‐sensitive self‐assembled peptide hydrogel (KM13E) and polyethylene glycol diacrylate/gelatin methacryloyl‐hydrogel microspheres (PGE), respectively. These materials are mixed to form a “microspheres‐in‐gel” KM13E@PGE system. In vitro, KM13E@PGE abruptly released IL‐10^+^ EVs after 3 days and slowly released SOX9^+^ EVs for more than 30 days. In vivo, KM13E@PGE increased the CD206^+^ M2 macrophage proportion in the synovial tissue and decreased the tumor necrosis factor‐α and IL‐1β levels. The aggrecan and SOX9 expressions in the cartilage tissues are significantly elevated following inflammation subsidence. This performance is not achieved using anti‐inflammatory or cartilage repair therapy alone. The present study provides an injectable, integrated delivery system with spatiotemporal control release of dual EVs, and may inspire logic‐gates strategies for OA treatment.

## Graphical abstract

Osteoarthritis requires a long and stepwise treatment process. In this study, two types of extracellular vesicles (EVs) with anti‐inflammatory and cartilage repair functions are injected into the joint cavity. By using the “matrix metalloproteinase 13 sensitivity” AND “microsphere‐in‐gel” structure of KM13E@PGE delivery materials, the two EVs are spatiotemporal controlled release for macrophages and chondrocytes, achieving an innovative strategy of “anti‐inflammatory first” AND “cartilage repair second” for osteoarthritis treatment.

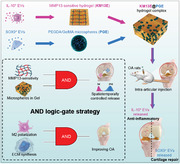

## 1. Introduction

Osteoarthritis (OA) is a prevalent degenerative joint disorder with a range of symptoms, including pain, limited mobility, and physical deformity.^[^ ^1^ ^]^ These symptoms can cause instability and physical disability, ultimately reducing the overall quality of life for the patient. The pharmacological treatment of OA primarily targets the accompanying joint inflammation and cartilage damage via nonsteroidal anti‐inflammatory drugs, corticosteroids, sodium hyaluronate, and glucosamine.^[^ ^2^, ^3^, ^4^ ^]^ The research‐ and clinical treatment‐based consensus is that anti‐inflammatory and cartilage‐repair therapies should not be pursued concurrently.^[^ ^5^, ^6^ ^]^ This recommendation is largely based on the presence of high levels of inflammatory factors,^[^ ^7^ ^]^ such as tumor necrosis factor‐alpha (TNF‐α) and interleukin‐6 (IL‐6). TNF‐α promotes protease activation and continuous dissolution of cartilage collagen in the inflammatory environment, whereas IL‐6 prompts matrix metalloproteinase (MMP) expression following binding to receptors, thereby causing cartilage matrix destruction.^[^ ^8^ ^]^ Within the cells, IL‐1β stimulates the chondrocytes to produce prostaglandin E2 (PGE2), an inhibitor of cartilage matrix synthesis metabolism.^[^ ^9^ ^]^ Therefore, improving the OA inflammatory microenvironment is of primary significance, as this environment directly affects the cartilage destruction behavior and the ultimate curative effect of cartilage repair. In accordance with current regenerative medicine research.^[^ ^10^, ^11^ ^]^ Applications of stem cells, growth factors, and extracellular vesicles (EVs) to OA target one aspect only: either anti‐inflammatory action or cartilage repair. Thus, there is a lack of integrated strategies that follow the AND logic‐gate, as “anti‐inflammatory AND cartilage repair”, and more importantly “anti‐inflammatory first, cartilage repair second”.

EVs offer significant advantages over stem cells for OA therapy. They provide a safer alternative by retaining therapeutic properties while eliminating the risk of tumorigenesis associated with stem cell transplantation.^[^ ^12^, ^13^, ^14^ ^]^ Moreover, EVs allow for improved targeting, ease of storage, and administration, making them promising candidates for the development of effective and scalable OA treatments.^[^ ^15^ ^]^ Mesenchymal stem cell‐derived EVs (MSC‐EVs) may stimulate chondrocyte matrix synthesis, or activate stem cells during OA repair.^[^ ^16^ ^]^ In the context of anti‐inflammatory therapy, EVs rich in anti‐inflammatory cytokines, such as IL‐10 and IL‐6, may serve as macrophage M2 polarization switchers.^[^ ^17^ ^]^ Furthermore, EVs from the macrophage cell line RAW264.7 may enhance the targeted therapeutic effects of immune regulation by promoting internalization by macrophages via integrin interaction.^[^ ^17^, ^18^ ^]^ In the context of cartilage repair, MSC‐EVs and platelet‐rich plasma EVs can encourage chondrocyte proliferation, promote chondrocyte matrix synthesis, and reduce joint cartilage degeneration.^[^ ^19^ ^]^ While infrapatellar fat pad‐derived mesenchymal stem cells (IPFP‐MSCs) located near the OA joint may also contribute to disease development, their EVs, which are rich in proteoglycan 4 (PRG4) and microRNA (miR)−100‐5p, can potentially support cartilage repair.^[^ ^20^ ^]^ Although EVs have multiple roles, a certain type of EV may possess relatively singular therapeutic effects and may require appropriate engineering or specific cultural environments for activation of their therapeutic effects.^[^ ^21^ ^]^ Considering the Boolean “AND” logic‐gates therapeutic function required for OA, which targets first anti‐inflammatory action and then cartilage repair, the use of at least two types of EVs may be necessary. And obviously, it is challenging to deliver two types of EVs at different times and in different areas.

Intravenous injection of EVs usually yields a low EV concentration within the joint, owing to the relatively closed anatomical structure of the joint cavity.^[^ ^5^ ^]^ However, injection into the joint cavity ensures local, high‐concentration EV enrichment.^[^ ^22^ ^]^ Sodium hyaluronate and chitosan are commonly used for joint‐cavity injections in clinical settings.^[^ ^5^, ^23^ ^]^ Hydrogels with similar properties may serve as excellent EV‐delivery materials by providing a physical buffer to the joint cavity.^[^ ^2^ ^]^ High‐water‐content materials prevent damage to EVs and ensure a higher EV load.^[^ ^2^, ^24^ ^]^ Self‐assembling peptides (SAPs) are short peptides that spontaneously form beta folds based on specific amino acid sequences.^[^ ^25^ ^]^ These peptides form hydrogels at the macroscopic scale, enabling the insertion of functional peptides; this aspect renders them useful as hydrogels for biological response strategies.^[^ ^26^ ^]^ In cases of OA, the level of matrix metalloproteinase 9 (MMP9) and MMP13 increase within the joint cavity.^[^ ^27^ ^]^ The incorporation of enzyme‐substrate sequences into SAPs enables their breakdown in response to the MMP9‐ and MMP13‐containing inflammatory environment, thereby controlling the release of the drugs they contain. Our previous research highlighted the use of MMP1‐sensitive SAP in clinical treatment.^[^ ^28^ ^]^ The ability of MMP1‐sensitive SAP to respond to neovascularization and regulate exosome release spatially and temporally offers a novel strategy for controlled release. In the context of OA pathology, MMP13‐sensitized hydrogels can initiate drug release and achieve an “anti‐inflammatory first” effect. Challengingly, the “cartilage repair second” effect needs to be combined with another system with differential release rates.

**Scheme 1:**
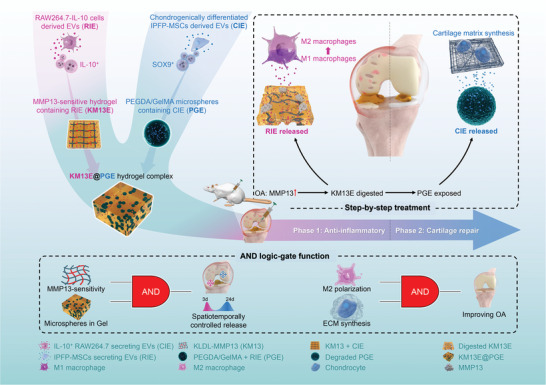
Schematic illustration: the KM13E@PGE system spatiotemporally releases of two types of EVs according to its MMP13‐sensitive characteristics and “microspheres‐in‐gel” structure, resulting in step‐by‐step efficacy of anti‐inflammatory and cartilage repair to improve OA.

In this study, we construct an MMP13‐sensitive hydrogel complex that responds to the OA inflammatory environment and releases anti‐inflammatory EVs as an initial step, followed by cartilage‐repairing EVs. In detail, we genetically engineer IL‐10^+^ EVs (RIE) for anti‐inflammatory treatment and induce SOX9^+^ EVs (CIE) for cartilage repair. The two EVs are packed in SAPs and microspheres, respectively, which are combined to form a composite “microspheres‐in‐gel” system. Due to the different release rates of the two EVs in the hydrogel complex, and the effect of the two EVs’ different targeting properties, spatiotemporal controlled release of the drug was achieved. This hydrogel complex can be injected directly into the joint cavity with minimal invasiveness. The anti‐inflammatory RIE is released in response to MMP13. Slow release of the CIE then occurs, which promotes cartilage repair. Hence, step‐by‐step, integrated treatment of OA is enabled via a new type of logic‐based material that is jointly controlled by biological responses and its physical structure.(**Scheme** **1**).

## 2. Results

### 2.1. IL‐10 Overexpression within EVs Enhances Anti‐Inflammatory Capabilities

**Figure 1:**
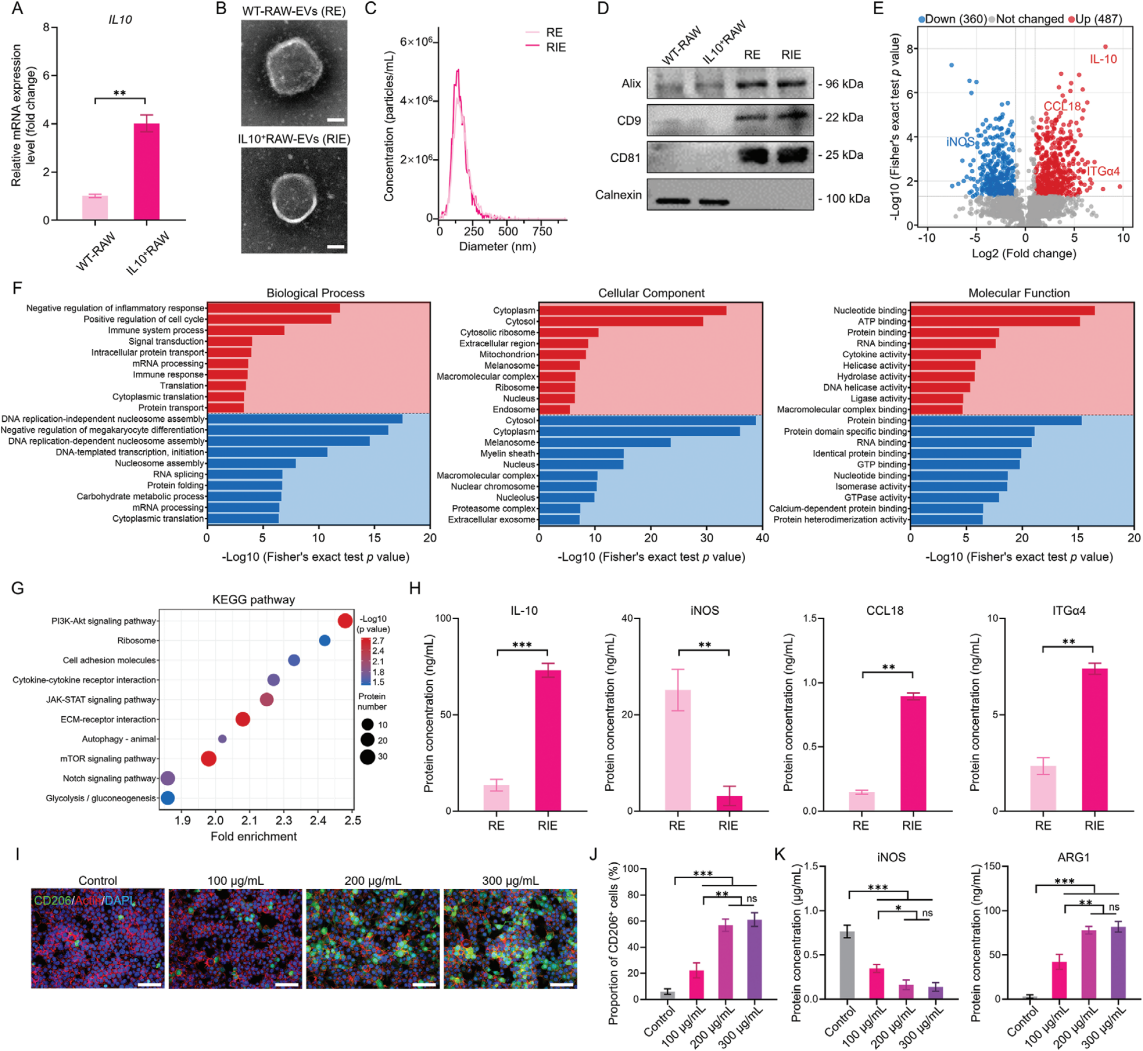
RIE characterization and screening of optimal RIE concentrations. A) IL‐10 mRNA expression in RAW264.7 before and after transfection. B) EV morphologies observed via transmission electron microscopy (TEM); scale bar: 50 nm. C) EV particle size distribution measured via nanoparticle tracking analysis (NTA). D) Western blotting of EV markers. E) DEP volcano map between RIE and RE; *p* < 0.05; log_2_ fold change. F,G) GO enrichment and KEGG pathway analyses, respectively, of DEP between RIE and RE. H) Inflammation‐related DEP quantified via ELISA, for RE and RIE. For M1‐polarized RAW264.7 samples treated with various RIE concentrations for 24 h: I) CD206 fluorescent staining of samples; scale bar: 100 µm; J) CD206^+^ cell percentages following CD206 fluorescent staining; and K) M1 polarization‐related protein iNOS and M2 polarization‐related protein ARG1 concentrations, quantified by ELISA. ^***^: *p* < 0.001, ^**^: *p* < 0.01, ^*^: *p* < 0.05, ns: *p* > 0.05.

To increase the IL‐10 content within the EVs, we overexpressed IL‐10 in the RAW264.7 macrophage cell line by using a lentiviral vector (Figure S1, Supporting Information) to increase the IL‐10 mRNA (**Figure** **1**A). We obtained EVs produced with WT‐RAW (RE) and IL‐10^+^ RAW (RIE) from a cell culture medium. Both the RE and RIE had cup‐shaped morphologies (Figure 1B) with particle sizes within the 80–150 nm range (Figure 1C). No significant difference in protein concentration was determined for the RE and RIE secretion levels (Figure S2A, Supporting Information). Both the RE and RIE were highly enriched with CD9, CD81, and ALIX proteins, which are well‐known EV markers (Figure 1D). Therefore, we successfully isolated RE and RIE. To clarify the impact of the IL‐10 overexpression on the RIE protein composition, protein profiling was performed: 1101 proteins were detected in the RIE and RE, of which 360 and 487 were down‐ and upregulated, respectively, by the log_2_ fold change (Figure 1E). Gene ontology (GO) analysis revealed that the biological processes of the differentially expressed proteins (DEP) were primarily inflammatory and cytokine regulation and that the DEP cellular components and molecular functions were primarily associated with extracellular proteins and adenosine triphosphate (ATP) binding (Figure 1F). Kyoto Encyclopedia of Genes and Genomes (KEGG) pathway analysis revealed that the phosphoinositide 3‐kinase–protein kinase B (PI3K‐Akt) signaling pathway was the most significant (Figure 1G). These results demonstrate that RIE significantly influences anti‐inflammation activity. Furthermore, we quantified the DEP via an enzyme‐linked immunosorbent assay (ELISA); the results showed significantly higher IL‐10, C─C motif chemokine ligand 8 (CCL18), and integrin subunit alpha 4 (ITGα4) levels in the RIE compared to the RE. The nitric oxide synthase (iNOS) level was significantly lower, and no significant differences were obtained for ITGβ1, ITGβ2, and ITGαM (Figure 1H; Figure S2B, Supporting Information).

Following the co‐culturing of the RIE with M1‐polarized RAW264.7 cells in vitro, immunofluorescence analysis was performed; the results showed that RIE concentrations exceeding 200 µg mL^−1^ significantly reversed the M1 polarization, yielding a significant amount of CD206^+^ M2 polarization (Figure 1I,J). ELISA results also confirmed this finding, as the iNOS decreased with increasing RIE concentration and was negatively correlated with the M2 polarization. In contrast, the Arginase‐1 (ARG1) expression was positively correlated with the M2 polarization and increased with increasing RIE concentration. Regarding the effective RIE dose, there was no significant difference between 200 and 300 µg/mL (Figure 1K). These results indicate the successful construction of IL‐10^+^ EVs promoting macrophage M2 polarization, that is, RIE.

### 2.2. Chondrogenically Differentiated IPFP‐MSC‐Derived EVs Promote Cartilage Matrix Synthesis

**Figure 2:**
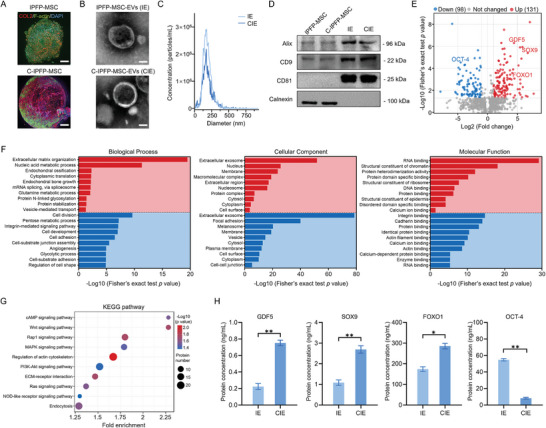
CIE characterization. A) COL2 fluorescence staining of IPFP‐MSC and C‐IPFP‐MSC chondrospheres over 14 d; scale bar: 1 mm. B) EV morphologies observed via TEM; scale bar: 50 nm. C) EV particle size distribution measured via NTA. D) Western blotting of EV markers. E) DEP volcano map between IE and CIE; *p* < 0.05; log_2_ fold change. F,G) GO enrichment and KEG pathway analyses, respectively, of DEP between IE and CIE. H) Chondrogenesis‐related DEP quantified via ELISA. ^**^: *p* < 0.01, ^*^: *p* < 0.05.

Through enzymatic digestion of infrapatellar adipose tissue discarded during arthroscopic surgery, we successfully obtained IPFP‐MSCs (Figure S3A,B, Supporting Information). These cells showed a strong preference for chondrogenic differentiation (Figure S3C, Supporting Information). Immunofluorescence analysis showed that the chondrogenically differentiated IPFP‐MSCs (C‐IPFP‐MSCs) formed dense chondrospheres rich in type‐II collagen (COL2), as shown in **Figure** **2**A. We extracted IPFP‐MSC‐derived EVs (IE) and C‐IPFP‐MSC‐derived EVs (CIE) from a cell culture medium soaked with chondrospheres. Both the IE and CIE assumed cup‐shaped morphologies (Figure 2B) with particle sizes in the range of 90 to 140 nm (Figure 2C). There were no statistically significant differences in the IE and CIE secretion levels (Figure S4, Supporting Information). Both the IE and CIE expressed the CD9, CD81, and ALIX EV markers, but did not express calnexin (Figure 2D). To further clarify the impact of chondrogenic differentiation on the CIE protein components, protein profiling was performed. As shown in the volcano plot (Figure 2E), 521 proteins were totally quantified in the CIE and IE groups, of which 98 downregulated and 131 upregulated, DEP in the CIE group, compared with the IE group, by the log_2_ fold change. GO analysis revealed that the DEP were significantly enriched by the regulation of the cellular chondrogenic differentiation and the extracellular matrix (ECM) secretion, with the cellular components and molecular functions primarily involving extracellular proteins and RNA binding (Figure 2F). Moreover, KEGG pathway enrichment analysis suggested that the wingless (Wnt) and cyclic adenosine monophosphate (cAMP) signaling pathways were the most DEP‐related (Figure 2G). We performed ELISA to validate the growth differentiation factor 5 (GDF5), SOX9, proteoglycan 4 (PRG4), and POU class 5 homeobox 1 (OCT‐4) protein contents, which were related to the stem cell fate; the quantitative results were consistent with the GO‐analysis expression trend (Figure 2H). The above results indicate successful isolation of IE and CIE, and that the CIE obtained after chondrogenic differentiation was rich in GDF5, forkhead box O1 (FOXO1), and SOX9, which have potential cartilage matrix synthesis functionality.

**Figure 3:**
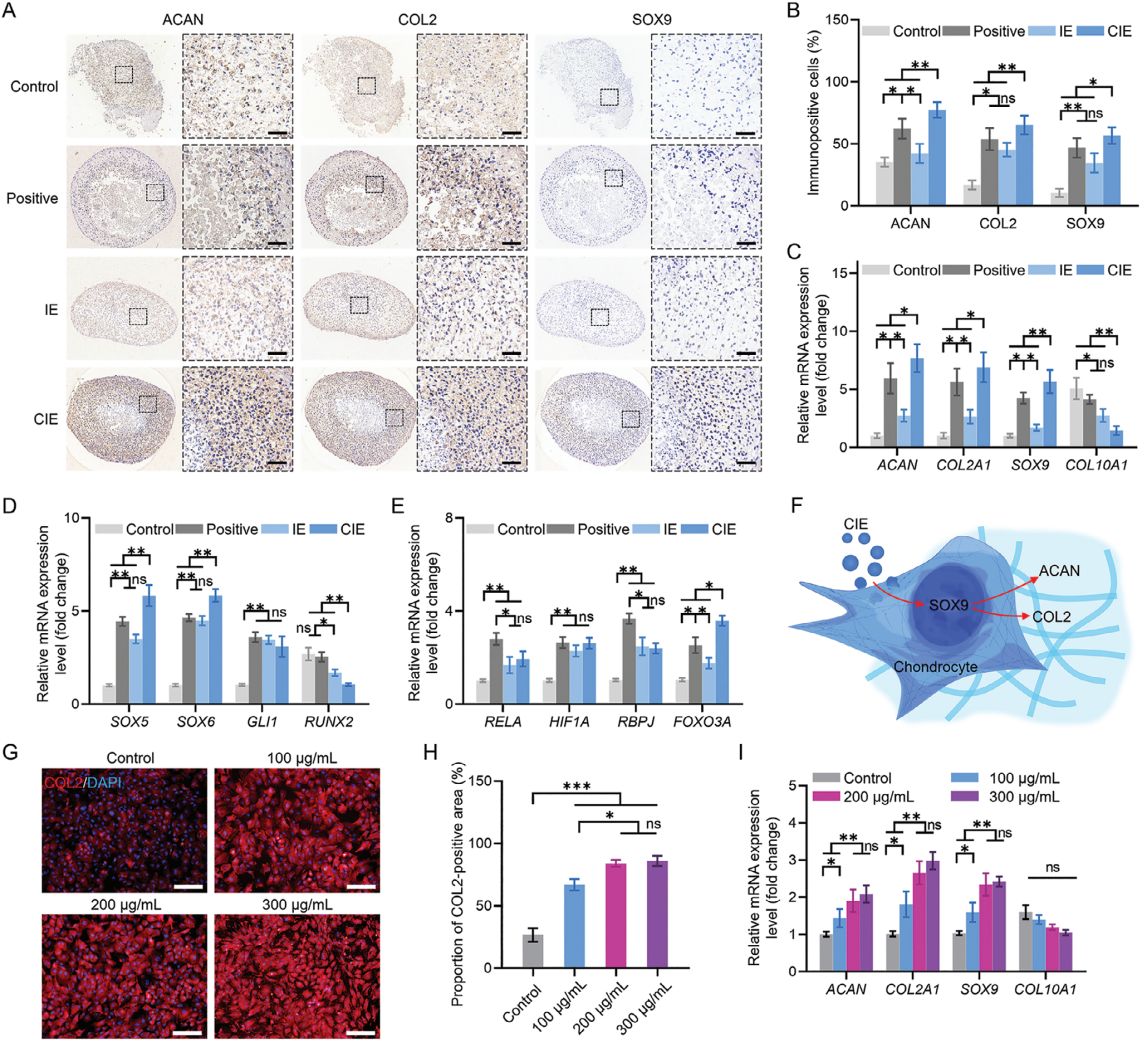
CIE concentration screening and cartilage matrix‐synthesis promotion mechanism. Groups: Control: proliferation medium; Positive: chondrogenic differentiation medium; IE: proliferation medium containing 300 µg mL^−1^ IE; CIE: proliferation medium containing 300 µg mL^−1^ CIE. A) Immunohistochemical staining of ACAN, COL2, and SOX9 at 21 d; Magnification: 50× (left) and 200× (right); scale bar: 50 µm. B) Proportions of cells with positive immunohistochemical staining. C) *ACAN*, *COL2A1*, *SOX9*, and *COL10A1* mRNA expression levels. D) *SOX9*‐related gene expression levels: *SOX5*, *SOX6*, *GLI1*, and *RUNX2*. E) *SOX9* upstream transcription‐factor expression levels: *RELA*, *HIF1A*, *RBPJ*, and *FOXO3A*. F) Schematic diagram of CIE‐induced cartilage matrix‐synthesis promotion mechanism. For C28/I2 treated with various CIE concentrations for 48 h: G) COL2 fluorescence staining samples; scale: 100 µm; H) COL2^+^ cell percentages following COL2 fluorescent staining; and I) *ACAN*, *COL2A1*, *SOX9*, and *COL10A1* mRNA expression levels via RT‐qPCR. ^***^: *p* < 0.001, ^**^: *p* < 0.01, ^*^: *p* < 0.05, ns: *p* > 0.05.

To further clarify the CIE ability to promote cartilage matrix synthesis, we co‐cultured CIE cells with chondrocytes (the C28/I2 cell line) to form chondrospheres in vitro. Four groups were considered: a proliferation medium, a chondrogenic differentiation medium, a proliferation medium containing 300 µg mL^−1^ IE, and a proliferation medium containing 300 µg/mL CIE. Immunohistochemical staining revealed that the CIE‐induced chondrospheres had the highest expression of aggrecan (ACAN), COL2, and SOX9 proteins among all groups, surpassing the chondrogenically differentiated group (**Figure** **3**A,B). Similarly, regarding the mRNA expression levels, the highest *ACAN*, *COL2A1*, and *SOX9* expression levels were obtained for the CIE group, along with the lowest levels of the hypertrophic cell marker *COL10A1* (Figure 3C). This outcome suggests that CIE not only delivers SOX9 protein to chondrocytes but also promotes *SOX9* mRNA self‐expression in target cells. We further detected *SOX5* and *SOX6*, which functionally interacted with the *SOX9* and were highly expressed in the CIE group (Figure 3D). Following co‐cultivation with CIE, no significant change in the *SOX9* downstream target GLI family zinc finger 1 (*GLI1*) was obtained. Another downstream target related to osteogenic differentiation, *RUNX* family transcription factor 2 (*RUNX2*), decreased significantly. To clarify the *SOX9* cellular self‐expression pathway, the following upstream transcription factors were detected: RELA proto‐oncogene (*RELA*), hypoxia‐inducible factor 1 subunit alpha (*HIF1A*), the recombination signal binding protein for the immunoglobulin Kappa J region (*RBPJ*), and *FOXO3A*. Only the mRNA expression of *FOXO3A* was significantly enhanced (Figure 3E). This result suggests that *FOXO1*, carried by the CIE, activated *SOX9* expression in the target cells through the *FOXO1–FOXO3A/SOX9* pathway. Under the effects of direct SOX9 delivery and stimulation of endogenous *SOX9* expression through FOXO1 delivery, the CIE promoted chondrocyte synthesis in the ECM (Figure 3F).

We investigated the CIE chondrocyte internalization efficiency of chondrocytes via fluorescence imaging, obtaining absorption peaks at 48 h (Figure S5, Supporting Information). Therefore, we co‐cultured different CIE concentrations with C28/I2 cells for 48 h and screened for the optimal CIE concentration. Comparable induction levels were obtained for 200 and 300 µg mL^−1^ CIE concentrations, significantly elevating the expression of COL2, a cartilage marker (Figure 3G,H). This finding was confirmed using the quantitative polymerase chain reaction with reverse transcription (RT‐qPCR) method (Figure 3I).

### 2.3. Characteristics of MMP13‐Sensitive Hydrogel

**Figure 4:**
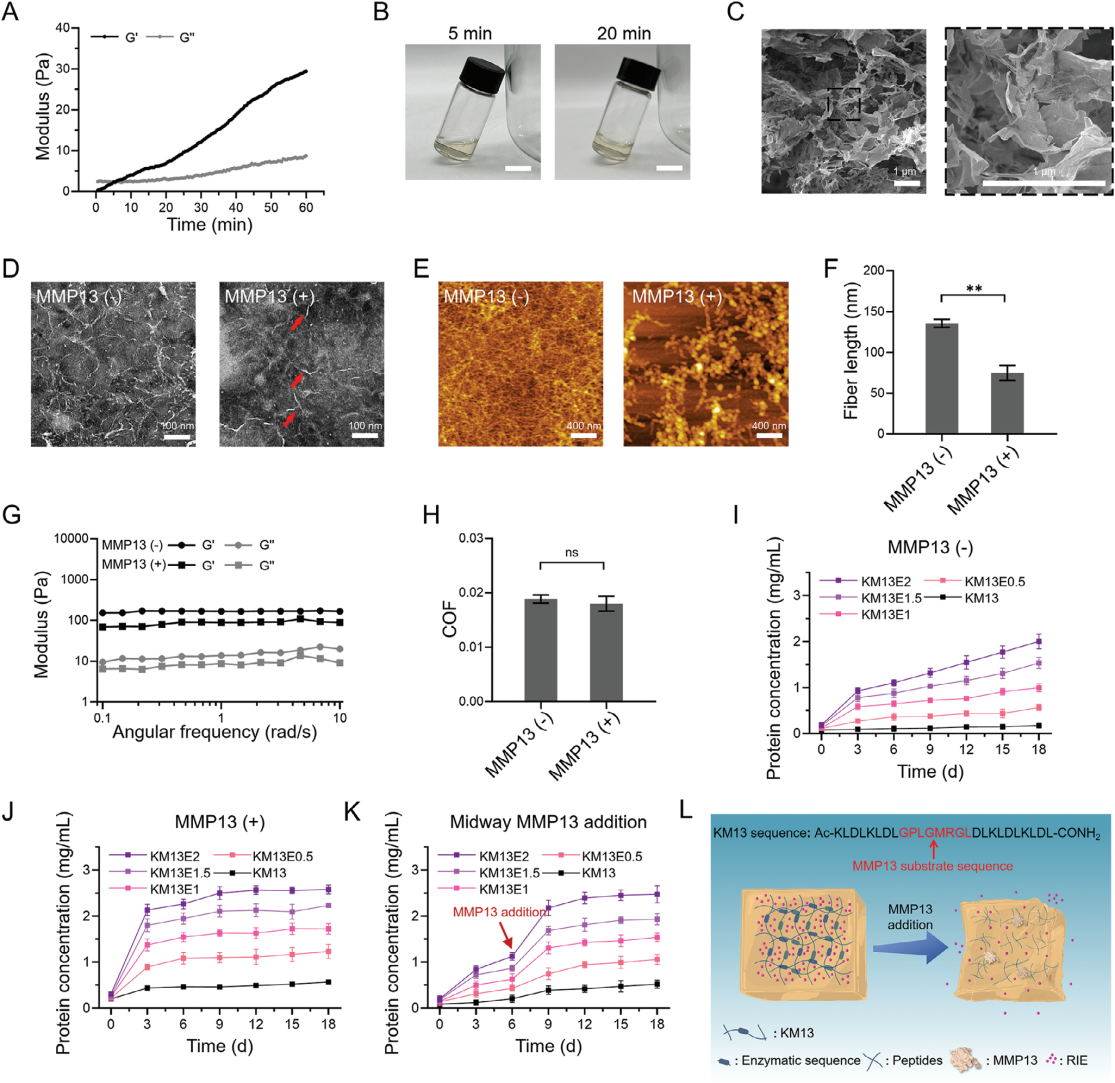
MMP13‐sensitive hydrogel (KM13) characterization. A) Gelation profile of 1% (w/v) KM13 over 60 min. B) Image of 1% (w/v) KM13 dissolved in DPBS for 5 and 20 min at room temperature; scale bar: 2 cm. C) SEM micrographs of 1% (w/v) KM13 hydrogel; scale bar: 1 µm. D) TEM and E) AFM micrographs of 1% (w/v) KM13 nanofibers before and after digestion by MMP13; scale bars: 100 and 400 nm, respectively. F) 1% (w/v) KM13‐nanofiber lengths before and after digestion by MMP13. G) Storage and loss moduli (G’ and G’’, respectively) of KM13 before and after digestion by MMP13 for angular frequencies of 0.1–10 rad/s (1% strain). (H) COF of 1% (w/v) KM13 hydrogel before and after digestion by MMP13. BCA assays of KM13E containing different RIE concentrations (0, 0.5, 1, 1.5, 2 mg mL^−1^) in (I) SBF solution, (J) MMP13 solution (SBF containing 5 ng mL^−1^ MMP13), and K) SBF solution (with MMP13 addition at 6 d, to a final concentration of 5 ng mL^−1^), over 18 d. (L) Schematic diagram of KM13E releasing RIE in response to MMP13. ^**^: *p* < 0.01, ns: *p* > 0.05.

An MMP13‐sensitive hydrogel was used as an RIE carrier, based on the insertion of the MMP13 substrate sequence into a KLDL‐12 self‐assembled peptide. The molecular weight of KLDL‐MMP13 (KM13) is 2.71 kDa, and its theoretical isoelectric point is 7.01. Rheological tests demonstrated a gelation time of 5 min for 1% (w/v) KM13 (**Figure** **4**A). In a gel‐formation experiment, KM13 formed a solid hydrogel block after 20 min (Figure 4B). The above characteristics of KM13 indicate that it can spontaneously but slowly change from a solution to a hydrogel. Scanning electron microscope (SEM) images confirmed that the KM13 hydrogel had a 3D porous structure (Figure 4C).

We observed the enzymatic response degradation of KM13 via TEM and atomic force microscopy (AFM), in a simulated body fluid (SBF) solution. For 1% (w/v) KM13, interwoven nanofibers were observed. We measured the intra‐articular MMP13 content and activity in the joint‐fluid of OA rats, and used a consistent 4.78 ng mL^−1^ of MMP13 to prepare the enzymatic solution (Figure S6A,B, Supporting Information). In the MMP13 solution, the KM13 nanofiber structure was broken and loosely arranged (Figure 4D,E). Quantitative analysis revealed that the KM13 nanofiber length before contact with MMP13 was 135.71 ± 13.64 nm; after enzymatic digestion, the nanofiber length decreased significantly to 74.89 ± 24.38 nm (Figure 4F). We also observed a significant decrease in the KM13‐hydrogel modulus after enzymatic digestion; however, their coefficient of friction (COF) values remained relatively unchanged (Figure 4G,H). Notably, these enzymatically digested KM13 gels exhibited modulus and COF values similar to those of commercially available hyaluronic acid, indicating that KM13 gels may be a viable option for intra‐articular injection (Figure S6C,D, Supporting Information). In addition, the shear modulus of the KM13 hydrogels did not differ significantly before and after enzymatic digestion, demonstrating that they remained well stable at high MMP13 levels (Figure S6E, Supporting Information). To confirm that KM13 could be extruded smoothly through a syringe, we also tested its shear‐thinning properties (Figure S7, Supporting Information). The drug release rates of KM13 samples mixed with various RIE concentrations (0.5–2 mg mL^−1^, labeled KM13E0.5, KM13E1, KM13E1.5, and KM13E2, respectively), and of KM13 without RIE, were evaluated by quantifying the protein concentration released into the solution. Without MMP13, KM13E released all the RIE within 18 d (Figure 4I; Figure S8A, Supporting Information). Of the various samples, the KM13E1.5 average release concentration was closest to 200 µg mL^−1^ (Figure S8B, Supporting Information). In the presence of MMP13, a sudden RIE release was observed, with complete release occurring within 9 d (Figure S8C, Supporting Information). Note that KM13 also released protein components when digested by MMP13 (Figure 4J). Again, the KM13E1.5 average release concentration was the closest group to 200 µg mL^−1^ (Figure S8D, Supporting Information). Midway addition of MMP13 significantly increased the KM13E protein release rate (Figure 4K; Figure S8E,F, Supporting Information). In DMEM medium, KM13E also released all RIE within 18 d and the release profile was similar to that in SBF solution without MMP13 enzyme (Figure S9, Supporting Information). We also considered some of the MMPs expressed in the OA joint fluid, including MMP1, MMP3, and MMP9. The KM13 hydrogel were specifically responsive to MMP13 (Figure S10, Supporting Information). These results show that the KM13 hydrogel can be degraded by MMP13 in particular to rapidly release RIE, while maintaining certain structural and mechanical properties following enzymatic degradation (Figure 4L).

### 2.4. Droplet‐Based Microfluidics Facilitating Microsphere Formation

**Figure 5:**
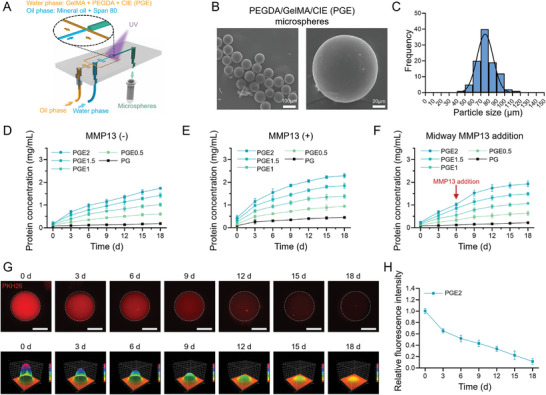
PGE microsphere characterization. A) Schematic diagram of microfluidic focusing chip used for PGE microsphere preparation. B) SEM micrographs showing uniform spherical PGE microspheres. C) Histogram of PGE‐microsphere diameter distribution. BCA assays of PGE microspheres in (D) SBF solution, (E) MMP13 solution (SBF containing 5 ng mL^−1^ MMP13), and (F) SBF solution (with MMP13 addition at 6 d, to a final concentration of 5 ng mL^−1^), over 18 d. G) Immunofluorescence images of single PGE2 microsphere (the CIE were labeled with PKH26) in SBF solution over 18 d; scale bar: 50 µm. J) Quantified relative fluorescence intensity of single PGE2 microsphere.

Polyethylene glycol diacrylate/gelatin methacryloyl hydrogel (PEGDA/GelMA) microspheres were used as CIE carriers. The synthesized GelMA was first examined via proton nuclear magnetic resonance analysis; a clear characteristic peak was observed (Figure S11A, Supporting Information). The GelMA was then mixed with PEGDA to form gels and examined via Fourier‐transform infrared spectroscopy, which confirmed the successful formation of PEGDA/GelMA (PG) hydrogels (Figure S11B, Supporting Information). The storage and loss moduli (G’ and G’’, respectively) of the PG hydrogels exhibited slight fluctuations and low strain dependence (Figure S11C, Supporting Information). **Figure** **5**A is a schematic of the microfluidic focusing chip. The oil and water phase solutions intersected to form water droplets in the oil. Following ultraviolet curing, the materials in the water phase formed a stable 3D structure, thus maintaining the microsphere structures. SEM images showed that the PEGDA/GelMA/CIE (PGE) microspheres prepared using the microfluidic focusing chips had a consistent size and smooth surfaces (Figure 5B). The microsphere sizes were predominantly within the 70–80‐µm range, with an average diameter of 75.172 ± 13.682 µm (Figure 5C).

To investigate the controlled release effect of the PGE microspheres in the MMP13 solution, we prepared microspheres with CIE concentrations of 0.5–2 mg mL^−1^ (labeled PGE0.5, PGE1, PGE1.5, and PGE2, respectively), and also considered PG without CIE. In the absence of MMP13, all the PGE groups released ≈90% of their CIE within 18 d (Figure 5D; Figure S12A, Supporting Information). Among the various samples, the PGE2 average release concentration was closest to 200 µg mL^−1^ (Figure S12B, Supporting Information). In the presence of MMP13, the release rates of all PGE groups increased from 50% to 70%. Here, the PG may have been digested by the MMP13, increasing the protein release concentration of the PG group (Figure 5E; Figure S12C, Supporting Information). We speculate that this phenomenon is caused by the degradation of gelatin components in GelMA by MMP13. However, this sudden release effect was not as significant as that of KM13E (40–90% release rate). Again, the PGE2 average release concentration was closest to 200 µg mL^−1^ (Figure S12D, Supporting Information). Midway addition of MMP13 enhanced the PGE protein release rate (Figure 5F; Figure S12E,F, Supporting Information), but not as significantly as for the KM13E response to MMP13. Confocal microscopy was used to observe the release of PKH26‐labeled CIE from single microsphere over 18 d. The PGE2‐group fluorescence intensity gradually decreased over time, indicating CIE release (Figure 5G). Quantitative analysis of the fluorescence intensity showed that the PGE2 retained weak fluorescence until Day 18, when the CIE was completely released (Figure 5H).

### 2.5. The “Microspheres‐In‐Gel” Structure Leads to Differential Release Rates of the Two EVs

**Figure 6:**
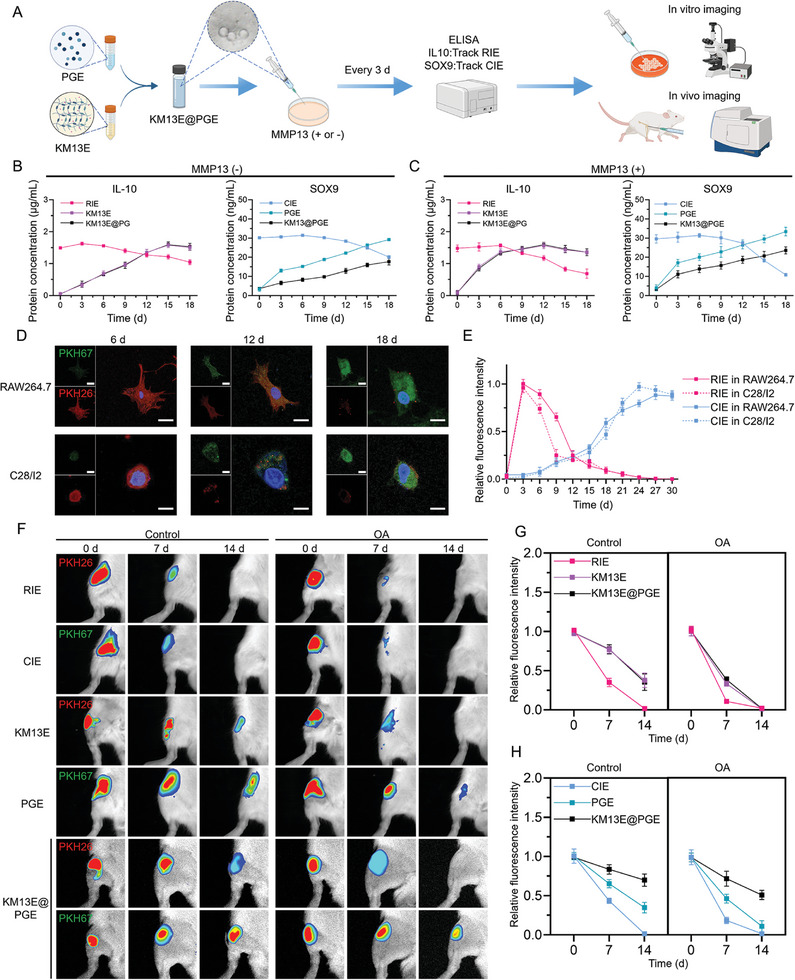
Controlled release of KM13E@PGE in vitro and in vivo. A) Schematic diagram of KM13E@PGE controlled‐release detection. B,C) ELISA assay of KM13E@PGEs in SBF and MMP13 (SBF containing 5 ng mL^−1^ MMP13) solutions, respectively, over 18 d. D,E) Immunofluorescence images and quantitative fluorescence intensity analysis results, respectively, for PKH26‐labeled RIE and PKH67‐labeled CIE uptake by RAW264.7 and C28/I2 within 30 d; scale bar: 20 µm. F) In vivo imaging at 0, 7, and 14 d post‐injection into rat joint cavities. G,H) Quantification of KM13E@PGE relative fluorescence intensity in vivo, for RIE and CIE, respectively.

Following the construction of RIE‐containing KM13E hydrogel and CIE‐containing PGE microspheres, we expected that the “microspheres‐in‐gel” hydrogel complex, that is, KM13E@PGE, could achieve different release rates for the two EVs. We performed quantitative analysis of IL‐10 and SOX9 to assess the released RIE and CIE, respectively. Based on the separately marked fluorescence of the two EVs, the KM13E@PGE was co‐cultured with macrophages and chondrocytes in vitro and injected into joint cavities in vivo. Hence, the differential release rates of the two EVs was examined for KM13E@PGE (**Figure** **6**A). ELISA results showed that, regardless of the presence of MMP13, the IL‐10 and SOX9 concentrations in the pure EVs (both RIE and CIE) decreased continuously, indicating the possibility of EV degradation or MMP13 enzymatic hydrolysis. In terms of IL‐10 release, there was no significant difference between the KM13E and KM13E@PG groups, as the KM13E was located in the outer layer of the “microspheres‐in‐gel” system. However, with the involvement of MMP13, both the KM13E and KM13E@PG groups exhibited significantly increased IL‐10 releases. The difference in their performance pertained to their SOX9 release. The PGE and KM13@PGE groups exhibited more sustained release rates, which was due to the PGE encapsulation performed by the KM13 in the “microspheres‐in‐gel” system. In the presence of MMP13, the SOX9 releases of the PGE and KM13@PGE groups were slightly upregulated, possibly because of the enzymatic hydrolysis of the GelMA component in the PGE (Figure 6B,C). These results indicate that the enzyme‐sensitive hydrogel outer layer of KM13E@PGE can rapidly release RIE in the presence of MMP13. However, MMP13 does not significantly affect the CIE release rate from the KM13E@PGE system.

We labeled the RIE and CIE with PKH26 and PKH67, respectively, and added them to the KM13E@PGE system. Direct observation of the fluorescence intensity in the KM13E@PGE material was not feasible because the dispersed PKH26‐labeled RIE was easily mis‐identified as background fluorescence. However, we observed changing fluorescence trends for the two types of EVs (Figure S13A,B, Supporting Information). Therefore, we co‐cultured KM13E@PGE with RAW264.7 and C28/I2, respectively, to facilitate observation of the fluorescence intensities after the EVs were internalized in the cells. The results showed that both cell types first absorbed the PKH26‐labeled RIE. Then, as the fluorescence carried by the RIE gradually weakened, the PKH67 fluorescence carried by the CIE gradually increased (Figure 6D). Quantitative analysis showed that the fluorescence peaks of RAW264.7‐ and C28/I2‐internalized RIE appeared on Day 3. The fluorescence peak of the RAW264.7‐internalized CIE appeared on Day 27, whereas that for the C28/I2 appeared on Day 24 (Figure 6E). This result indicates that the proposed KM13E@PGE system has a programmable release function in which RIE is released first, followed by CIE.

Because of the inability of in vivo imaging to distinguish between the two fluorescent markers, we separately labeled a single EV type with fluorescence in our in vivo experiments. The results showed that, for direct injection of RIE or CIE into the joint cavities of wild‐type rats, the EVs could not be maintained for 14 d. Moreover, the fluorescence had decreased by more than 50% on Day 7. In OA rats, the fluorescence nearly disappeared on Day 7 (Figure 6F–H). When KM13E or PGE were injected into wild‐type rats, the EV fluorescence persisted for up to 14 d. However, in the joints of the OA rats, less than 50% of the fluorescence intensity of KM13E and PGE remained at 7 d, and had almost disappeared at 14 d (Figure 6F–H). For KM13E@PGE, the fluorescence in the wild‐type rats was maintained for 14 d; at that time, the RIE and CIE fluorescence had decreased by ≈50% and ≈30%, respectively. However, in OA rats, the RIE fluorescence had decreased by 60% by Day 7 and had disappeared completely by Day 14. The CIE fluorescence was maintained for 14 d, with the fluorescence decreasing by ≈40%; however, there was no significant difference in the trend between the OA rats and wild‐type rats (Figure 6F,H). This outcome further demonstrates the sensitivity of KM13E to OA biochemical indicators, and that the KM13E@PGE system quickly released RIE within 7 d and slowly released CIE for more than 14 d. In summary, the MMP13‐sensitive characteristics and “microspheres‐in‐gel” structure of KM13E@PGE contribute to the different release rates of RIE and CIE. Additionally, the homologous targeting effect of EVs and the relatively active immune cells during the early stages of injection collectively result in a spatiotemporal controlled release effect of dual EVs (Figure S14, Supporting Information).

### 2.6. Anti‐Inflammatory Effect of KM13E@PGE In Vivo

**Figure 7:**
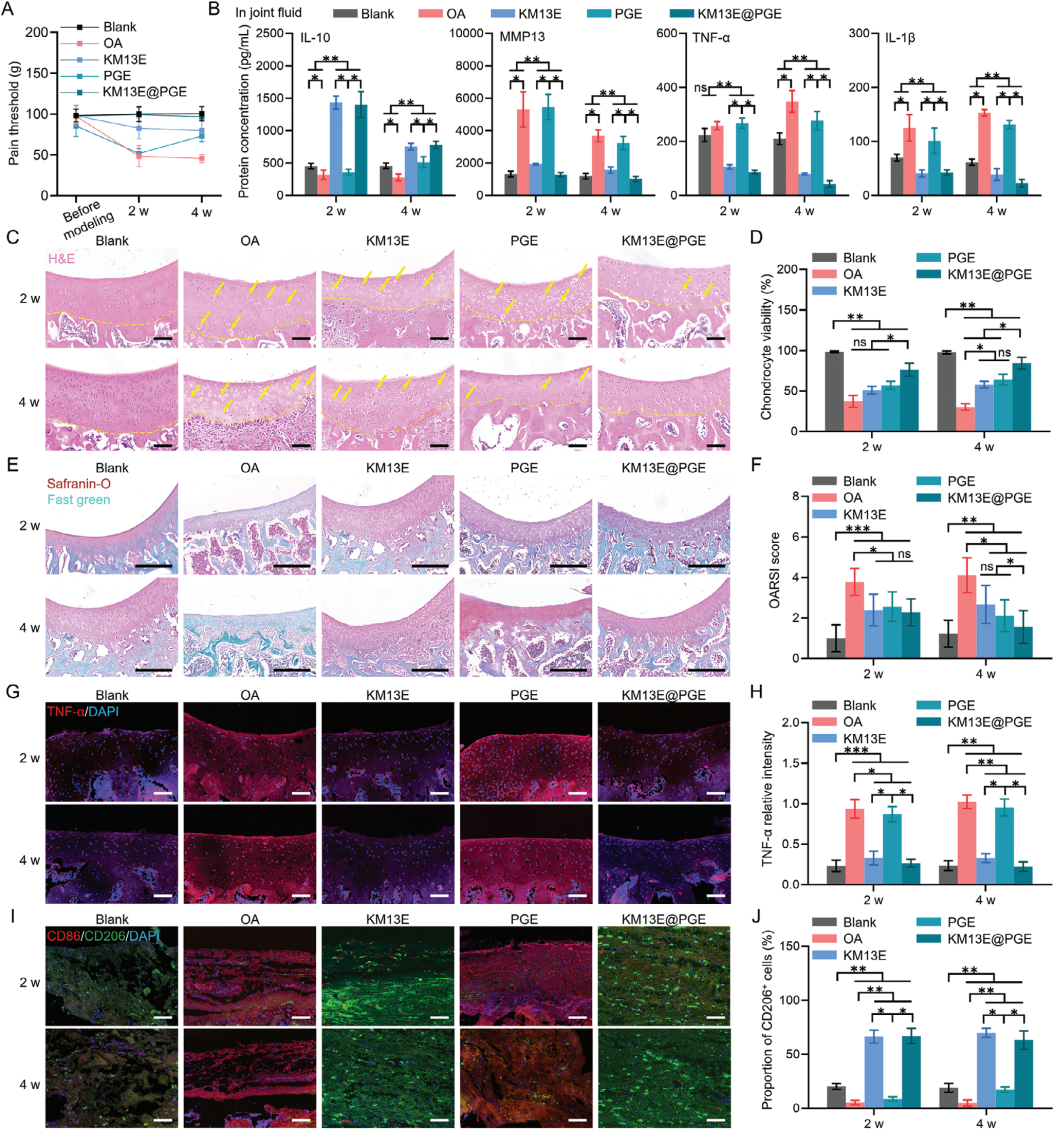
KM13E@PGE‐induced improvement of the joint‐cavity inflammatory microenvironment in OA rats. Groups: Blank: saline injection without modeling; OA: MIA injection modeling for 2 w; KM13E: KM13E (containing 1.5 mg mL^−1^ RIE) hydrogel injection; PGE: PGE (containing 2 mg mL^−1^ CIE) microsphere injection, KM13E@PGE: KM13E@PGE (containing 1.5 mg mL^−1^ RIE and 2 mg mL^−1^ CIE) injection. A) Plantar pain threshold analysis results, before modeling, 2 and 4 w after modeling. B) ELISA detection of IL‐10, MMP13, TNF‐α, and IL‐1β concentrations in joint fluid. C) H&E staining of articular cartilage tissue at 2 and 4 w; scale bar: 200 µm. The yellow arrows indicate cartilage traps that have lost chondrocytes; the yellow dotted lines distinguish cartilage layers. D) Chondrocyte viability counted via H&E staining. E) Safranin O/fast‐green staining of articular cartilage tissue at 2 and 4 w; scale bar: 1 mm. F) ORASI score statistics based on H&E staining and Safranin O/fast‐green staining. G) TNF‐α fluorescent staining of articular cartilage tissue at 2 and 4 w; scale bar: 200 µm. H) Quantification of TNF‐α relative fluorescence intensity. I) CD86/CD206 fluorescent staining of synovial tissue at 2 and 4 w; scale bar: 200 µm. J) CD206^+^‐cell proportions following CD86/CD206 fluorescent staining. ^***^: *p* < 0.001, ^**^: *p* < 0.01, ^*^: *p* < 0.05, ns: *p* > 0.05.

Plantar pain threshold analysis was performed for four groups, which received the following treatment: saline injection without modeling (Blank group); monosodium iodoacetate (MIA) injection modeling for 2 w (OA group); injection of KM13E hydrogel containing 1.5 mg mL^−1^ RIE in OA rats (KM13E group); injection of PGE microspheres containing 2 mg mL^−1^ CIE in OA rats (PGE group); and injection of KM13E@PGE containing 1.5 mg mL^−1^ RIE and 2 mg mL^−1^ CIE in OA rats (KM13E@PGE group). The results reveal a pain‐threshold decrease for all treatment groups by 2 w postoperatively, with the KM13E@PGE group showing the smallest decrease. By 8 w postoperatively, the plantar pain threshold of the KM13E@PGE group had returned to a level similar to that of the control group (Blank), which was significantly different from those of the other groups. Notably, differences were apparent in the plantar pain thresholds obtained for the OA group and the KM13E and KM13E@PGE groups at 4 w; however, there was no significant difference between the OA‐ and PGE‐group results (**Figure** **7**A). Furthermore, we tried to treat with direct injection of CIE at different treatment times, and all groups were injected with RIE on the first day (Figure S15A, Supporting Information); the results showed that all groups had a decrease in plantar pain thresholds at 2 w postoperatively, which was similar to that of the KM13E@PGE group (Figure S15B, Supporting Information). However, at 8 w postoperatively, the plantar pain threshold could not recover to a level similar to that of the Blank group, which was slightly different from that of the KM13E@PGE group. Quantitative analysis of the OA biochemical indicators in the joint cavity indicated considerably higher levels of MMP13, TNF‐α, and IL‐1β for the OA and PGE groups at 2 w relative to those for the KM13E and KM13E@PGE groups. However, by 4 w, the MMP13 levels had decreased significantly with no discernible differences between the treatment groups; in contrast, the TNF‐α and IL‐1β levels exhibited slight upward trends. Furthermore, for the KM13E and KM13E@PGE groups, the IL‐10 levels were consistently elevated at 2 and 4 w postoperatively, with significant differences compared to the other groups (Figure 7B). In contrast, the levels of MMP13, TNF‐α, and IL‐1β in the groups injected directly with RIE and CIE were significantly higher than those in the KM13E@PGE group at 2 w and 4 w postoperatively, and the increase in IL‐10 levels was not as great as in the KM13E@PGE group (Figure S15C, Supporting Information). Direct injection of RIE and CIE did not significantly improve inflammatory factors, but delaying the injection interval of CIE to 14 or 21 d can improve pain to some extent. It can be seen that there is a need for a time interval between anti‐inflammatory treatment and cartilage repair treatment, and the sustained release effect of delivery materials can be more beneficial for the long‐term treatment of OA.

H&E staining revealed numerous cartilage traps with missing chondrocytes on the articular surface at 2 w postoperatively for all groups, except the control group. However, for the KM13E@PGE group at 4 w post‐surgery, a significant number of newly formed chondrocytes appeared in the cartilage cavities. The PGE had the second highest number of these chondrocytes and, thus, more than the KM13E and OA groups (Figure 7C). For the chondrocyte survival‐rate calculation, multiple views were considered. The chondrocyte survival rate was found to be enhanced in the KM13E, PGE, and KM13E@PGE groups at both 2 and 4 w postoperatively, with the KM13E@PGE group having the highest rate (Figure 7D). Safranin O–fast‐green staining demonstrated varying degrees of cartilage ossification at 2 w for all groups, excluding the Blank group, with less ossification observed for the KM13E and KM13E@PGE groups. After 4 w, the KM13E, PGE, and KM13E@PGE groups retained a significant amount of cartilage tissue on their articular surfaces, whereas the OA group exhibited severe cartilage ossification (Figure 7E). From the OA Research Society International (OARSI) score, the KM13E@PGE group exhibited the best recovery, with a reduced score at 4 w (Figure 7F). Immunofluorescence analysis indicated that the OA and PGE groups expressed high levels of TNF‐α protein, whereas the KM13E and KM13E@PGE groups expressed the opposite. Note that TNF‐α protein is an inflammation‐related protein that can serve as a predictor of inflammation severity. Quantitative analysis showed that the inflammation degree at 2 w was considerably lower for the KM13E and KM13E@PGE groups than for the other groups. At 4 w, the KM13E@PGE group exhibited the lowest TNF‐α protein levels among the groups, and the most statistically significant difference (Figure 7G,H). Immunofluorescence imaging of the synovium demonstrated positive expression of CD206 and CD86 proteins (M1 and M2 macrophage markers, respectively) in all groups during OA progression (Figure 7I). Quantitative analysis revealed that, at 2 and 4 w, the KM13E and KM13E@PGE groups had higher proportions of M2 macrophages than the other groups (Figure 7J). Intriguingly, at 4 w, the PGE group showed an improvement in the M2 macrophage proportion, approaching that of the Blank group. However, the difference between these two groups remained statistically significant. These results indicate that the KM13E and KM13E@PGE groups effectively improved the OA‐induced pathological inflammation by promoting M2 macrophage polarization, which is attributed to the RIE. Although the CIE‐carrying PGE had a certain effect on cartilage regeneration, the articular cartilage exhibited a high level of inflammation when PGE alone was employed.

### 2.7. Cartilage Repair Effect of KM13E@PGE In Vivo

**Figure 8:**
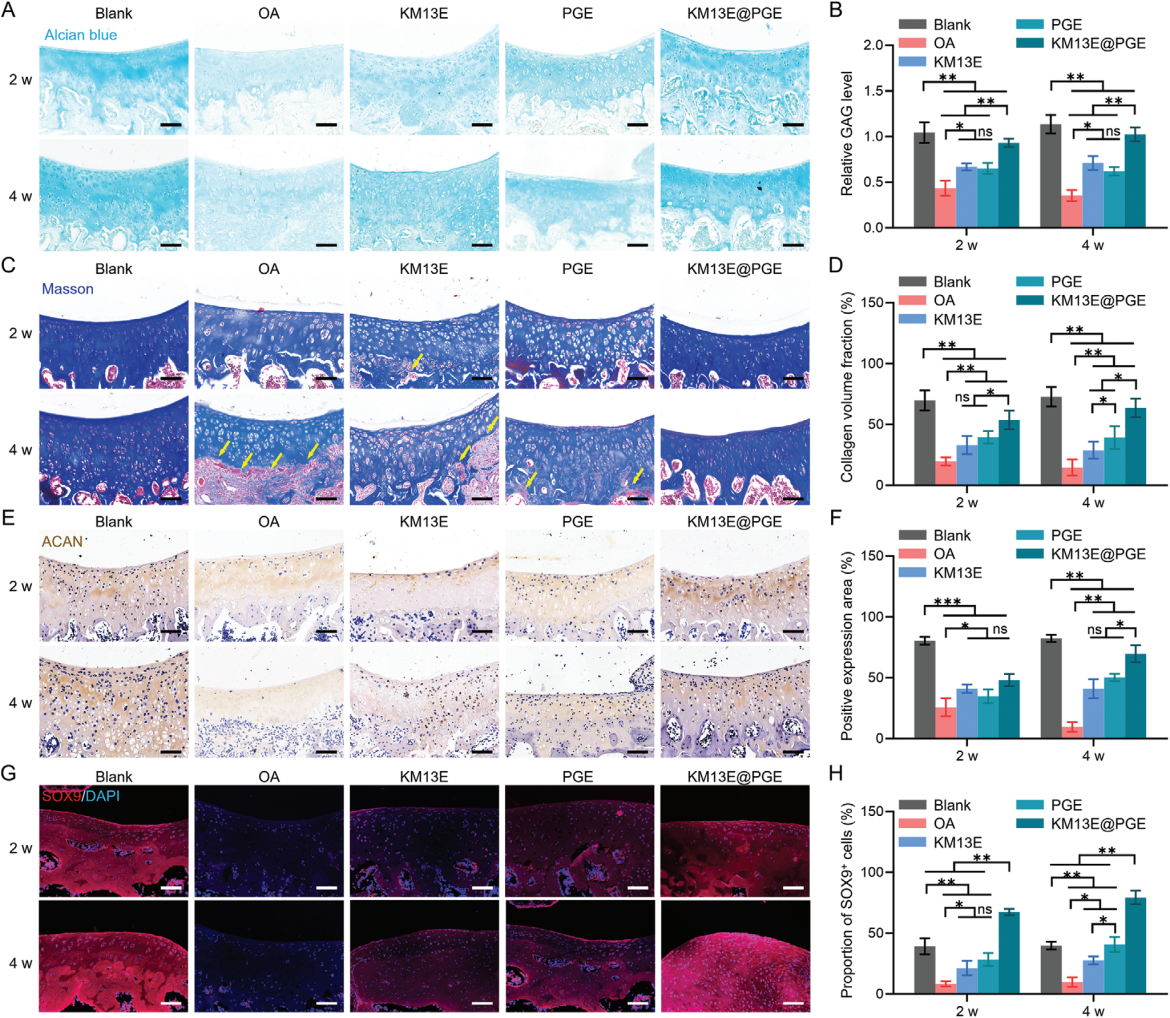
KM13E@PGE promotion of articular cartilage repair in OA rats. A) Alcian blue staining of articular cartilage tissue at 2 and 4 w; scale bar: 200 µm. B) Relative GAG levels are based on Alcian blue staining statistics. C) Masson's staining of articular cartilage tissue at 2 and 4 w; scale bar: 200 µm. D) Collagen volume fractions from Masson's staining. E) ACAN staining of articular cartilage tissue at 2 and 4 w; scale bar; 200 µm. G) Positive areas of ACAN staining. H) SOX9 fluorescent staining of articular cartilage tissue at 2 and 4 w; scale bar: 200 µm. I) SOX9^+^‐cell proportions following SOX9 fluorescent staining. ^**^: *p* < 0.01, ^*^: *p* < 0.05, ns: *p* > 0.05.

Alcian blue staining showed that, at 4 w, all groups exhibited various degrees of cartilage regeneration, with the KM13E@PGE group showing the highest and most significant difference compared with the OA group (**Figure** **8**A). The KM13E@PGE group displayed the highest glycosaminoglycan (GAG) expression levels at both 2 and 4 w, close to those of the Blank group (Figure 8B). Masson's trichrome staining was performed to determine the local‐collagen type and density. At 4 w, all treatment groups, except for the KM13E@PGE group, exhibited varying degrees of type‐I collagen infiltration. Notably, the KM13E@PGE group displayed the highest COL2 density (Figure 8C). The KM13E@PGE group had the highest COL2 expression levels at both 2 and 4 w, which were similar to those of the Blank group (Figure 8D). Immunohistochemical analysis revealed that all groups displayed significant ACAN protein expression, with the KM13E@PGE group exhibiting the highest levels and the most noticeable differences relative to the other groups (Figure 8E,F). Furthermore, immunofluorescence imaging indicated positive *SOX9* protein expression in all groups during the repair phase, suggesting the involvement of chondrocyte differentiation (Figure 8G). Quantitative analysis revealed a significantly higher degree of chondrogenic differentiation for the KM13E@PGE group than the other groups (Figure 8H). Notably, the PGE group displayed the second highest level of differentiation, albeit still lower than that of the Blank group; this level was not significantly different to that of the Blank group at 4 w. These results indicate that the KM13E, PGE, and KM13E@PGE groups promoted cartilage repair, with the latter being the most effective. Therefore, anti‐inflammatory action and tissue regeneration are equally important for OA treatment, and the order of these treatments is even more important (Figure S16, Supporting Information).

## 3. Discussion

Compared to stem cells, EVs can be cryopreserved, have lower immunogenicity, and do not cause tumorigenesis.^[^ ^13^, ^14^ ^]^ EVs also have the ability to regulate target cell functions, showing great potential as biological macromolecule medicines.^[^ ^13^, ^14^ ^]^ Several studies have reported that MSC‐, probiotic‐, and tumor‐derived EVs induce macrophage M2 polarization.^[^ ^29^, ^30^, ^31^, ^32^, ^33^ ^]^ The present study aimed to enrich the M2‐polarizing stimulus, IL‐10, in EVs. Previously, Tang et al.^[^ ^17^ ^]^ used a similar genetic engineering approach to modify the parental cells, thereby achieving IL‐10^+^ EVs that regulated the immune responses during the treatment of ischemic acute kidney injury. In this study, we obtained IL‐10^+^ EVs (RIE) without dexamethasone, which reduced the time for engineering treatment of RIE and removed the potential effects of dexamethasone, but focused more on the effects of IL‐10. We also found that RIE is rich in CCL18, which promotes the M2 polarization of macrophages through the PI3K‐Akt pathway.^[^ ^34^ ^]^ This pathway is critical for the ATP/adenosine diphosphate (ADP) response and may contribute to the promotion of aerobic glucose metabolism in macrophages, thereby facilitating their polarization toward the M2 phenotype.^[^ ^35^ ^]^ Upon detection of several integrins, we found that the α4 integrin expression in the RIE was enhanced after the IL‐10 overexpression. The α4 integrin may interact with the β1 integrin expressed by the macrophages, thus promoting targeted action.^[^ ^36^ ^]^ However, the RIE expression of αmβ2, which is related to phagocytosis, did not change significantly.^[^ ^37^ ^]^ This indicates that the RIE is more prone to macrophage internalization but the possibility of phagocytosis and degradation exists. This is important because macrophages are the immune cells that respond most rapidly to exogenous EVs in the innate immune system,^[^ ^38^ ^]^ and also key for us to ensure that RIE takes effect in the joint cavity. Note that we screened the in vitro effective RIE concentration and the KM13E release rate to ensure accurate dosing and high RIE utilization.

The IPFP tissue plays a role in promoting inflammation and angiogenesis during the development of OA.^[^ ^39^, ^40^ ^]^ However, the EVs secreted by IPFP‐MSCs possess potential therapeutic effects.^[^ ^41^, ^42^ ^]^ These dual effects prompted us to adopt an engineered strategy involving chondrogenic induction cultures to induce EVs. Wu et al.^[^ ^20^ ^]^ found that EVs derived from IPFP‐MSCs are rich in miR‐100‐5p, which can downregulate the mammalian target of rapamycin (mTOR) pathway to protect articular cartilage. These EVs also inhibit fibroblast proliferation by suppressing metallothionein‐2A in an inflammatory environment. In this study, we found that FOXO1, GDF5, and SOX9 were enriched in the CIE through proteomics. In chondrocytes, CIE promotes COL2 and ACAN secretion, which improves the chondrosphere cohesion. This process is caused by exogenous SOX9 delivery from the CIE and, also, because CIE delivers FOXO1 to chondrocytes exogenously, specifically binds to FOXO3A, and promotes the transcription of endogenous *SOX9*. This process suppresses fatty acid oxidation and induces chondrogenic commitment to a low‐fat environment.^[^ ^43^ ^]^ Considering the tendency of IPFP‐MSCs to undergo adipogenic differentiation, this mechanism further confirms the necessity for chondrogenic differentiation of IPFP‐MSCs. As for the RIE, the effects of different CIE concentrations were screened in vitro in this study. As to whether CIE has a targeting effect on chondrocytes, we have not been able to study this in‐depth, but based on the homologous targeting theory,^[^ ^44^ ^]^ this possibility exists.

At the onset of OA, inflammatory factors such as IL‐1β, IL‐7, and TNF‐α stimulate proteinase production, including MMPs and a disintegrin and metalloproteinase with thrombospondin motifs (ADAMTS).^[^ ^27^, ^45^ ^]^ MMP‐1, −2, −3, −9, and −13 are expressed in the OA pathological environment.^[^ ^27^ ^]^ MMP13 degrades proteoglycans, inducing a dual degradation effect on the cartilage matrix.^[^ ^46^ ^]^ Materials that stimulus‐responsed to MMPs biomarker have attracted attention, such as MMP2‐sensitive drug delivery materials used in renal ischemia/reperfusion injury.^[^ ^47^ ^]^ In our previous study, we presented an MMP1 enzyme‐responsive hydrogel that responds to angiogenesis during bone repair.^[^ ^28^ ^]^ Previously, Kisiday et al.^[^ ^48^ ^]^ compared the KLDL‐12 SAP system with the RADA‐16 SAP system and revealed effective mimicry of the natural ECM by the former, which facilitated cartilage repair. Building upon this finding, we focused on developing a KM13 hydrogel, which is an MMP13 degradable gel based on a similar KLDL‐12 SAP system. We integrated the MMP13 substrate sequence proposed by Hubbell et al.^[^ ^49^ ^]^ into this system, thereby creating a KM13 short peptide sequence. Our KM13 hydrogel demonstrated the capacity for spontaneous gelation within a rapid 5 min timeframe. Moreover, its storage and loss moduli and COF closely resembled those of sodium hyaluronate, a commercially available injectable drug widely used in joint cavity treatment. Through in vivo and in vitro experiments, we confirmed the ability of this material to serve as the outermost release structure for programmed‐release materials, enabling EV release in accordance with the degree of inflammation in the OA joint cavity.

The differential release rate realized by the integrated delivery of the two EV types has not been achieved in previous studies. To sustainably release the CIE after the initial RIE burst release, microspheres were incorporated as physical structural systems into the stimuli‐responsive system. We observed differential release rates of fluorescence and biochemical markers both in vitro and in vivo. This “microspheres‐in‐gel” system enables step‐by‐step treatment, which is not only recommended in clinical therapy, but has also been explored by Mitra et al.^[^ ^50^ ^]^ and Leung et al.^[^ ^51^ ^]^ Although the direct construction of bilayer‐structured microspheres can achieve step‐by‐step release to some extent, in this study, we did not use an MMP13‐sensitive sequence‐carrying SAP to prepare microspheres via a microfluidic chip. This is because an SAP with an MMP13‐sensitive sequence is electroneutral, which causes faster gel formation. Thus, we previously investigated the “microspheres‐in‐gel” system.^[^ ^52^ ^]^ Note that the GelMA component of PGE may serve as a substrate for collagenase MMP13; therefore, PGE exhibits a less‐intense sudden release in an MMP13 solution. However, KM13E@PGE extended the CIE release cycle in this study, especially in the in vivo experiments, which confirmed the role of the “microspheres‐in‐gel” physical structure in prolonging the CIE release. Further, the microspheres in the KM13E@PGE composite system are smaller than the diameter of a syringe needle, and the outer layer of KM13 has shear‐thinning properties, so the whole system can be injected. Based on the fact that KLDL‐12 has self‐healing properties,^[^ ^53^ ^]^ we speculate that the KM13 has a slow self‐healing property after injection, so the mechanical properties and structural arrangements of the hydrogel network will not be particularly affected, and the release characteristics of the EVs will not be altered.

The M2 macrophages showed increased phagocytic activity upon RIE regulation, potentially hindering EV retention. Therefore, CIE needs to be released later on. Controlled drug release using stimuli‐responsive materials is essential for targeted and timed delivery, especially with the advent of multidrug protocols.^[^ ^54^, ^55^ ^]^ Zhang et al.^[^ ^56^ ^]^ introduced an innovative and highly programmable platform for sequential drug release in tissue engineering, which enables the controlled release of more than three proteins in a specific sequence. The present study constitutes significant progress, this is not only achieved by the MMP13 stimuli‐responsive of KM13E@PGE, but also the “microspheres‐in‐gel” structure ensures that the two types of EVs have different release rates. In addition, EVs exert different therapeutic effects on macrophages and chondrocytes, constituting a “temporal AND spatial” logic‐gate function, collectively achieving spatiotemporally controlled release. This approach distinguishes the proposed method from the current practice of simply mixing multiple EVs during treatment, as demonstrated by Wu et al.^[^ ^57^ ^]^

To establish an OA model, we employed the joint cavity injection method using MIA, which caused significant mobility impairment, cartilage degradation, and the development of an inflammatory environment within the joint cavities of rats.^[^ ^58^ ^]^ Compared with surgical excision of the anterior cruciate ligament, our MIA‐induced OA model yielded a more pronounced alteration in the inflammatory environment within the joint cavity. We used a method of injecting RIE and CIE at different intervals to simulate sequential anti‐inflammatory and cartilage repair treatments. We found that the overlap of the two treatment times was not conducive to the improvement of validation indicators and pain. When the injection interval between RIE and CIE was more than 2 weeks, the pain improved, but due to the lack of a sustained‐release system for EVs, the therapeutic effect was not satisfactory. KM13E, which was developed by the authors, exhibited significant anti‐inflammatory properties by enhancing the M2 polarization of the macrophages and reducing the inflammatory‐factor levels. However, despite the lack of pro‐chondrogenic differentiation factors, the effectiveness of KM13E for cartilage repair is limited, which can be attributed to the repair effects of the M2 macrophages and KLDL‐12 on tissue. The persistent inflammatory microenvironment further deteriorates the cartilage ECM, contributing to OA progression.^[^ ^59^ ^]^ Conversely, KM13E@PGE exhibits a comprehensive therapeutic effect, as evidenced by its OARSI and synovial inflammation scores, which were the lowest, as well as a notable increase in the cartilage repair indicators, including *ACAN* and *SOX9*. Additionally, although the in vitro and in vivo experiments revealed that KM13E@PGE releases RIE almost completely within 7 d in response to MMP13, the TNF‐α level at 4 w was lower than at 2 w, which was not obvious for the KM13E group. We believe a feedback regulation mechanism exists; that is, the reduction in inflammatory factors synthesized by the CIE‐targeted chondrocytes consolidates the low‐inflammatory microenvironment. Several studies have investigated the potential of EVs in terms of their anti‐inflammatory and cartilage‐repair properties. Wei et al.^[^ ^60^ ^]^ examined the use of MSC‐derived exosomes to alleviate temporomandibular joint OA by reducing inflammation. Similarly, Lin et al.^[^ ^61^ ^]^ developed MSC‐derived exosomes that promoted chondrocyte proliferation via the lncRNA‐KLF3‐AS1/miR‐206/GIT1 axis. However, none of these studies comprehensively addressed the dual requirements of anti‐inflammatory and reparative strategies in clinical OA therapy.

Based on the excellent OA efficacy of KGM13E@PGE, we have further demonstrated that the “anti‐inflammatory first AND cartilage repair second” logic‐gate treatment strategy is necessary. Currently, achieving both anti‐inflammatory and reparative effects using EVs remains a challenge, which necessitates the development of drug delivery materials with an affinity for EVs and an intelligent response for controlled release.^[^ ^62^ ^]^ In addition, these materials must be injectable and minimally invasive in certain cases. Compared to clinical treatment methods for OA, we believe that a single intra‐articular injection of KM13E@PGE can be expected to have a sustained‐release effect lasting up to one month, potentially replacing a course of treatment with four injections of sodium hyaluronate. This work has used MMP13 concentration which is consistent with the animal model. This concentration may vary from that of patients. We believe that by adjusting the concentration of KM13, we can meet the release rate required for clinical treatment. Our KM13E@PGE delivery system not only effectively facilitates therapeutic EV application, but also serves as a potential mediator for integrated step‐by‐step therapies involving drug and stem cell delivery. As a flexible and spatiotemporally controlled delivery system for multiple EVs, with minor modifications, this approach can also serve as an effective therapeutic tool for diseases requiring combination drug scenarios, such as novel coronavirus pneumonia. In this work, we focused on the effects of EVs on macrophages and chondrocytes, however, other types of immune cells and tissue cells could not be detected in their entirety, and we believe that the wide range of regulatory effects of EVs may have a more diverse impact on OA treatment, which will be gradually refined in our subsequent work.

## 4. Conclusion

In this study, we have developed a logic‐based system involving two engineered EVs for intra‐articular injection for OA treatment. Early release of IL‐10^+^ EVs, which acted as an anti‐inflammatory agent, was achieved, followed by later release of SOX9^+^ EVs, which had cartilage‐repair effects. This integrated in vivo delivery effectively enabled step‐by‐step therapy of OA. To achieve differential release rates of the two EV types in the joint cavity, an MMP13‐sensitive hydrogel and related “microspheres‐in‐gel” structure were used for spatiotemporally controlled EVs release. This work may inspire multi‐targeted, integrated applications of multiple EVs, providing informative approaches to differential rate release and enzyme‐sensitive controlled release.

## Conflict of Interest

The authors declare no conflict of interest.

## Supporting information

Supporting Information

## Data Availability

The data that support the findings of this study are available from the corresponding author upon reasonable request.
