# Supplementary material for: Logic‐Based Strategy for Spatiotemporal Release of Dual Extracellular Vesicles in Osteoarthritis Treatment
Source: Adv Sci (Weinh). 2024 May 5;11(26):2403227. doi: 10.1002/advs.202403227 (PMC11234466; doi:10.1002/advs.202403227)
Supplement: Supplementary file 1 — Supporting Information [file ADVS-11-2403227-s001.pdf]

ADVANCED SCIENCE
Open Access

Supporting Information

for *Adv. Sci.*, DOI 10.1002/advs.202403227

Logic-Based Strategy for Spatiotemporal Release of Dual Extracellular Vesicles in Osteoarthritis Treatment

Shiyu Li, Weihan Zheng, Wenfeng Deng, Ziyue Li, Jiaxin Yang, Huihui Zhang, Zhenning Dai, Weiwei Su, Zi Yan, Wanting Xue, Xinyi Yun, Siqi Mi, Jianlin Shen, Xiang Luo, Ling Wang, Yaobin Wu* and Wenhua Huang**

Supporting Information

Logic-based Strategy for Spatiotemporal Release of Dual Extracellular Vesicles in Osteoarthritis Treatment

Shiyu Li †, Weihan Zheng†, Wenfeng Deng†, Ziyue Li, Jiaxin Yang, Huihui Zhang, Zhenning Dai, Weiwei Su, Zi Yan, Wanting Xue, Xinyi Yun, Siqi Mi, Jianlin Shen, Xiang Luo, Ling Wang*, Yaobin Wu*, Wenhua Huang*.

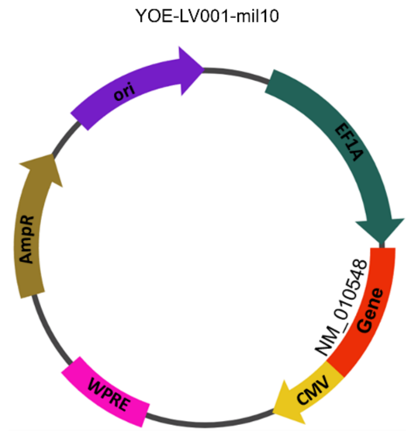

Figure S1. Schematic diagram of IL-10 overexpression plasmid.

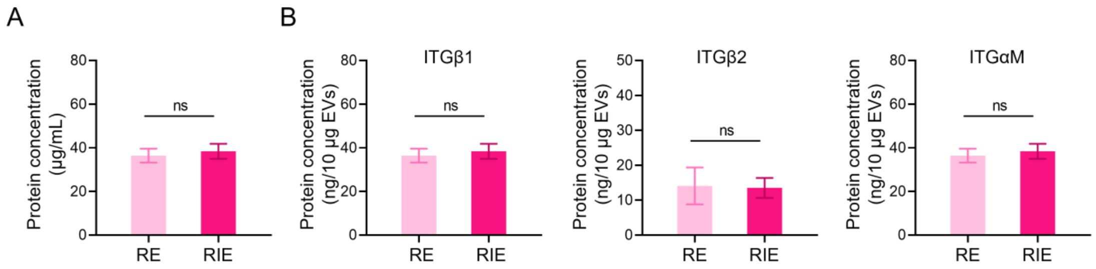

Figure S2. Comparison of separation concentration and integrin content between RE and RIE. (A) Protein quantification assay of RE and RIE using the BCA kit. (B) Detection of ITGβ1, ITGβ2, ITGαM in RE and RIE by ELISA. ns: $p > 0.05$.

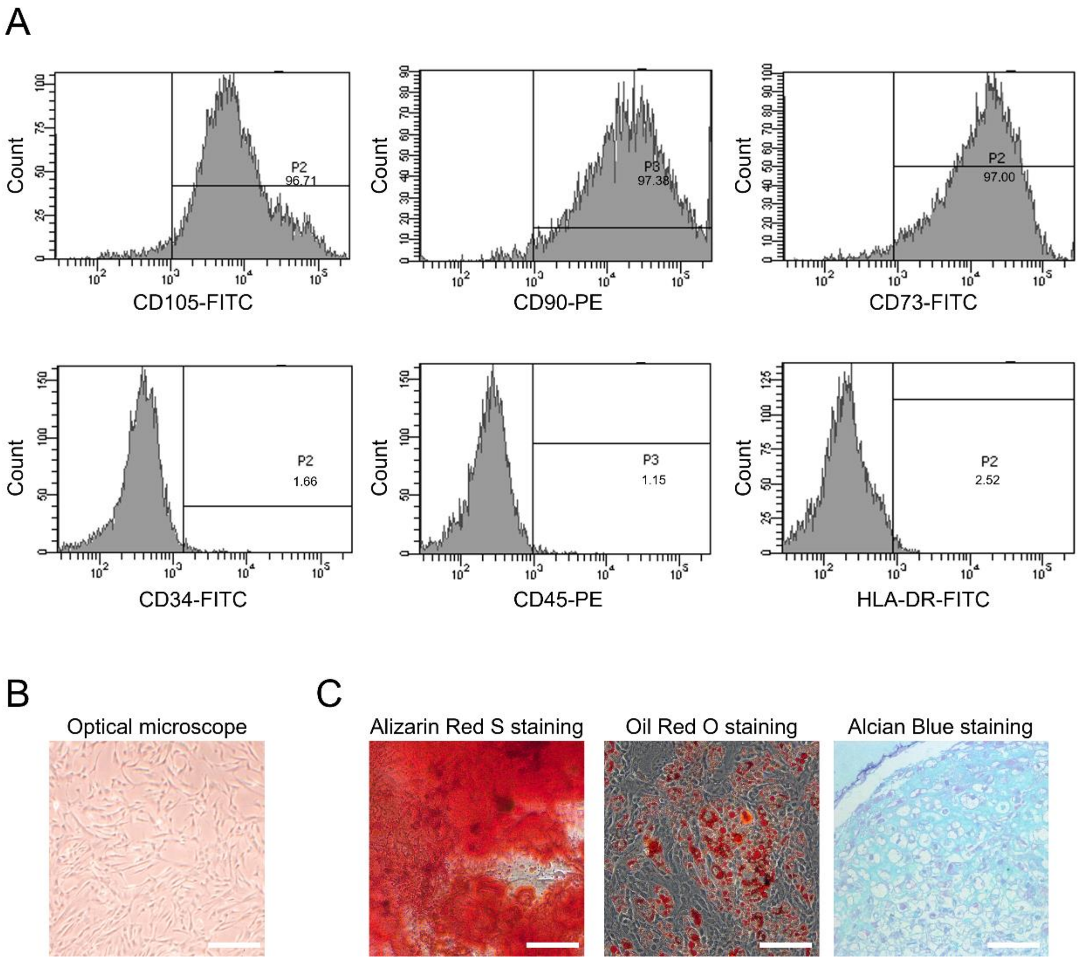

Figure S3. Identification of IPFP-MSCs. (A) Identification of surface markers by flow cytometry. (B) Cell adhesion capacity and morphology by light microscopy (C) Validation of trilineage differentiation potential: Alizarin Red S staining, induced for 21 days; Oil Red O staining, induced for 14 days; Alcian Blue staining, induced for 21 days. Scale bar: 50 μm.

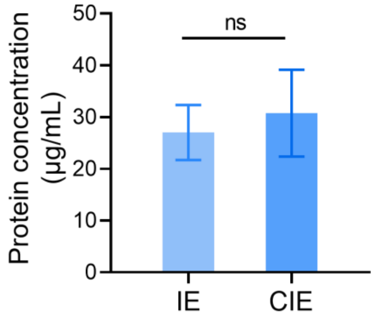

Figure S4. Protein quantification assay of IE and CIE using the BCA kit. ns: $p > 0.05$.

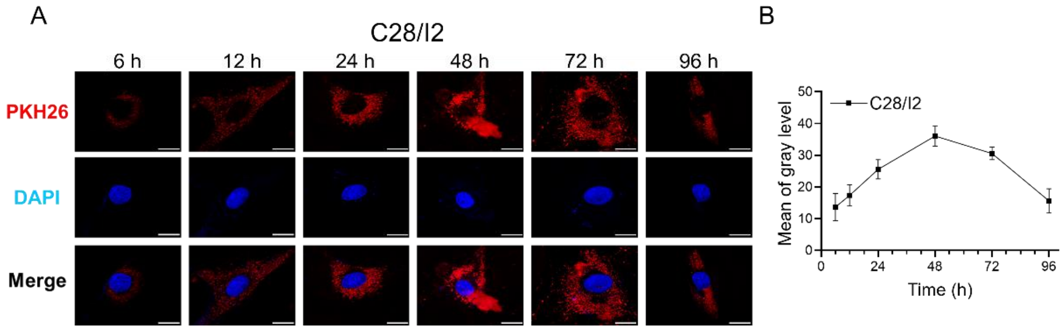

Figure S5. Internalization of CIE uptake by C28/I2 cells. (A, B) Immunofluorescence micrograph and fluorescence intensity quantitative analysis of PKH-26 labeled CIE uptake by C28/I2 at 6, 12, 24, 48, 72, and 96 h. Scale bar: 20 μm.

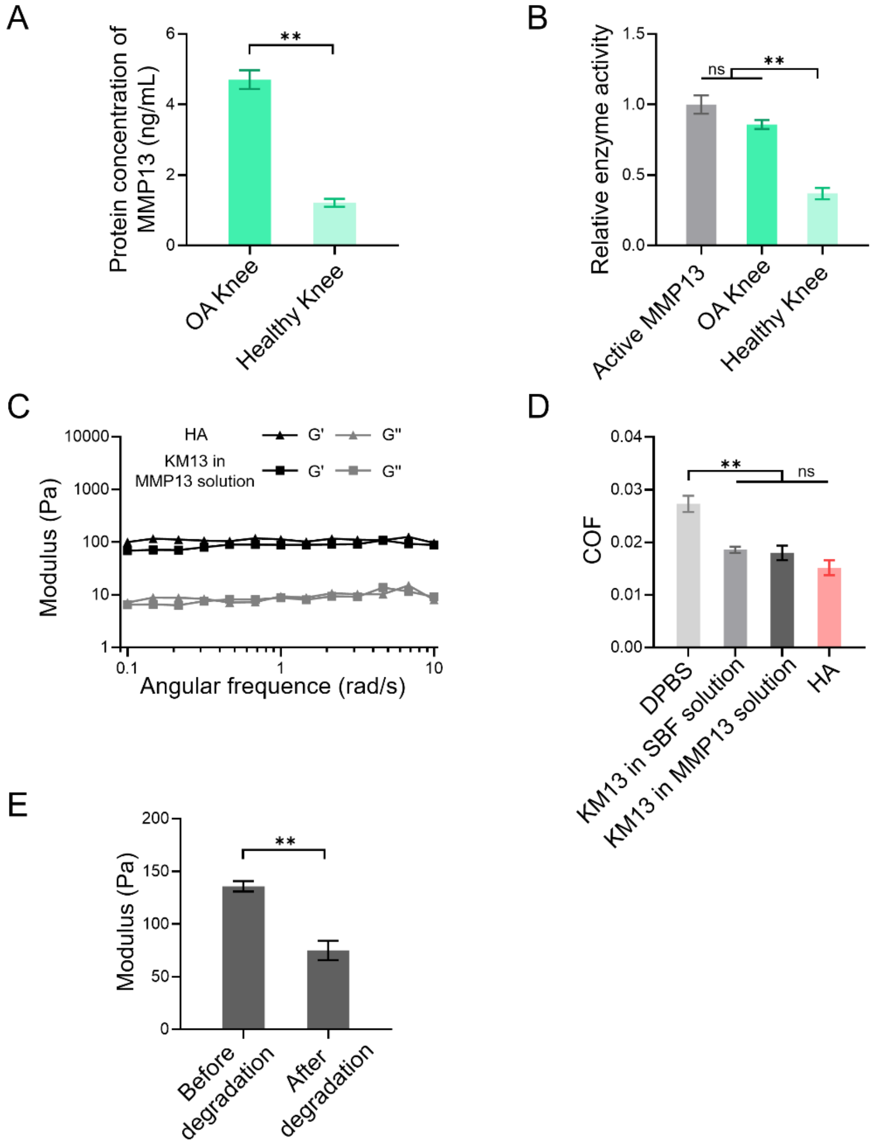

Figure S6. The content of MMP13 in joint fluid, and the modulus of KM13 after digestion by MMP13. (A) Relative enzyme activity assay of active enzyme, joint fluid of OA rats (OA knee) and joint fluid of wild-type rats (healthy knee). OA rats were modeled after 2 w. (B) Quantification of MMP13 protein concentration in OA knee and Healthy knee by ELISA. (C) Storage modulus (G') and loss modulus (G'') of KM13 in MMP13 solution (SBF containing 5 ng/mL MMP13) and commercial HA in SBF solution, in the angular frequency ranging from 0.1- 10 rad/s (1% strain). (D) COF of DPBS (1×), 1% (w/v) KM13 in SBF solution, KM13 in MMP13 solution and HA in SBF solution. (E) Shear modulus of KM13 before and after degradation. **: $p < 0.01$.

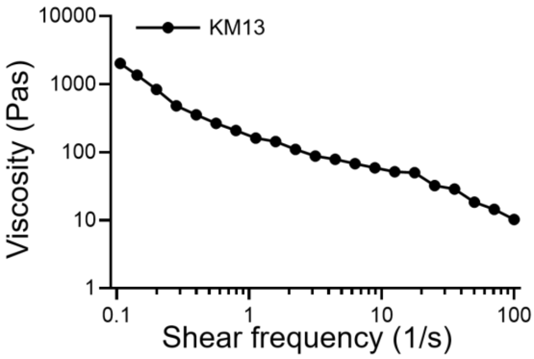

Figure S7. Shear thinning behaviour of KM13.

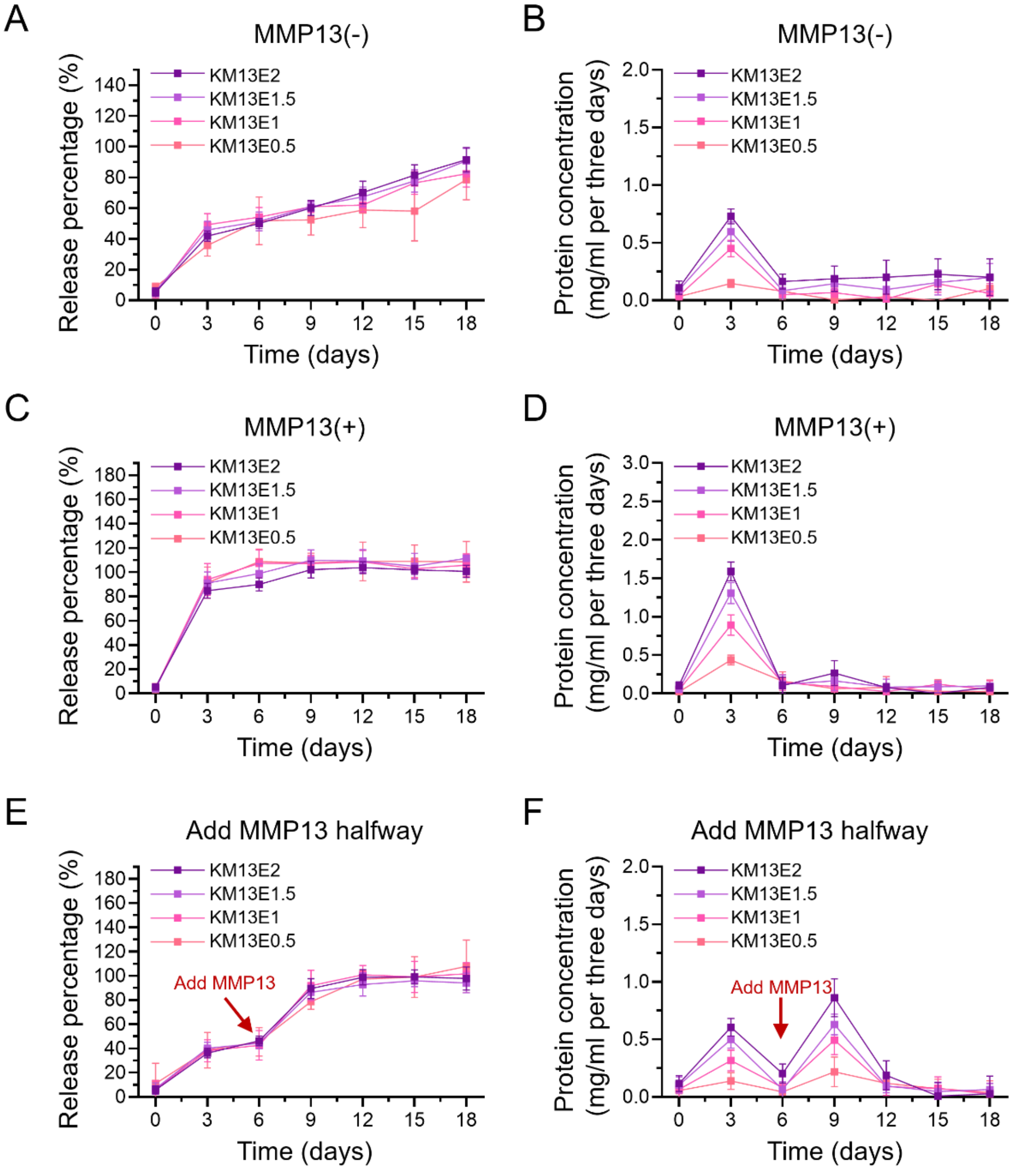

Figure S8. Controlled release of KM13E in SBF or MMP13 solution. (A-B) BCA assay of KM13E

in SBF solution over 18 d. (A) Percentage of released protein. (B) Concentration of protein released every 3 days. (C-D) BCA assay of KM13E in MMP13 solution (SBF containing 5 ng/mL MMP13) over 18 d. (C) Percentage of released protein. (D) Concentration of protein released every 3 days. (E-F) BCA assay of KM13E in SBF solution (add MMP13 with a final concentration of 5ng/mL at 6d) over 18 d. (E) Percentage of released protein. (F) Concentration of protein released every 3 days.

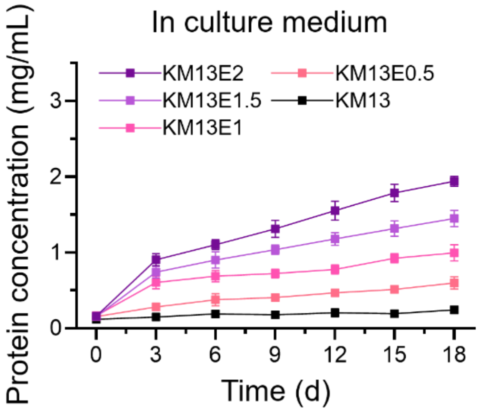

Figure S9. BCA assay of KM13E containing different concentrations of RIE (0, 0.5, 1, 1.5, and 2 mg/mL) for 18 d in the medium.

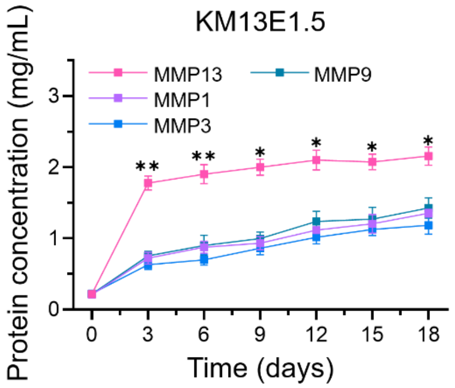

Figure S10. BCA assay of KM13E1.5 in different kinds of MMPs solutions (MMP1, MMP3, MMP9, MMP13 all with final concentration of 5 ng/mL) over 18 d. **: $p < 0.01$, *: $p < 0.05$.

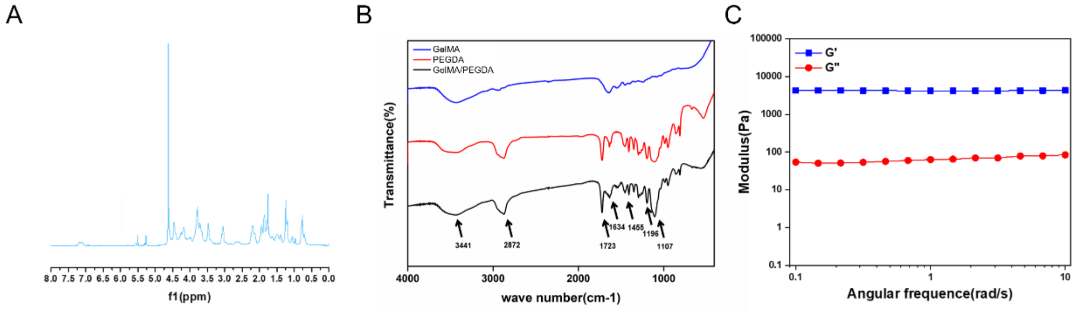

Figure S11. Characterisation of PG (PEGDA /GelMA) hydrogels. (A)^{1}HNMR detection of GelMA. (B) FT-IR spectroscopy analysis of GelMA, PEGDA, and PG. (C) Storage modulus (G') and loss modulus (G'') of the PG hydrogel in the angular frequency ranging from 0.1-10 rad/s (1% strain).

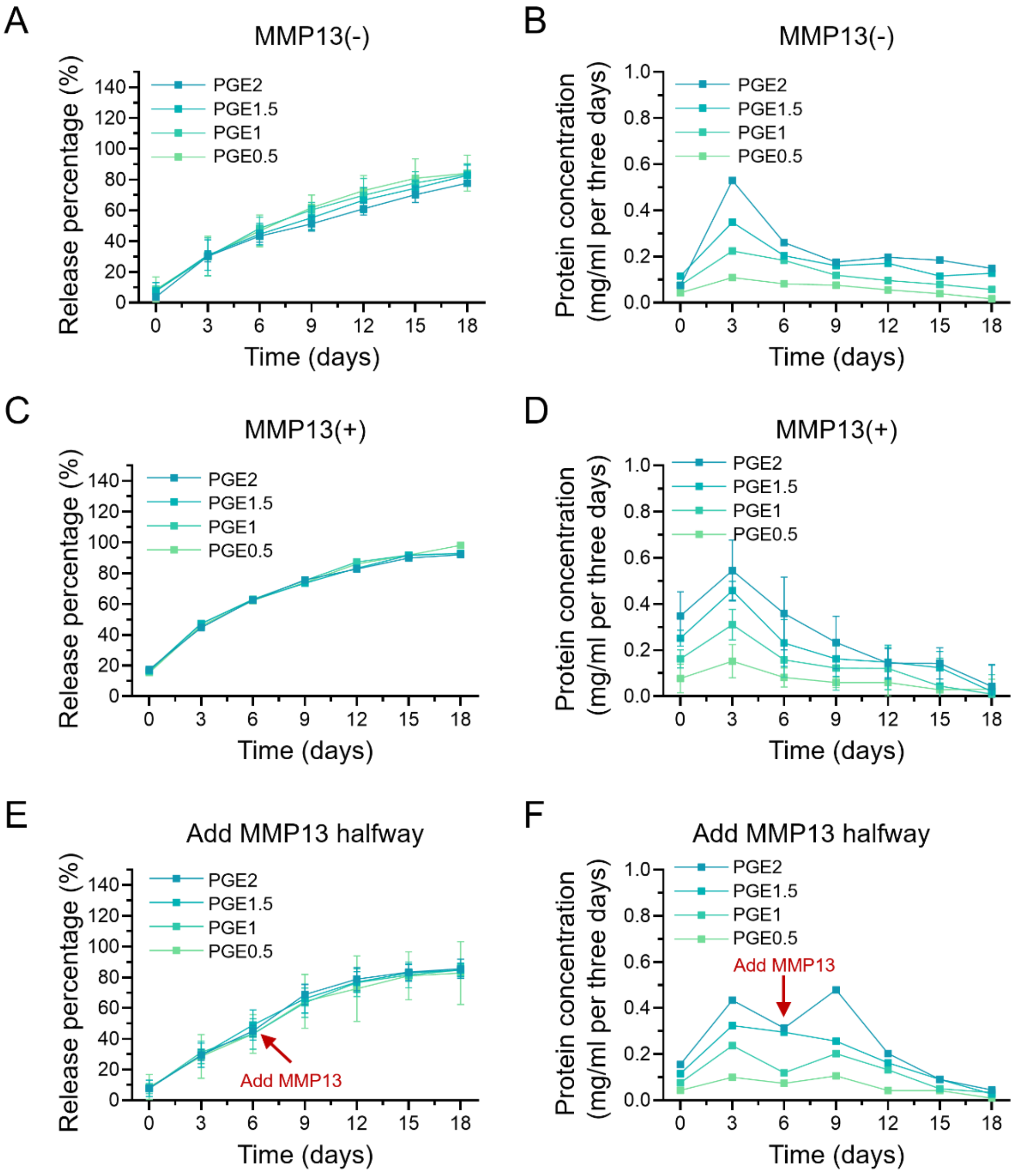

Figure S12. Controlled release of PGE microspheres in SBF or MMP13 solutions. (A-B) BCA assay of PGE microspheres in SBF solution over 18 d. (A) Percentage of released protein. (B) Concentration of protein released every 3 days. (C-D) BCA assay of PGE microspheres in MMP13 solution (SBF containing 5 ng/mL MMP13) over 18 d. (C) Percentage of released protein. (D) Concentration of protein released every 3 days. (E-F) BCA assay of PGE microspheres in SBF solution (add MMP13 with a final concentration of 5 ng/mL at 6d) over 18 d.

(E) Percentage of released protein. (F) Concentration of protein released every 3 days.

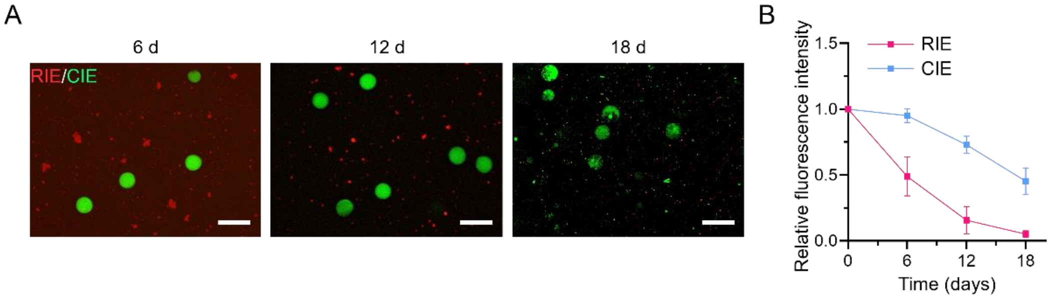

Figure S13. *In vitro* release of EVs from KM13E@PGE hydrogel at 6, 12, 18 d in MMP13 solution. (A) Immunofluorescence images, scale bar: 100 μm. (B) Fluorescence intensity quantitative analysis.

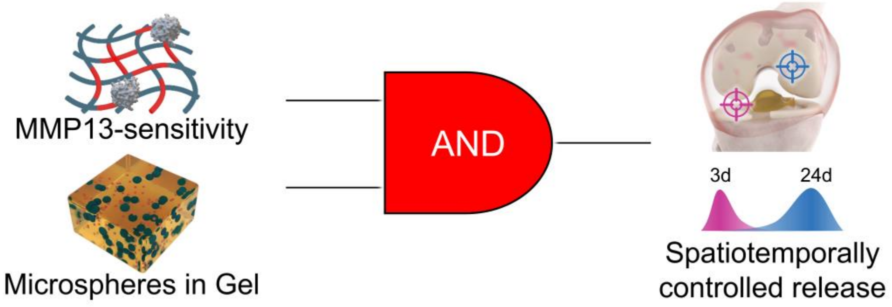

Input		Output
MMP13-sensitivity	"Microspheres in Gel" structure	Spatiotemporally controlled release
1	1	1
1	0	0
0	1	0
0	0	0

Figure S14. Schematic illustration of AND logic-gate: Spatiotemporally controlled release = MMP13-sensitivity · Microsphere-in-Gel.

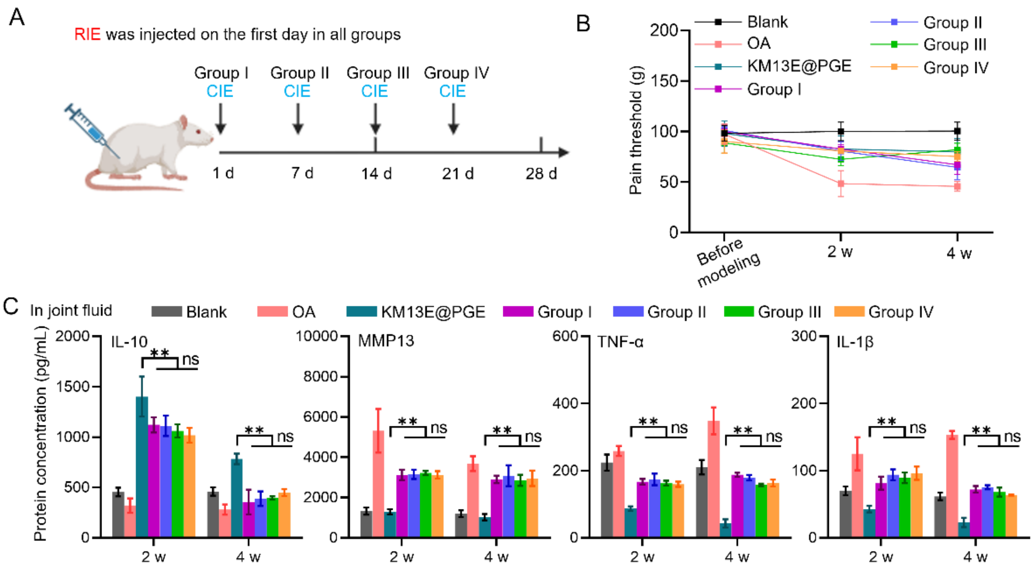

Figure S15. Improvement of the inflammatory microenvironment in the joint cavity of OA rats by direct injection of RIE and CIE. Groups: Blank: saline injection without modeling; OA: MIA injection modeling for 2 w; KM13E@PGE: KM13E@PGE (containing 1.5 mg/mL RIE and 2 mg/mL CIE) injection; Group I: RIE and CIE both injected on 1 d; Group II: RIE injected on 1 d, CIE injected on 7 d; Group III: RIE injected on 1 d, CIE injected on 14 d; Group IV: RIE injected on 1 d, CIE injected on 21 d; (A) Treatment grouping schematic. (A) Plantar pain threshold analysis results, before modeling, 2 and 4 w after modeling. (B) ELISA detection of IL-10, MMP13, TNF-α, and IL-1β concentrations in joint fluid. **: $p < 0.01$, ns: $p > 0.05$.

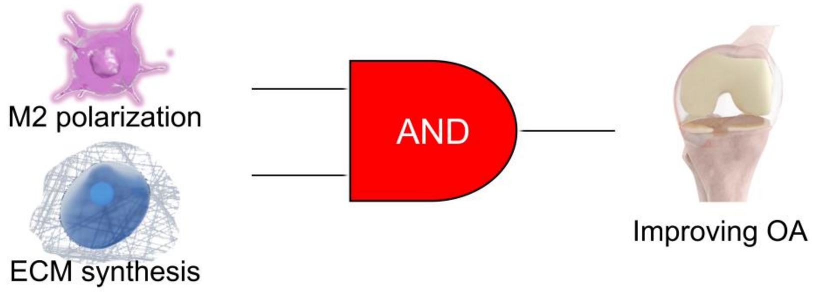

Input		Output
Anti-inflammatory first	Cartilage repair second	Improving OA
1	1	1
1	0	0
0	1	0
0	0	0

Figure S16. Schematic illustration of AND logic-gate: Improving OA = Anti-inflammatory first · Cartilage repair second.

Experimental Section

Ethical statement

Samples of healthy human infrapatellar fat pad tissue were collected from the Third Affiliated Hospital of Southern Medical University. The collection was approved by the ethics committee, and all healthy donors provided written informed consent.

The animal experiments referred to in this study were conducted with the approval of the ethics committee of the Third Affiliated Hospital of Southern Medical University and in strict accordance with the ARRIVE 2.0 guidelines and the "Guidelines for Ethics Review of Laboratory Animals' Welfare" (GB/T 35892-2018, China).

Cell culture & Grouping

RAW264.7 and C28/I2 cell lines were purchased from the American Type Culture Collection (ATCC, US). IPFP-MSCs were obtained according to a previously described enzymatic digestion method[1]. The RAW264.7 and C28/I2 cells were cultured in Dulbecco's modified Eagle's medium (DMEM, C11995500BT, Gibco, US) containing 10% EV-free foetal bovine serum (EXO-FBS-50A,

SBI, US) and 1% penicillin-streptomycin (PS, 10000 U/mL, 15140122, Gibco, US). The IPFP-MSCs were cultured in Dulbecco's modified Eagle's medium F-12 (DMEM/F-12, C11330500BT, Gibco, US), containing 10% EV-free FBS and 1% PS. All cells were maintained under controlled culture conditions at a constant temperature of 37 °C and a CO_2 concentration of 5% in a humidified incubator.

For the IPFP-MSCs induced by the proliferation medium (IPFP-MSC) group, the cells were not treated. For the IPFP-MSCs induced by the chondrogenic medium (C-IPFP-MSC) group, the cells were cultured in the chondrogenic differentiation medium (containing TGF-β1, dexamethasone, ascorbic acid, and ITS+1, HUXXC-90041, Cyagen, US) for 2 weeks following the manufacturer's instructions. To fulfill the MSC characterization requirements, the cells were also induced using an osteogenic induction medium (HUXXC-90021, Cyagen, US) and adipogenic induction medium (HUXMD-90031, Cyagen, US). Flow cytometry was utilized to identify the relevant surface proteins CD105, CD90, CD73, CD34, CD45, and HLA-DR[2], and the antibody information can be found in Table S3.

For the overexpressed IL-10 in the RAW264.7 (IL-10^+ RAW) group, cells were infected with the YOE-LV001-mIL10 lentivirus (Ubigene, China), as in a previous study[3]. Following plasmid transfection of *Escherichia coli*, the positive bacteria were screened by culturing on agarose plates supplemented with ampicillin. The CDS-seq of the target gene, NM_010548, is available at https://www.ncbi.nlm.nih.gov/nuccore/NM_010548.2. For the RAW264.7 induced with the proliferation medium (WT-RAW), cells were collected for subsequent RT-qPCR and western blotting. The primer and antibody information is shown in Tables S2 and S3.

EVs Isolation and Identification

Isolation of EVs from cell supernatants. EVs from the IL-10^+ RAW (RIE), WT-RAW (RE), IPFP-MSC (IE), and C-IPFP-MSC (CIE) groups were extracted according to a previously described multi-step centrifugation protocol[4]. Cells from all groups were cultured in T75 flasks (Corning, NY, US) until they reached 80% confluency. After 72 hours of culture, the conditioned medium was gathered and subjected to sequential centrifugation steps: 300 *g* for 10 min, 2,000 *g* for 10 min, and 10,000 *g* for 30 min, all performed at a temperature of 4 °C to eliminate dead cells and cell debris. The resultant supernatant was then ultracentrifuged at 100,000 *g* for 70 min at 4 °C, and the sediment

was washed with DPBS (C14190500BT, Gibco, US) before being ultracentrifuged again at 100,000 *g* for 70 min at 4 °C. Finally, the pellet was resuspended in particle-free PBS, and its concentration was determined using a BCA kit (23225, Thermo Scientific, US), following the manufacturer's instructions. All groups of EVs were stored at -80 °C for subsequent characterization experiments.

Western blot. Following the recommendations of Minimal Information for Studies on Extracellular Vesicles 2018 (MISEV2018)[5], western blotting was used to detect the positive markers CD9, CD81, and ALIX for EVs and the negative marker Calnexin. The cell lysates were used as controls. The antibody information is shown in Table S3.

NTA. NTA was conducted using ZetaView PMX 110 (Particle Metrix) and the corresponding software (ZetaView v8.02.28). The EVs were diluted in particle-free PBS and loaded into the sample cell chamber prior to size distribution and concentration determination at a wavelength of 405 nm.

TEM. The morphology of EVs was analyzed using TEM. The collected EVs were carefully added in small increments to a 200-mesh copper grid for negative staining with 2% tungstate phosphate (pH 7.0, 1 min). Subsequently, images were captured at 80 kV using TEM (H-7650, Hitachi; JEM-1400, Jeol).

Protein Profiling & Bioinformatics Analysis

To compare protein profiles, the RE and RIE groups were used. Another set of comparisons was performed on the IE and RIE groups. Samples were lysed using lysis buffer (1% sodiumdeoxycholate, 1% protease inhibitor) at a 4-fold volume and then incubated overnight at 56 °C. Ultrasonication was performed to lyse the samples, followed by centrifugation at 12,000 *g* for 10 min to remove cell debris. The supernatant was transferred to a new tube and reduced with DL-dithiothreitol (5 mM) at 56 °C for 30 min. Next, iodoacetamide (11 mM) was added, and the cells were incubated for 15 min at room temperature in the dark. Tetraethylammonium bromide was added to dilute the urea concentration below 2 M. Trypsin was added at a protease-to-protein ratio of 1:50 (m/m) and incubated overnight, followed by another addition of trypsin at a ratio of 1:100 (m/m) the following day. Digestion was continued for 4 h to obtain samples for mass spectrometry analysis. The peptide samples were loaded onto a nanoflow LC (NLC-MS/MS) system connected to a mass spectrometer (Orbitrap Exploris 480, Thermo Scientific, US) through a nanoelectrospray ionization source. The analysis was repeated twice for each sample. High-resolution Orbitrap was

used to detect and analyze both peptide precursor ions and their secondary fragments, with secondary spectral data used to determine the distribution of proteins between groups.

Functional enrichment analyses were conducted on the DEPs to determine their biological functions. The gene ontology (GO) system, which includes three ontologies (molecular function, cellular components, and biological processes), was used for functional gene classification. Genes associated with the detected peaks were mapped to specific GO terms in the gene ontology database (GO.db 3.8.2). Gene numbers were calculated for each term, and hypergeometric tests were conducted to identify significantly enriched GO terms among the differentially expressed genes compared to the background genome. Pathway-based analysis was used to gain further insight into the biological functions of these genes. The Kyoto Encyclopedia of Genes and Genomes (KEGG), a comprehensive public pathway database (Release 94), was used for the analysis.

Content identification and functional validation of EVs

ELISA. To quantify the concentration of specific proteins in the EVs, ELISA was performed using standard procedures. The ELISA kit information is shown in Table S4.

Immunohistochemistry. To validate the functionality of the CIE in C28/I2 cells, chondrogenic differentiation was performed. The positive group was induced using the chondrogenic differentiation medium, whereas the control group was induced using the proliferation medium. The concentration of EVs in all EV groups was maintained at 300 μg/mL, and the specific experimental steps have been described above. Following induction, all the groups were fixed, embedded in paraffin, and sectioned. The sections were exposed to COL2, ACAN, and SOX9 antibodies, respectively, and incubated at 4 °C for 10 h. Subsequently, goat anti-rabbit IgG H&L (HRP) was added and incubated for 1 h at room temperature. Immunohistochemical staining was performed using the 3,3'-diaminobenzidine tetrahydrochloride kit (P0202, Beyotime, Shanghai, China), and then the sections were counterstained with hematoxylin and mounted using neutral resin. Three randomly selected fields of view were analyzed, and images were captured using an inverted microscope (CKX31, Olympus). The antibody information is shown in Table S3.

RT-q PCR. RT-qPCR was carried out on each group after 14 d. TRIzol Reagent (15596026CN, Invitrogen, US) was used to extract and purify RNA from treated cells, and the RNA concentration was assessed using Multiskan Go (Thermo Scientific, US). Samples were converted from RNA to

cDNA by reverse transcription using a PrimeScript RT Master Mix Kit (RR036A, Takara, Japan). SYBR Premix Ex Taq II (RR820A, Takara, Japan) was used for RT-qPCR analysis following the manufacturer's instructions. Experiments were conducted in triplicate and repeated three times. The primer sequences for each gene are listed in Table S2. GAPDH served as an endogenous control. The data were calculated as a fold change relative to the control group using the ΔΔCt method, and the filtering criteria for identifying up- or down-regulated genes were a $1 \leq \log_2$ (fold change) ≤ -1 and a p-value < 0.05.

Immunofluorescence. Immunofluorescence was used to determine the optimal concentration of EVs. CIE was co-cultured with C28/I2 at different concentrations for 48 h, whereas RIE was co-cultured with RAW264.7 at different concentrations for 3 d, followed by standard cell treatment procedures. RAW264.7 was incubated overnight at 4 °C with CD206 antibodies. C28/I2 was also incubated overnight at 4 °C with COL2 antibodies. The sections were then incubated with fluorescent-labeled secondary antibodies. The cell nuclei were counterstained with DAPI, and the cytoskeleton was counterstained with actin-tracker-rhodamine (C2207S; Beyotime, Shanghai, China). All staining results were imaged using an inverted laser confocal microscope (LSM880, ZEN) and quantified using ImageJ software (v1.51, NIH). The images were segmented into different fluorescence channels and converted into an 8-bit format, and the grayscale values were measured using appropriate thresholds.

Design and characterization of KM13E

The KM13 (Ac-KLDLKLDLGPLGMRGLDLKLDLKLDL-$CONH_2$) peptide was commercially synthesized by Shanghai Botai Biotechnology and purified to >95% purity by HPLC. The molecular weight (MW) of the peptide was determined by LC-MS. The theoretical isoelectric point was calculated using the Henderson-Hasselbalch equation: the lyophilized peptides were dissolved in deionized water to a concentration of 10 mg/mL (1%) for subsequent experiments.

SEM. To form a hydrogel, 100 μL of 1% (w/v) KM13 was co-incubated with DPBS for 2 h. The resulting hydrogel was immersed in 2.5% glutaraldehyde for 2 h and then dehydrated in a series of ethanol concentrations, starting at 30%, increasing incrementally to 50%, 70%, 80%, and 90%, and then twice in 100% ethanol. The dehydrated samples were subjected to critical point drying using liquid CO_2 and then gold-plated to a thickness of 10 nm. Subsequently, the micromorphology of

KM13 (1%) was analyzed using SEM (N7000, Hitachi). ImageJ software was used to perform particle size analysis in three different fields of view.

Rheological testing. A MARS III rheometer (HAAKE) equipped with a 20 mm parallel plate was used to perform rheological measurements on the hydrogels. The gelation time of the KM13 hydrogel (final concentration of 1%) was monitored at a frequency of 1 Hz and a strain of 1% for 1 h at room temperature. The gelation point was determined at the intersection of the storage modulus (G′) and loss modulus (G″). Moreover, the viscosity and shear stress of the KM13 hydrogels were measured at different shear rates ranging from 0.1 to 100 s^{-1} at 25 °C.

Enzymatic properties of KM13E

To prepare the samples, 1% KM13 was mixed with MMP13 (0.5 ng/ml, CC1047, Sigma, US) and incubated at 37 °C for 24 h.

TEM. The morphology of the peptides dispersed in the solution was analyzed by TEM. 10 μL of the peptide solution before and after enzyme treatment was dropped onto a copper grid and allowed to incubate at room temperature for 10 min. The grid was then subjected to negative staining with 2% uranyl acetate and dried on the edge of filter paper. Finally, the prepared samples were imaged at 80 kV using a TEM (H-7650; Hitachi).

AFM. Atomic force microscopy (AFM) was used to measure the length of the peptide nanofibers. Separately, 10 μL of the peptide solution was placed on matching mica substrates before and after the enzyme treatment. The mica substrate was washed with deionized water and air-dried at room temperature. The prepared sample was then analyzed using AFM (Cipher VRS) in AC mode at a scanning speed of 0.5 Hz.

Rheological testing. The rheological characteristics of the hydrogel samples were evaluated by dynamic frequency sweep testing at room temperature in the frequency range of 0.1–10 rad/s. To ensure that the strain magnitude used was within the linear viscoelastic region of the hydrogel samples, a dynamic amplitude sweep test was conducted at a frequency of 6.28 rad/s, with shear amplitudes ranging from 0.1% to 10%. The viscosity and shear stress of KM13 hydrogels were measured at increasing shear rates from 0.1 to 100 s-1 at 25 °C.

Tribological test. Tribological tests were carried out using a universal material tester (UMT-3, Bruker Nano Inc.) in reciprocating mode at room temperature for 600 cycles. The test employed

pin-on-disk friction pairs, with a polytetrafluoroethylene pin (contact surface diameter: 5 mm) serving as the upper sample and a silicon wafer as the lower sample. Before each experiment, 20 drops of the sample solution were added to the contact area as a lubricant, with PBS and HA (H10960136, Schweppert, Shangdong, China) serving as control groups. The oscillation amplitude was 4 mm, sliding frequency was 1 Hz, and normal load was 12 N. To ensure data validity, each test was repeated at least three times.

ELISA assay of MMP13 concentration

To determine the concentration levels of MMP13 in the joint fluids of healthy Sprague-Dawley (SD) and OA rats, MMP13 ELISA kits (Cloud-clone, China) were used according to the manufacturer's instructions.

MMP13 activity assay

The activity levels of MMP13 in the joint fluid were determined in healthy SD and OA rats using fluorescence quantification of the Metalloproteinase 13 Activity Kit (50076.2, Jisskang), following the instructions provided by the manufacturer. Briefly, the joint fluid was extracted from the joint cavities of healthy SD and OA rats. The extracted fluid was then mixed with Kormas Brilliant Blue G-250-labeled enzymatic substrate reagent E at concentrations of 1, 0.5, 0.25, 0.125, 0.0625, and 0.0317 mg/ml. The MMP13 concentration in each sample group was adjusted to match the ELISA value of MMP13 in the joint fluid. Next, 20 μl of activated MMP13 and joint fluid were added to the respective gradient concentrations of enzyme cutting substrate reagent E. The mixture was mixed for 1 min, and the OD value at 595 nm was measured to calculate the enzyme activity relative to activated MMP13.

Specific MMPs responsiveness of KM13E

To confirm the specific response of KM13E to MMP13, KM13E1.5 was mixed with 1 mL of each MMP (MMP1, MMP3, MMP9, and MMP13). Each enzyme was present in the SBF at a concentration of 0.5 ng/mL. The solutions were incubated at 37 °C, and 100 μL of each solution was collected at 3-day intervals to measure protein concentration over an 18-day period. After each collection, 100 μL of the corresponding degradation solution was added to replenish the solution. The protein concentration was determined using a BCA kit with three replicates per group. The release rate was calculated using the formula described previously.

Characterization of PEGDA/GelMA hydrogel

NMR spectroscopy of the GelMA prepolymer. For the NMR spectroscopy, the GelMA sample was dissolved in deuterium oxide and stirred for 2 h to ensure homogeneity. ^{1}H NMR spectra were recorded at room temperature using a Bruker-400 NMR instrument operating at a frequency of 400 MHz. To accurately determine the chemical shifts, deuterium oxide was used as an internal standard.

FT-IR of the PG hydrogel. The specific chemical groups of the GelMA, PEGDA, and PG hydrogels were examined by Fourier transform infrared (FT-IR) spectroscopy on a Vertex 70v instrument (Bruker, Germany). Hydrogels were initially subjected to freezing at -80 °C, followed by lyophilization, pulverization, and subsequent mixing with potassium bromide in a weight ratio of 1:100 (w/w). The resulting mixtures were compressed into disks using a tablet press. Subsequently, FT-IR analysis was performed by scanning the samples from 500 to 4000 cm^{-1} at a resolution of 2 cm^{-1}. Each sample was subjected to 140 scans to acquire the FT-IR spectrum, which was subsequently averaged across all the scans to ensure accurate characterization.

Rheological properties of the KGE hydrogel. To evaluate the rheological properties, PG hydrogels were formed by mixing GelMA (final concentration 5%) with PEGDA (final concentration 5%) under 365-nm UV light for 10 s. Samples were subjected to dynamic frequency sweep testing at room temperature, with a test frequency ranging from 0.1 to 10 rad/s. To ensure accurate results, the strain magnitude utilized was within the linear viscoelastic region, which was determined by dynamic amplitude sweep testing of the PG hydrogel samples. The test was conducted at a frequency of 6.28 rad/s, with shear amplitudes ranging from 0.1% to 10%.

Preparation and characterization of PGE microspheres

Microfluidic chips and materials. A microfluidic chip and multifunctional fluidic programmer were purchased from Meso Bio Systems, and specific fluidic channels within the chip were described in our previous study[4]. Microfluidic technology was used to prepare the PGE microspheres. GelMA (final concentration 5%), CIE (final concentrations 0.5, 1, 1.5, and 2 mg/mL), and PEGDA (final concentration 5%) were dissolved in deionized water, and 0.5% w/v lithium phenyl-2,4,6-trimethylbenzoylphosphonate was used as the photoinitiator to form the water-phase solution. The oil-phase solution was mineral oil containing 10% (v/v) Span 80.

Preparation of microspheres. Oil- and water-phase solutions were sequentially injected into the

two inlets of the microfluidic chip. As the relative pressure of the water phase was adjusted, mixed droplets (oil-in-water) formed at the intersection and were mobilized toward the outlet. UV irradiation at 365 nm for 15 s within the chip was used to crosslink the hydrogel microspheres. The resultant microspheres were rinsed with deionized water, transferred into a 1.5-mL Eppendorf tube, and centrifuged at 5000 rpm for 15 min (this process was repeated three times). The microspheres are then resuspended in deionized water and stored at 4 °C. Scanning electron microscopy (SEM) was used to observe the microstructure of the microspheres.

EVs release analysis from PGE microspheres and KM13E hydrogel

BCA analysis. To monitor the release of EVs from the KM13E and PGE microspheres, their release characteristics were investigated in SBF (CZ0403, Leagene Corporation, Beijing, China), degradation solution consisting of SBF supplemented with 0.5 ng/mL MMP13 enzyme, and DMEM medium. Each group of hydrogel and microspheres was added to 1 mL of the respective degradation solution at 37 °C, and 100 μL of the solution was collected every 3 d up to 18 d to measure protein concentration. After each collection, 100 μL of the corresponding degradation solution was added to replenish the solution. The BCA kit was used to determine the protein concentration, with three replicates per group. The formula used to calculate the release rate was derived from a previous study[4].

Fluorescent assay. To visualize the release of EVs in the microspheres, PKH26-labeled CIE (2 mg/mL) was integrated into the water phase, and microspheres were ultimately generated using the same method. 2 μL of PGE2 microspheres were combined with 2 mL of SBF or SBF supplemented with 0.5 ng/mL of MMP13 enzyme to observe a single microsphere. The fluorescence of each group was observed at defined intervals of 0, 3, 6, 9, 12, 15, and 18 d and tracked using an inverted laser confocal microscope. The relative fluorescence intensity of the microspheres was calculated by visualizing the obtained fluorescence images using the ZEN software (v2.0, Zeiss).

***In vitro* release assay**

Mixing PGE2 with KM13E1.5 results in the formation of a KM13E@PGE composite system, where CIE is labeled with PKH67 and RIE is labeled with PKH26. The structure of the composite system was observed under a light microscope.

ELISA. Based on the results of the above experiments, IL-10 was used as a marker for RIE, and

SOX9 was used as a marker for CIE. These were used to track the release of EVs in the composite system. The relative quantities of EV release with SOX9 and IL-10 ELISA kits were recorded at defined time intervals of 0, 3, 6, 9, 12, 15, and 18 d. The ELISA kit information is shown in Table S4.

EVs' uptake. To track the uptake rate of EVs by RAW264.7 and C28/I2 cells *in vitro*, both cells were inoculated in 6-well plates at a density of 1×10^4 cells/mL, and the cells were cultured in a proliferation medium containing 5 ng/mL of MMP13 and semi-changed every 2 d. KM13E@PGE was co-cultured with these two cell lines for 5 d. After 5 d, KM13@PGE and the culture medium were removed and transferred to wells, where the two cells were re-inoculated. These steps were repeated for 30 d. Every 3 d, the cells were washed with DPBS and fixed with 4% paraformaldehyde (PFA, P0099, Beyotime, Shanghai, China) for Immunofluorescence, and DAPI was used to visualize the nuclei. Three different samples from each group were observed and photographed using an inverted laser confocal microscop. Images were analyzed using ImageJ software to calculate the relative fluorescence intensity. Given that RIE fluorescence intensity was difficult to observe when it was low, only fluorescence images from days 6, 12, and 18 are displayed. The fluorescence intensity within 30 d was collected and counted.

***In vivo* imaging**

To visualize the biodistribution of KM13E@PGE in live organisms, 30 six-week-old male SD rats were used. Among these rats, 15 were specifically used to construct *in vivo* OA release models, as detailed in this section.

Five minutes after the injection of KM13E@PGE, KM13E, PGE, RIE, and CIE, all animals were photographed under bright vision using an *in vivo* imaging system (FX Pro, Bruker). Fluorescent images were obtained using excitation (470/30 nm and 550/30 nm) and emission filters (535/20 nm and 600/20 nm). The exposure time was set to 1 s to avoid overexposing the images. Specifically, animals in the KM13E@PGE group were set to receive both fluorescence and bright field shots sequentially. All experimental animals were subjected to *in vitro* fluorescence imaging in the following weeks. Fluorograms were quantified using image analysis software (Bruker MI SE v721, Bruker).

Animal experiment

After conducting a pre-experimental exploration, 40 male SD rats were procured from the Animal Center of Southern Medical University. These rats were kept in a controlled environment at a temperature range of 18–25 °C, a humidity level of 40–70%, and a 12-h light/dark cycle (8:00–20:00), with access to sufficient food and water. The bedding, consisting of corncobs and shavings, was changed 2–3 times per week to maintain cleanliness.

Using a random number table, the rats were divided into nine groups: blank, OA, KM13E, PGE, Group I-IV, and KM13E@PGE. Based on this grouping, the therapeutic ability of the KM13E@PGE composite system was evaluated in a rat model of OA. Briefly, the left joint cavity of each 2-week-old rat was injected with 0.2 mL of iodoacetic acid (MIA, 5 g/mL, I2512, Sigma, US). No post-injection complications, such as wound infections or difficulty eating were observed, nor were any significant changes in the body weight or size of the rats. Plantar pain thresholds were assessed in the rats before and after sampling. The rats were euthanized 2 and 4 weeks after injection, and their joint tissues were separated using a handheld chainsaw, gently rinsed in saline, and fixed in 4% PFA for subsequent evaluation. All animals survived until the time of sampling and were included in the subsequent experiments.

Histological testing

Fixed joint tissues (n = 40) were washed three times with DPBS for 20 min each to remove any residue. The tissues were then immersed in an EDTA decalcification solution (R20403; Leagene, Beijing, China) and left to decalcify for one month. The solution was changed once a week, and the decalcification endpoint was checked using a pinprick. After adequate decalcification, the tissues were thoroughly rinsed with distilled water before ethanol gradient dehydration and clearance with xylene. The cleared tissues were embedded in wax and cut into 5-μm tissue sections. Standard hematoxylin and eosin (H&E), Masson, Alcian blue, and Safranin O-Fast Green staining were performed to investigate the structural features of the cartilage tissue. Chondrocyte survival was evaluated by researchers blind to the experimental groups.

OARSI score. In accordance with the ORASI guidelines[6], two researchers who were blind to the experimental groups performed the histological scoring of the three most severely affected sections.

Relative GAG level. Relative GAG levels were assessed by analyzing the staining depth and tissue

area using ImageJ software (v1.51, National Institutes of Health) following Alcian blue staining. The average GAG level for each group was determined and subsequently compared with that of the blank group.

Collagen volume fraction. After staining with Masson, the tissues were analyzed to measure the depth of staining and the percentage area covered by blue fibers using ImageJ software (v1.51, NIH). The data obtained from these measurements were then multiplied. Three different areas were selected for replication in each group.

Immunohistochemical staining. ACAN staining was used to identify the mature cartilage tissue. After routine pre-treatment, sections were incubated with anti-aggrecan (1:200, ab36861, Abcam, UK) antibody for 10 h at 4 °C, followed by goat anti-rabbit IgG H&L (HRP) (1:500, ab6721, Abcam, UK) for 1 h at room temperature. Next, OCN staining was performed using the 3,3'-diaminobenzidine tetrahydrochloride kit (P0202; Beyotime, Shanghai, China). Counterstaining was performed with hematoxylin, and mounting was performed using neutral resin. Images were captured using a CKX31 microscope (OLYMPUS). Three randomly selected fields of view were analyzed. ACAN protein expression levels were assessed using the ImageJ IHC Profiler plugin. The software deconvoluted the DAB/hematoxylin chromatogram and performed downstream analysis to obtain aggrecan-positive areas.

Immunofluorescent staining. The sections underwent standard procedures and were then incubated overnight at 4 °C with anti-CD86 (1:200, ab238468, Abcam, UK) and anti-CD206 (1:1000, ab64693, Abcam, UK) antibodies. To gauge the extent of inflammatory factor infiltration and cartilage tissue repair, the sections were similarly incubated overnight at 4 °C with anti-TNF-α (1:100, ab205587, Abcam, UK) and anti-SOX9 (1:500, ab185966, Abcam, UK) antibodies. The corresponding secondary antibodies were applied, and the nuclei were counterstained with DAPI. All staining outcomes were visualized using a CKX31 microscope and quantified using ImageJ software (v1.51, NIH). Specifically, the images were segmented into distinct fluorescent channels and converted to an 8-bit format, and the gray-scale values were measured after setting suitable thresholds.

Statistical analysis

The study utilized a minimum of three biological replicates for each group and presented continuous variable data as mean ± SD. IBM SPSS Statistics 27.0 was used for statistical analyses. Student's t-

test was used to compare means between two groups, and one-way analysis of variance (ANOVA) or Welch's test was used to compare means among multiple groups. Multiple comparison correction was performed using the Bonferroni correction. Statistical significance was set at $p < 0.05$.

References

[1] Q. Fu, R. Zhou, J. Cao, Y. Chen, J. Zhu, Y. Zhou, J. Shao, W. Xin, S. Yuan, Front Bioeng Biotechnol **2022**, 10, 889306.

[2] M. Dominici, K. Le Blanc, I. Mueller, I. Slaper-Cortenbach, F. Marini, D. Krause, R. Deans, A. Keating, D. Prockop, E. Horwitz, Cytotherapy **2006**, 8, 315.

[3] T. Tang, B. Wang, M. Wu, Z. Li, Y. Feng, J. Cao, D. Yin, H. Liu, R. Tang, S. D. Crowley, L. Lv, B. Liu, Sci Adv **2020**, 6, eaaz0748.

[4] Y. Yang, W. Zheng, W. Tan, X. Wu, Z. Dai, Z. Li, Z. Yan, Y. Ji, Y. Wang, W. Su, S. Zhong, Y. Li, Y. Sun, S. Li, W. Huang, Acta Biomater. **2023**, 157, 321.

[5] J. A. Welsh, D. C. I. Goberdhan, L. O'Driscoll, E. I. Buzas, C. Blenkiron, B. Bussolati, H. Cai, D. Di Vizio, T. A. P. Driedonks, U. Erdbrügger, J. M. Falcon-Perez, Q. Fu, A. F. Hill, M. Lenassi, S. K. Lim, M. G. Mahoney, S. Mohanty, A. Möller, R. Nieuwland, T. Ochiya, S. Sahoo, A. C. Torrecilhas, L. Zheng, A. Zijlstra, S. Abuelreich, R. Bagabas, P. Bergese, E. M. Bridges, M. Brucale, D. Burger, R. P. Carney, E. Cocucci, R. Crescitelli, E. Hanser, A. L. Harris, N. J. Haughey, A. Hendrix, A. R. Ivanov, T. Jovanovic-Talisman, N. A. Kruh-Garcia, V. Ku'Ulei-Lyn Faustino, D. Kyburz, C. Lässer, K. M. Lennon, J. Lötvall, A. L. Maddox, E. S. Martens-Uzunova, R. R. Mizenko, L. A. Newman, A. Ridolfi, E. Rohde, T. Rojalin, A. Rowland, A. Saftics, U. S. Sandau, J. A. Saugstad, F. Shekari, S. Swift, D. Ter-Ovanesyan, J. P. Tosar, Z. Useckaite, F. Valle, Z. Varga, E. van der Pol, M. J. C. van Herwijnen, M. H. M. Wauben, A. M. Wehman, S. Williams, A. Zendrini, A. J. Zimmerman, C. Théry, K. W. Witwer, J Extracell Vesicles **2024**, 13, e12404.

[6] N. Gerwin, A. M. Bendele, S. Glasson, C. S. Carlson, Osteoarthr. Cartilage **2010**, 18, S24.

Supplementary Tables

Table S1. Abbreviation index

Full Name	Abbreviation
Metalloproteinase with thrombospondin motifs	ADAMTS
Atomic force microscope	AFM
C-C motif chemokine ligand 8	CCL18
C-IPFP-MSCs-derived EVs (SOX9^{+} EVs)	CIE
Chondroinduced IPFP-MSCs	C-IPFP-MSC
Coefficient of friction	COF
Type II collagen	COL2
Differentially expressed proteins	DEP
Extracellular matrix	ECM

Extracellular vesicles	EVs
Forkhead box O1	FOXO1
Forkhead box O3	FOXO3A
Growth differentiation factor 5	GDF5
GLI family zinc finger 1	GLI1
Hyaluronic acid	HA
Hypoxia inducible factor 1 subunit alpha	HIF1A
IPFP-MSCs-derived EVs	IE
Interleukin-10	IL-10
Interleukin-1β	IL-1β
Interleukin-6	IL-6
Nitric oxide synthase	iNOS
Infrapatellar fat pad	IPFP
Infrapatellar fat pad-derived mesenchymal stem cells	IPFP-MSCs
Integrin subunit alpha 4	ITGα4
Integrin subunit alpha M	ITGαM
Integrin subunit beta 1	ITGβ1
Integrin subunit beta 2	ITGβ2
KLDL-MMP13	KM13
Iodoacetic acid	MIA
Matrix metalloproteinase 13	MMP13
Matrix metalloproteinase	MMPs
Mesenchymal stem cell-derived EVs	MSC-EVs
Nanoparticle Tracking Analysis	NTA
Osteoarthritis	OA
POU class 5 homeobox 1	OCT-4
PEGDA/GelMA microspheres	PG
PEGDA/GelMA/CIE microspheres	PGE
Recombination signal binding protein for immunoglobulin Kappa J region	RBPJ
WT-RAW-derived EVs	RE
RELA proto-oncogene	RELA
Full Name	Abbreviation
IL-10+RAW-derived EVs (IL-10^{+} EVs)	RIE
Quantitative PCR with reverse transcription	RT-qPCR
RUNX family transcription factor 2	RUNX2
Self-assembling peptides	SAPs
Simulated body fluid	SBF
Sprague-Dawley rat	SD rat
Scanning electron microscope	SEM
SRY-box transcription factor 9	SOX9
Transmission electron microscopy	TEM
Tumor necrosis factor alpha	TNF-α

Table S2. Primer sequences used for RT-qPCR

Gene	Primer Sequences (F = forward; R = reverse)

IL10	F: TTAATAAGCTCCAAGACCAAGG
	R: CATCATGTATGCTTCTATGCAG
GAPDH	F: ACTCTTCCACCTTCGATGC
	R: CCGTATTCATTGTCATACCAGG
ACAN	F: CTGACCAGACTGTCAGATACC
	R: TCCTCACACCAGGAAACTC
COL2A1	F: AGGAGACAGAGGAGAAGCT
	R: CTTGAGGACCCTGGATTCC
SOX9	F: CTCTGGAGACTTCTGAACGA
	R: ACTTGTAATCCGGGTGGTC
COL10A1	F: GATACCAAATGCCCACAGG
	R: CCTCTTACTGCTATACCTTTACTC
SOX5	F: TTCAGCTGATCCCAACTACC
	R: CATATAACTGCTGCAGTTGGAG
SOX6	F: TACCAACACTTGTCAGTACCA
	R: TCTCTGATTCCATTCTTTGCTG
GLI1	F: TTTCATCAACTCGCGATGC
	R: GAATCCCAGAGATGGGCTC
RUNX2	F: TTATTCTGCTGAGCTCCGG
	R: GTGAAACTCTTGCCTCGTC
RELA	F: GCTTGTAGGAAAGGACTGC
	R: AGGTTCTGGAAACTGTGGA
HIF1A	F: GTTCTCACAGATGATGGTGAC
	R: TGTCCAGTTAGTTCAAACTGAG
RBPJ	F: CTTACTAGGGAAGCTATGCG
	R: TTCCATATGACTTCTGTGCA
FOXO3A	F: ATCTACGAGTGGATGGTGC
	R: CCGGATGGAGTTCTTCCAG

Table S3. Antibody

Product Name	Item Number	Manufacturer	Dilution
FITC Anti-CD105	ab11415	Abcam	Flow Cyt: 1/10
PE Anti-CD90	ab95700	Abcam	Flow Cyt: 1/20
FITC Anti-CD73	ab239246	Abcam	Flow Cyt: 1/20
FITC Anti-CD34	ab78165	Abcam	Flow Cyt: 1/20
PE Anti-CD45	ab134202	Abcam	Flow Cyt: 1/10
FITC Anti-HLA-DR	ab1182	Abcam	Flow Cyt: 1/40
Anti-CD9	ab236630	Abcam	WB: 1/1000
Anti-CD81	ab109201	Abcam	WB: 1/5000
Anti-ALIX	ab186429	Abcam	WB: 1/5000
Anti-Calnexin	ab133615	Abcam	WB: 1/1000
Anti-CD206	ab64693	Abcam	IF: 1/1000
Anti-Collagen II	ab34712	Abcam	IF: 1/500; IHC: 1/200
Anti-Aggrecan	ab36861	Abcam	IHC: 1/200

Anti-SOX9	ab185966	Abcam	IHC: 1/1000; IF: 1/500
Anti-CD86	ab238468	Abcam	IF: 1/200
Anti-TNF-α	ab205587	Abcam	IF: 1/100

Table S4. ELISA kit

Protein	Item Number	Manufacturer
IL10 (Mus)	SEA056Mu	Cloud-Clone corp.
iNOS	SEA837Mu	Cloud-Clone corp.
CCL18	SEB522Mu	Cloud-Clone corp.
ITGα4	EKU05033	Biomatik
ARG1	SEB120Mu	Cloud-Clone corp.
ITGβ1	EKU09042	Biomatik
ITGβ2	EKU05042	Biomatik
ITGαM	EKE61801	Biomatik
GDF5	SEC110Hu	Cloud-Clone corp.
SOX9	SEG329Hu	Cloud-Clone corp.
FOXO1	SEA764Hu	Cloud-Clone corp.
OCT4	SEA424Hu	Cloud-Clone corp.
IL10 (Rat)	EKU05181	Biomatik
MMP13	SEA099Ra	Cloud-Clone corp.
TNFα	SCA133Ra	Cloud-Clone corp.

Table S5. List of DEP from proteomics analysis (RE vs RIE)

Gene	RE1	RE2	RE3	RIE1	RIE2	RIE3
**IL10**	-0.913	-0.918	-0.885	0.910	0.918	0.938
Ppp6c	0.635	1.165	0.900	-0.924	-0.876	-0.900
Ppic	-0.632	-1.058	-0.845	0.284	1.405	0.845
Ppm1g	0.815	1.008	0.911	-0.900	-0.922	-0.911
Psmg1	0.845	0.978	0.912	-0.945	-0.879	-0.912
Rpl38	0.794	1.026	0.910	-0.903	-0.918	-0.910
Polr3h	1.008	0.814	0.911	-0.911	-0.911	-0.911
Sec24a	0.612	1.184	0.898	-0.908	-0.887	-0.898
Samd9l	0.130	-0.173	-0.022	1.595	-1.552	0.022
Scly	-0.952	-0.862	-0.907	0.732	1.082	0.907
Sart3	0.810	1.012	0.911	-0.954	-0.867	-0.911
Septin6	1.284	0.481	0.882	-0.822	-0.943	-0.882
Slfn9	-0.816	-0.962	-0.889	1.240	0.538	0.889
Slc7a1	0.937	0.888	0.912	-0.964	-0.861	-0.912
Timm50	0.694	1.100	0.897	-1.108	-0.687	-0.897
Mlec	0.883	0.941	0.912	-0.950	-0.875	-0.912
Mapk1	0.889	0.935	0.912	-0.953	-0.872	-0.912
Nup85	0.870	0.870	0.870	-1.350	-0.389	-0.870

Pdia3	0.822	1.001	0.911	-0.919	-0.904	-0.911
Pla2g15	-0.998	-0.822	-0.910	0.827	0.993	0.910
Pi4kb	-0.559	-0.967	-0.763	-0.081	1.607	0.763
Rps8	0.786	1.033	0.910	-0.961	-0.858	-0.910
Sorl1	0.743	1.072	0.907	-0.961	-0.854	-0.907
Smc4	-0.974	-0.836	-0.905	0.711	1.099	0.905
Sin3a	-0.793	1.117	0.162	1.066	-1.391	-0.162
Gtf2b	0.733	1.065	0.899	-0.681	-1.117	-0.899
Cct6a	0.866	0.959	0.912	-0.929	-0.896	-0.912
Tor1a	1.548	0.060	0.804	-0.880	-0.728	-0.804
Ric8a	-0.927	-0.838	-0.883	0.481	1.284	0.883
Arhgap12	-0.234	-0.978	-0.606	1.728	-0.516	0.606
Slc12a7	0.583	1.202	0.892	-0.772	-1.013	-0.892
Scarb1	0.843	0.980	0.911	-0.966	-0.857	-0.911
Samhd1	0.832	0.992	0.912	-0.890	-0.934	-0.912
Sec61a1	0.771	1.047	0.909	-0.897	-0.922	-0.909
Phgdh	0.750	1.066	0.908	-0.919	-0.898	-0.908
Sf3a2	0.628	1.170	0.899	-0.910	-0.889	-0.899
Syngr1	1.796	-0.616	0.590	-0.639	-0.541	-0.590
Smarca4	0.810	1.004	0.907	-1.053	-0.761	-0.907
Snrpd1	0.824	0.998	0.911	-0.958	-0.864	-0.911
Smn1	0.291	1.415	0.853	-0.853	-0.853	-0.853
Stxbp3	0.889	0.936	0.913	-0.933	-0.892	-0.913
Stx6	0.929	0.897	0.913	-0.921	-0.905	-0.913
Spsb2	1.001	0.818	0.910	-1.009	-0.810	-0.910
Farsa	0.940	0.884	0.912	-0.958	-0.866	-0.912
Prpf8	0.684	1.121	0.902	-1.000	-0.805	-0.902
Alpl	-0.895	-0.904	-0.900	1.169	0.630	0.900
Rbmx	0.738	1.077	0.908	-0.931	-0.884	-0.908
Rpl3	0.899	0.926	0.913	-0.929	-0.897	-0.913
Rpn2	0.858	0.967	0.912	-0.929	-0.895	-0.912
Gm2a	-1.330	-0.232	-0.781	0.174	1.388	0.781
Selenbp1	0.809	1.013	0.911	-0.932	-0.889	-0.911
Set	0.592	1.199	0.895	-0.944	-0.846	-0.895
Seh1l	-1.787	0.720	-0.534	0.807	0.261	0.534
Pls3	0.882	0.942	0.912	-0.978	-0.846	-0.912
Ppm1a	0.752	1.051	0.902	-1.099	-0.704	-0.902
Ptpn7	0.679	0.987	0.833	-0.206	-1.460	-0.833
Psmd2	-1.283	-0.176	-0.730	-0.043	1.502	0.730
Uqcrc2	0.681	1.127	0.904	-0.877	-0.931	-0.904
Rcc1	0.797	1.024	0.910	-0.948	-0.873	-0.910
Rad23b	0.887	0.938	0.913	-0.913	-0.913	-0.913
Rab1A	0.797	1.023	0.910	-0.944	-0.877	-0.910
Rpl35a	0.217	1.460	0.839	-0.783	-0.895	-0.839

Rrp7a	-0.624	-1.129	-0.876	0.514	1.239	0.876
Rps15	-0.723	-1.074	-0.898	0.681	1.116	0.898
Rps26	0.649	1.153	0.901	-0.938	-0.864	-0.901
Setd3	0.968	0.856	0.912	-0.951	-0.873	-0.912
Syngr2	1.066	0.742	0.904	-1.058	-0.749	-0.904
Spcs2	0.816	1.006	0.911	-0.874	-0.948	-0.911
Snap23	0.928	0.895	0.911	-1.003	-0.819	-0.911
Snx6	0.419	1.278	0.849	-1.242	-0.455	-0.849
Smc1a	0.744	1.070	0.907	-0.834	-0.980	-0.907
Srgap2	0.733	1.080	0.907	-0.968	-0.845	-0.907
St14	0.785	1.033	0.909	-0.976	-0.843	-0.909
Stag2	0.613	1.181	0.897	-0.828	-0.967	-0.897
Srsf1	-0.722	1.306	0.292	-1.395	0.811	-0.292
Tgfb1	0.627	1.171	0.899	-0.899	-0.899	-0.899
Pnp	0.673	1.133	0.903	-0.949	-0.857	-0.903
Slc39a10	0.938	0.887	0.912	-0.951	-0.874	-0.912
Septin2	0.783	1.037	0.910	-0.934	-0.886	-0.910
Septin7	0.743	1.073	0.908	-0.922	-0.894	-0.908
Smarcc2	-0.652	-1.138	-0.895	0.701	1.089	0.895
Serpinb6	0.702	1.104	0.903	-1.020	-0.786	-0.903
Srm	0.516	1.250	0.883	-0.716	-1.049	-0.883
Smchd1	-0.917	-0.906	-0.911	0.816	1.006	0.911
Stxbp2	0.815	1.008	0.911	-0.919	-0.903	-0.911
Swap70	0.932	0.893	0.913	-0.873	-0.952	-0.913
Srp14	0.645	1.150	0.898	-1.033	-0.762	-0.898
Srrt	0.557	1.225	0.891	-0.983	-0.799	-0.891
Sars1	0.753	1.061	0.907	-1.004	-0.810	-0.907
Tbcd	0.704	1.106	0.905	-0.939	-0.872	-0.905
Tpm4	-1.036	1.815	0.389	-0.507	-0.271	-0.389
Tpm3	0.732	1.082	0.907	-0.946	-0.868	-0.907
Relt	0.845	0.954	0.900	-1.162	-0.638	-0.900
Cd40	-0.890	-0.591	-0.741	-0.172	1.653	0.741
Tnfrsf22	0.629	1.151	0.890	-1.128	-0.651	-0.890
Tmem87a	0.711	1.099	0.905	-0.988	-0.821	-0.905
Nup98	-0.546	-0.662	-0.604	1.788	-0.581	0.604
Parvb	0.768	1.049	0.909	-0.867	-0.951	-0.909
Ppp4r2	-1.194	-0.592	-0.893	0.756	1.029	0.893
Hnrnpa2b1	0.986	0.837	0.912	-0.879	-0.944	-0.912
Rpp30	-1.012	-0.746	-0.879	0.476	1.282	0.879
Tars1	0.839	0.984	0.912	-0.934	-0.890	-0.912
Slc19a1	0.709	1.102	0.906	-0.910	-0.901	-0.906
Psap	-1.298	-0.097	-0.698	-0.126	1.522	0.698
Spcs3	0.687	1.103	0.895	-1.124	-0.667	-0.895
Tbca	1.084	0.730	0.907	-0.907	-0.907	-0.907

Cct4	0.640	1.113	0.877	-1.250	-0.504	-0.877
Ptk2	0.814	1.008	0.911	-0.890	-0.932	-0.911
Exosc6	0.693	1.116	0.904	-0.938	-0.871	-0.904
Eri3	1.360	0.116	0.738	-0.045	-1.431	-0.738
Exoc4	-1.465	-0.143	-0.804	0.453	1.155	0.804
Ext1	1.087	0.726	0.907	-0.906	-0.908	-0.907
Gatd1	1.209	0.580	0.894	-0.942	-0.846	-0.894
Glt8d1	-1.381	-0.339	-0.860	0.760	0.960	0.860
Itsn2	0.913	0.912	0.913	-0.877	-0.948	-0.913
Kdsr	0.924	0.902	0.913	-0.902	-0.924	-0.913
Lamtor2	0.758	1.059	0.909	-0.902	-0.916	-0.909
Pa2g4	0.710	1.101	0.905	-0.967	-0.843	-0.905
Ppp2ca	1.026	0.608	0.817	-1.490	-0.144	-0.817
Pik3c3	0.524	1.246	0.885	-1.028	-0.742	-0.885
Pon1	0.893	0.932	0.913	-0.936	-0.889	-0.913
Rpl32	0.270	1.425	0.847	-0.961	-0.734	-0.847
Rnf123	1.695	-0.299	0.698	-0.907	-0.489	-0.698
Rnf11	-0.574	1.648	0.537	-1.170	0.096	-0.537
Rras2	0.819	1.003	0.911	-0.914	-0.909	-0.911
S100a4	0.880	0.946	0.913	-0.909	-0.916	-0.913
Scamp1	0.885	0.940	0.913	-0.906	-0.919	-0.913
Snf8	0.641	1.160	0.900	-0.868	-0.933	-0.900
Smarca5	0.758	1.058	0.908	-0.967	-0.849	-0.908
Dars1	0.658	1.145	0.902	-0.939	-0.864	-0.902
Tars3	-0.854	-0.676	-0.765	-0.093	1.623	0.765
Psmb9	1.026	0.792	0.909	-0.837	-0.982	-0.909
Adss1	0.847	0.977	0.912	-0.935	-0.889	-0.912
Nras	0.984	0.839	0.912	-0.955	-0.868	-0.912
Rpa1	0.838	0.986	0.912	-0.933	-0.890	-0.912
Rpl7	0.767	1.051	0.909	-0.924	-0.895	-0.909
Sec11a	0.872	0.953	0.912	-0.936	-0.889	-0.912
Nos2	0.924	0.902	0.913	-0.914	-0.911	-0.913
Sdc4	0.585	1.205	0.895	-0.866	-0.924	-0.895
Sf3b4	0.118	1.500	0.809	-1.052	-0.565	-0.809
Sf3b3	0.711	1.099	0.905	-0.976	-0.834	-0.905
Scp2	0.837	0.986	0.912	-0.912	-0.912	-0.912
Pik3r4	-0.835	1.692	0.429	-1.022	0.164	-0.429
Ndrg3	0.851	0.973	0.912	-0.928	-0.897	-0.912
Nono	0.593	1.199	0.896	-0.900	-0.892	-0.896
Nap1l4	0.916	0.910	0.913	-0.956	-0.869	-0.913
Osbp	0.857	0.967	0.912	-0.905	-0.919	-0.912
Park7	0.873	0.952	0.913	-0.924	-0.901	-0.913
Pcyt2	1.123	0.684	0.904	-0.934	-0.873	-0.904
Ppp6r1	-0.912	-0.912	-0.912	0.990	0.833	0.912

Ssr3	0.660	1.145	0.902	-0.902	-0.902	-0.902
Stambp	0.965	0.859	0.912	-0.973	-0.850	-0.912
Spred1	0.774	1.045	0.909	-0.925	-0.894	-0.909
Tuba1a	-0.810	-1.009	-0.910	0.818	1.001	0.910
Tuba1c	0.702	1.107	0.905	-0.967	-0.843	-0.905
Taf6	1.332	0.403	0.867	-0.702	-1.033	-0.867
Tbcb	0.357	1.366	0.862	-0.727	-0.997	-0.862
Thg1l	0.777	1.042	0.910	-0.912	-0.907	-0.910
Top1	0.879	0.947	0.913	-0.928	-0.898	-0.913
Ttc27	1.351	0.382	0.867	-0.976	-0.757	-0.867
Rab18	0.706	1.105	0.906	-0.924	-0.887	-0.906
Polr1d	0.693	1.104	0.898	-1.093	-0.703	-0.898
Rps15a	0.917	0.908	0.913	-0.944	-0.881	-0.913
Rsbn1	-1.047	0.708	-0.170	-1.112	1.452	0.170
Rps28	-1.716	0.320	-0.698	0.748	0.648	0.698
Rps27l	0.785	1.029	0.907	-1.033	-0.781	-0.907
Snx18	0.725	1.088	0.907	-0.939	-0.875	-0.907
Inpp5d	0.576	1.211	0.893	-0.816	-0.970	-0.893
Eprs1	0.660	1.132	0.896	-1.085	-0.707	-0.896
Wars1	-0.937	-0.878	-0.908	0.743	1.072	0.908
Ufsp2	0.855	0.967	0.911	-1.003	-0.818	-0.911
Oit3	0.584	1.171	0.877	-0.555	-1.200	-0.877
Pigr	0.849	0.975	0.912	-0.916	-0.908	-0.912
Ppp1r14b	1.228	0.556	0.892	-0.898	-0.886	-0.892
Ppme1	0.898	0.928	0.913	-0.921	-0.905	-0.913
Rtcb	0.736	1.078	0.907	-0.975	-0.838	-0.907
Son	1.234	0.545	0.890	-0.968	-0.811	-0.890
Smarcd2	0.472	1.291	0.881	-0.846	-0.917	-0.881
Inip	0.944	0.882	0.913	-0.913	-0.913	-0.913
Skap2	0.692	1.090	0.891	-1.171	-0.611	-0.891
Stard5	0.839	0.944	0.892	-0.557	-1.227	-0.892
Stat1	0.526	1.250	0.888	-0.855	-0.922	-0.888
Stip1	0.888	0.937	0.913	-0.903	-0.923	-0.913
Farsb	0.847	0.976	0.911	-0.979	-0.844	-0.911
Tpr	-1.683	1.296	-0.193	0.604	-0.217	0.193
Tmem115	-0.901	-0.901	-0.901	1.155	0.648	0.901
Txnrd1	0.858	0.966	0.912	-0.938	-0.887	-0.912
Psmb1	0.208	1.458	0.833	-0.999	-0.667	-0.833
Raver1	1.067	0.749	0.908	-0.924	-0.892	-0.908
Rab3gap1	0.128	1.483	0.805	-1.115	-0.495	-0.805
Arhgap1	0.919	0.907	0.913	-0.913	-0.913	-0.913
Rnps1	0.792	1.026	0.909	-1.007	-0.810	-0.909
RO60	0.901	0.924	0.913	-0.946	-0.879	-0.913
Sirt2	1.510	0.120	0.815	-0.966	-0.665	-0.815

Stx4	0.686	1.122	0.904	-0.898	-0.910	-0.904
Sypl1	-0.096	1.626	0.765	-0.802	-0.729	-0.765
Tubal3	0.912	0.912	0.912	-0.975	-0.849	-0.912
Tubb6	-0.765	-0.797	-0.781	-0.036	1.599	0.781
Ubqln1	0.798	1.023	0.911	-0.922	-0.899	-0.911
Pgam2	0.968	0.857	0.912	-0.902	-0.922	-0.912
Rps27	0.521	1.254	0.887	-0.944	-0.831	-0.887
Sf3b1	0.856	0.968	0.912	-0.946	-0.878	-0.912
Ftsj3	0.216	1.007	0.611	-1.717	0.495	-0.611
Serpina11	1.019	0.791	0.905	-1.075	-0.735	-0.905
Napg	0.876	0.948	0.912	-0.949	-0.876	-0.912
Sh3bgrl	0.711	1.101	0.906	-0.906	-0.906	-0.906
Stam2	1.001	0.821	0.911	-0.971	-0.850	-0.911
Steap3	1.033	0.776	0.905	-1.071	-0.739	-0.905
Trappc5	1.120	0.623	0.872	-1.270	-0.474	-0.872
Trmt112	0.558	1.225	0.892	-0.944	-0.840	-0.892
Ufm1	0.959	0.866	0.912	-0.909	-0.916	-0.912
Rab27a	0.764	1.053	0.908	-0.978	-0.838	-0.908
Rhog	0.835	0.989	0.912	-0.905	-0.918	-0.912
Rpl23a	0.724	1.089	0.907	-0.911	-0.902	-0.907
Pop4	0.667	1.138	0.903	-0.925	-0.881	-0.903
Serpinh1	1.017	0.805	0.911	-0.910	-0.912	-0.911
Ahcyl1	0.637	1.157	0.897	-1.028	-0.766	-0.897
Scaf4	0.943	0.882	0.912	-0.878	-0.947	-0.912
Sik3	-1.230	-0.488	-0.859	0.472	1.246	0.859
Napa	0.518	1.257	0.887	-0.925	-0.849	-0.887
Sod1	0.819	1.003	0.911	-0.926	-0.897	-0.911
Rbpj	0.356	1.370	0.863	-0.961	-0.765	-0.863
Plbd2	0.752	-0.148	0.302	-1.725	1.121	-0.302
Psma4	0.917	0.909	0.913	-0.916	-0.910	-0.913
Stx16	1.818	-0.783	0.517	-0.436	-0.599	-0.517
Rps25	0.682	1.126	0.904	-0.915	-0.892	-0.904
Ruvbl2	0.710	1.096	0.903	-1.028	-0.779	-0.903
Snrpe	0.905	0.921	0.913	-0.892	-0.934	-0.913
Rtn3	0.730	1.084	0.907	-0.912	-0.903	-0.907
Slc20a1	-0.854	-0.800	-0.827	0.157	1.496	0.827
Sdha	0.959	0.865	0.912	-0.953	-0.872	-0.912
Sf3a1	0.832	0.992	0.912	-0.912	-0.911	-0.912
Srp9	0.852	0.969	0.911	-0.816	-1.005	-0.911
Vamp3	1.120	0.682	0.901	-0.779	-1.024	-0.901
Nucks1	0.634	1.149	0.892	-1.112	-0.671	-0.892
Pcbp1	0.816	1.006	0.911	-0.947	-0.875	-0.911
Psmc4	0.788	1.032	0.910	-0.881	-0.939	-0.910
Hnrnpa1	0.770	1.048	0.909	-0.930	-0.889	-0.909

Polr2h	0.727	1.086	0.906	-0.960	-0.853	-0.906
Dis3	-0.922	-0.903	-0.913	0.941	0.884	0.913
Rps27a	0.789	1.031	0.910	-0.925	-0.896	-0.910
Ahcy	0.673	1.133	0.903	-0.857	-0.948	-0.903
Snx5	0.719	1.093	0.906	-0.960	-0.852	-0.906
Supt6h	0.932	0.886	0.909	-0.775	-1.044	-0.909
Sephs1	0.897	0.897	0.897	-0.599	-1.194	-0.897
Ube4b	-1.471	0.903	-0.284	-0.637	1.205	0.284
Pol	0.791	1.030	0.910	-0.912	-0.909	-0.910
Plekho2	0.700	1.110	0.905	-0.889	-0.922	-0.905
Rabac1	0.824	0.999	0.911	-0.911	-0.911	-0.911
Cryz	0.851	0.973	0.912	-0.915	-0.909	-0.912
Rbm25	-1.271	-0.447	-0.859	0.518	1.200	0.859
Rab9a	-0.970	-0.842	-0.906	0.729	1.084	0.906
Renbp	0.810	1.011	0.911	-0.877	-0.945	-0.911
Rragc	0.529	1.229	0.879	-1.122	-0.636	-0.879
Qars1	0.778	1.040	0.909	-0.848	-0.970	-0.909
Taok1	1.715	-0.492	0.611	-1.014	-0.209	-0.611
Tbc1d23	0.601	0.637	0.619	-1.781	0.543	-0.619
Ubr5	1.288	0.474	0.881	-0.957	-0.805	-0.881
Rps2	0.551	1.226	0.889	-1.020	-0.757	-0.889
Snrpf	-0.800	-0.824	-0.812	0.090	1.534	0.812
Slc27a1	-1.681	1.433	-0.124	-0.047	0.295	0.124
S100a6	0.751	1.066	0.908	-0.919	-0.898	-0.908
Slc22a1	0.950	0.875	0.912	-0.867	-0.958	-0.912
Slc4a7	0.916	0.910	0.913	-0.932	-0.893	-0.913
Shtn1	1.636	-0.116	0.760	-0.760	-0.760	-0.760
Smc3	0.892	0.934	0.913	-0.913	-0.913	-0.913
Acaa1a	0.750	1.066	0.908	-0.950	-0.866	-0.908
Tpp2	0.804	1.017	0.911	-0.933	-0.889	-0.911
Tnfrsf26	0.736	1.067	0.902	-0.720	-1.084	-0.902
Ptp4a2	0.592	1.198	0.895	-0.966	-0.824	-0.895
Atp6v0c	1.195	0.594	0.895	-0.805	-0.985	-0.895
Wdr89	-0.846	-0.930	-0.888	0.525	1.251	0.888
Wdr48	0.783	1.037	0.910	-0.894	-0.926	-0.910
Serpine1	-0.212	1.675	0.731	-0.802	-0.661	-0.731
Pfkm	-0.966	-0.844	-0.905	0.707	1.103	0.905
Phb2	0.896	0.929	0.913	-0.913	-0.913	-0.913
Ppp1r8	1.825	-0.883	0.471	-0.510	-0.432	-0.471
Psmd5	0.714	1.097	0.906	-0.941	-0.871	-0.906
Rac1	0.777	1.042	0.910	-0.937	-0.882	-0.910
Ranbp3	0.963	0.854	0.909	-1.046	-0.772	-0.909
Rab10	0.787	1.033	0.910	-0.937	-0.883	-0.910
Rab35	0.922	0.904	0.913	-0.935	-0.890	-0.913

Rab14	0.699	1.110	0.904	-0.967	-0.842	-0.904
Rpl5	0.565	1.218	0.891	-0.992	-0.791	-0.891
Rpe	0.916	0.910	0.913	-0.913	-0.913	-0.913
Polr2a	0.377	1.163	0.770	-1.523	-0.017	-0.770
Rpp14	-0.397	-1.164	-0.780	0.055	1.506	0.780
Stap1	-0.541	0.218	-0.161	1.671	-1.348	0.161
Tbl3	0.651	1.119	0.885	-1.197	-0.573	-0.885
Trpv2	0.696	1.114	0.905	-0.916	-0.893	-0.905
Pum3	0.972	0.852	0.912	-0.929	-0.895	-0.912
Qsox2	-0.224	-1.408	-0.816	0.424	1.207	0.816
Rab2a	0.906	0.920	0.913	-0.881	-0.944	-0.913
Rab1b	0.914	0.911	0.913	-0.913	-0.913	-0.913
Rnh1	1.007	0.740	0.874	-0.436	-1.312	-0.874
Rnf114	0.823	0.998	0.911	-0.978	-0.844	-0.911
Stom	0.854	0.969	0.911	-0.981	-0.842	-0.911
Stxbp5	0.795	1.013	0.904	-1.096	-0.712	-0.904
Srpk2	-1.051	-0.665	-0.858	1.364	0.351	0.858
Srp68	0.803	1.017	0.910	-0.976	-0.844	-0.910
Mpst	0.570	1.199	0.884	-1.118	-0.650	-0.884
Tns3	1.339	-1.731	-0.196	0.029	0.363	0.196
Pgk1	0.799	1.022	0.911	-0.924	-0.897	-0.911
Raet1a	0.576	1.211	0.894	-0.933	-0.855	-0.894
Rpl7a	0.734	1.080	0.907	-0.947	-0.867	-0.907
Hnrnpab	0.934	0.888	0.911	-1.019	-0.802	-0.911
Sec23ip	-1.259	-0.468	-0.863	0.536	1.191	0.863
Serinc3	-0.841	-0.916	-0.878	1.309	0.448	0.878
Soat1	-0.902	-0.893	-0.897	0.606	1.189	0.897
Spns1	-0.907	-0.905	-0.906	0.710	1.102	0.906
Slc9a3r1	-0.947	-0.875	-0.911	0.811	1.011	0.911
Nucb2	-0.863	-0.957	-0.910	1.036	0.783	0.910
Pea15	-0.429	-0.899	-0.664	1.723	-0.394	0.664
Poglut2	-0.949	-0.867	-0.908	0.751	1.065	0.908
Ruvbl1	-1.718	0.408	-0.655	0.370	0.941	0.655
Snx27	0.816	1.004	0.910	-0.993	-0.827	-0.910
Snx3	-0.117	1.608	0.746	-1.043	-0.448	-0.746
St3gal6	-0.933	-0.885	-0.909	1.047	0.772	0.909
Sgpl1	-1.268	-0.464	-0.866	0.568	1.164	0.866
Trmt2a	-0.524	-1.242	-0.883	1.060	0.706	0.883
Spr	-0.934	-0.874	-0.904	0.681	1.126	0.904
NARS1	0.480	1.239	0.859	-1.234	-0.485	-0.859
Tubb2a	-0.876	-0.947	-0.911	0.993	0.830	0.911
Trex1	-0.891	-0.890	-0.890	1.239	0.542	0.890
Twf1	0.022	0.355	0.189	-1.727	1.350	-0.189
Ttyh3	-0.925	-0.901	-0.913	0.947	0.878	0.913

Prpf31	-0.906	-0.906	-0.906	1.105	0.707	0.906
Psma5	-1.337	-0.398	-0.867	0.714	1.021	0.867
Ptpn22	-1.124	0.018	-0.553	-0.568	1.674	0.553
Ptpa	-0.911	-0.915	-0.913	0.906	0.919	0.913
Psmd1	-1.530	0.478	-0.526	-0.287	1.340	0.526
Scpep1	-1.633	0.747	-0.443	1.147	-0.262	0.443
Rpl26	-0.918	-0.895	-0.906	0.714	1.098	0.906
Snrpg	-0.903	-0.911	-0.907	0.733	1.081	0.907
Spart	-0.367	-0.941	-0.654	-0.412	1.719	0.654
Nans	-1.133	-0.673	-0.903	0.866	0.939	0.903
Snrpd2	-0.934	-0.883	-0.909	0.756	1.061	0.909
Srbd1	-0.964	-0.856	-0.910	0.796	1.024	0.910
Sms	-0.790	-0.604	-0.697	-0.319	1.714	0.697
Srsf7	-0.306	-1.031	-0.669	-0.345	1.682	0.669
Thoc6	-0.893	-0.898	-0.896	0.589	1.202	0.896
Tnip1	-1.111	-0.687	-0.899	1.077	0.720	0.899
Tmed4	0.833	0.985	0.909	-1.036	-0.781	-0.909
Atp6ap1	-0.904	-0.904	-0.904	0.682	1.126	0.904
Wdr61	-0.779	-1.030	-0.904	0.726	1.082	0.904
Prpf40a	-0.931	-0.654	-0.792	0.019	1.565	0.792
Psip1	-0.922	-0.898	-0.910	0.784	1.036	0.910
Cad	-0.956	-0.852	-0.904	0.697	1.112	0.904
Prxl2a	-0.912	-0.896	-0.904	1.119	0.690	0.904
Rabl6	-0.931	-0.895	-0.913	0.886	0.939	0.913
Strn	0.755	0.959	0.857	-1.391	-0.323	-0.857
Umps	-1.026	-0.789	-0.908	0.790	1.025	0.908
Ube2d3	-1.151	-0.635	-0.893	0.695	1.092	0.893
Ube2g2	-1.167	-0.631	-0.899	0.830	0.968	0.899
Uchl5	-0.946	-0.875	-0.910	0.796	1.024	0.910
Txnl1	-1.697	0.265	-0.716	0.697	0.735	0.716
Ubap2l	-0.839	-0.858	-0.848	0.264	1.433	0.848
Atp6v1f	-0.909	-0.909	-0.909	0.768	1.050	0.909
Wdr75	-1.367	-0.304	-0.836	1.186	0.485	0.836
Vps36	-0.912	-0.906	-0.909	0.766	1.052	0.909
Pgk2	0.809	1.013	0.911	-0.941	-0.880	-0.911
Rnase4	-1.068	-0.725	-0.897	0.653	1.140	0.897
Rps13	-0.734	-0.976	-0.855	1.396	0.314	0.855
Sptbn1	-1.629	0.739	-0.445	-0.266	1.156	0.445
Stx12	-0.974	-0.847	-0.911	0.818	1.004	0.911
Srsf2	-0.902	-0.893	-0.897	0.606	1.189	0.897
Syne1	-0.976	-0.814	-0.895	0.592	1.198	0.895
Tubb5	-0.948	-0.876	-0.912	0.853	0.971	0.912
Trmt1	-0.905	-0.909	-0.907	0.723	1.090	0.907
Tnf	-0.941	-0.883	-0.912	0.856	0.968	0.912

Top2b	-0.888	-0.936	-0.912	0.852	0.972	0.912
Ulk1	0.889	0.937	0.913	-0.913	-0.913	-0.913
Mlkl	0.977	0.847	0.912	-0.923	-0.901	-0.912
Pmpca	1.582	-0.505	0.539	0.196	-1.274	-0.539
Nudcd1	-0.989	-0.827	-0.908	0.763	1.053	0.908
Pik3r1	0.407	0.965	0.686	0.320	-1.691	-0.686
P4ha1	-0.945	-0.871	-0.908	0.743	1.072	0.908
Prpf6	0.854	0.970	0.912	-0.939	-0.885	-0.912
Plec	-1.064	-0.746	-0.905	0.765	1.045	0.905
Slc39a6	-0.909	-0.908	-0.908	0.752	1.065	0.908
Slc5a3	-0.920	-0.905	-0.913	0.948	0.877	0.913
Scamp3	-0.918	-0.875	-0.897	0.599	1.194	0.897
Snd1	0.036	1.461	0.748	-1.307	-0.190	-0.748
Sh3bgrl3	-0.932	-0.892	-0.912	0.853	0.971	0.912
Trnt1	-0.759	-1.055	-0.907	0.997	0.818	0.907
Pxn	-0.961	-0.774	-0.867	1.351	0.384	0.867
Pfn1	-0.931	-0.871	-0.901	0.650	1.152	0.901
Prdx6	-0.920	-0.897	-0.909	0.759	1.058	0.909
Pter	-0.893	-0.915	-0.904	0.686	1.123	0.904
Pfas	-0.945	-0.088	-0.517	1.748	-0.715	0.517
Puf60	0.820	1.003	0.911	-0.899	-0.924	-0.911
Pum2	-0.950	-0.871	-0.911	0.806	1.015	0.911
Rpl15	-0.947	-0.859	-0.903	0.673	1.133	0.903
Rpl31	-0.957	-0.828	-0.893	0.567	1.218	0.893
Snx1	-1.166	-0.422	-0.794	0.108	1.480	0.794
Slco4a1	-0.932	-0.893	-0.913	0.941	0.884	0.913
Stxbp1	-0.886	-0.937	-0.912	0.837	0.987	0.912
Psma7	-0.916	-0.885	-0.901	0.643	1.158	0.901
Psma3	-0.942	-0.883	-0.912	0.948	0.877	0.912
Rnf7	-0.749	-0.974	-0.862	0.352	1.372	0.862
Rab34	-0.922	-0.900	-0.911	0.808	1.014	0.911
Rpl18a	0.646	1.154	0.900	-0.984	-0.816	-0.900
Rrm2	0.918	0.908	0.913	-0.913	-0.913	-0.913
Rplp2	-0.953	-0.867	-0.910	0.789	1.031	0.910
Polr3c	-0.591	-1.195	-0.893	1.023	0.763	0.893
Vti1b	-0.923	-0.901	-0.912	0.847	0.977	0.912
Wipf1	-1.068	-0.748	-0.908	0.960	0.855	0.908
Xpot	-0.807	-0.307	-0.557	-0.670	1.784	0.557
Nle1	-0.919	-0.900	-0.909	0.773	1.046	0.909
Psmd6	-0.870	-0.634	-0.752	-0.138	1.641	0.752
Rpl18	-0.918	-0.904	-0.911	0.810	1.012	0.911
Slc6a12	-0.921	-0.904	-0.912	0.846	0.978	0.912
Uba2	-1.460	-0.099	-0.780	1.242	0.317	0.780
Psat1	-0.909	-0.912	-0.910	0.791	1.029	0.910

Shc1	-1.167	-0.576	-0.871	0.505	1.238	0.871
Srgn	-0.959	-0.861	-0.910	1.022	0.799	0.910
Serbp1	-1.079	-0.716	-0.898	0.673	1.122	0.898
Oxsr1	-0.833	-0.963	-0.898	0.622	1.174	0.898
Pwp1	0.183	1.388	0.786	-1.320	-0.251	-0.786
Racgap1	-0.947	-0.875	-0.911	1.005	0.817	0.911
Rpl12	-0.989	-0.827	-0.908	0.768	1.048	0.908
Rpl36	-0.960	-0.807	-0.883	0.492	1.274	0.883
Rock1	-1.036	-0.772	-0.904	0.728	1.080	0.904
Rnf149	-0.373	-1.310	-0.841	1.239	0.444	0.841
Rpn1	-0.928	-0.897	-0.913	0.884	0.941	0.913
Rnf213	-1.747	0.676	-0.535	0.119	0.951	0.535
Rpf2	-0.761	1.775	0.507	-0.854	-0.160	-0.507
Sqstm1	0.920	0.898	0.909	-0.768	-1.050	-0.909
Sdf2l1	-0.911	-0.911	-0.911	1.011	0.811	0.911
Nckap1l	-0.996	-0.804	-0.900	0.652	1.148	0.900
Myo9b	0.245	0.502	0.374	1.063	-1.811	-0.374
Myo1g	-0.910	-0.901	-0.905	0.703	1.107	0.905
Snu13	-0.904	-0.889	-0.896	0.598	1.195	0.896
Nhlrc2	-0.916	-0.904	-0.910	0.790	1.030	0.910
Nemf	-0.982	-0.825	-0.903	0.687	1.119	0.903
Ndufv1	-0.919	-0.906	-0.912	0.855	0.970	0.912
Nol6	-0.372	-0.526	-0.449	1.824	-0.925	0.449
Pdcd6ip	-0.914	-0.907	-0.910	0.791	1.029	0.910
Sec22b	-0.904	-0.921	-0.912	0.959	0.866	0.912
Ahcyl2	-0.938	-0.885	-0.912	0.829	0.994	0.912
Smu1	-1.007	-0.785	-0.896	0.614	1.178	0.896
Slc2a6	-0.952	-0.873	-0.913	0.904	0.921	0.913
Pex14	-0.959	-0.866	-0.912	0.912	0.912	0.912
Plrg1	-0.896	-0.927	-0.912	0.835	0.988	0.912
Pi4ka	-1.047	-0.720	-0.883	1.248	0.519	0.883
Rpl10	-0.906	-0.891	-0.898	0.617	1.180	0.898
Rpl13	-0.982	-0.814	-0.898	0.627	1.169	0.898
Rsl1d1	0.297	-1.441	-0.572	-0.302	1.446	0.572
Hnrnpa0	-0.787	-1.033	-0.910	0.864	0.956	0.910
Polr2j	1.627	-0.448	0.590	0.027	-1.206	-0.590
Polr3e	-0.899	-0.923	-0.911	1.019	0.802	0.911
Rrbp1	-1.037	-0.764	-0.900	0.675	1.126	0.900
Rps11	0.633	1.084	0.859	-1.345	-0.372	-0.859
Slc38a1	-1.046	-0.754	-0.900	0.679	1.121	0.900
Slc12a6	-0.942	-0.882	-0.912	0.864	0.960	0.912
Sec13	-0.942	-0.867	-0.905	0.695	1.114	0.905
Slc6a9	0.114	0.239	0.177	-1.727	1.373	-0.177
Selenof	-0.936	-0.887	-0.912	0.829	0.994	0.912

Usp39	-0.961	-0.857	-0.909	0.769	1.049	0.909
Snrnp40	-1.024	-0.781	-0.903	0.702	1.104	0.903
Ndrg1	-1.092	-0.695	-0.894	0.639	1.148	0.894
Nop56	-1.014	-0.800	-0.907	0.758	1.055	0.907
Nubp1	0.500	1.230	0.865	-1.214	-0.517	-0.865
Numa1	-1.005	-0.794	-0.899	0.647	1.151	0.899
Numb	-0.895	-0.911	-0.903	1.132	0.675	0.903
Osbpl9	-1.110	-0.691	-0.900	1.056	0.744	0.900
Pan3	-0.902	-0.924	-0.913	0.913	0.913	0.913
Slc39a14	-0.906	-0.920	-0.913	0.900	0.926	0.913
Scrn2	-0.981	-0.838	-0.910	1.019	0.801	0.910
Sbds	0.405	1.264	0.834	-1.310	-0.359	-0.834
Sfpq	-0.989	-0.705	-0.847	0.274	1.420	0.847
Psmd8	-0.897	1.187	0.145	-1.308	1.017	-0.145
Rbm39	-1.382	-0.322	-0.852	1.054	0.650	0.852
Ranbp2	-1.146	-0.634	-0.890	1.129	0.651	0.890
Rap1a	-0.920	-0.865	-0.893	0.564	1.221	0.893
Arhgap17	-1.339	0.755	-0.292	-0.779	1.363	0.292
Reep5	-0.930	-0.883	-0.906	0.721	1.092	0.906
Rpl14	-0.917	-0.902	-0.909	0.772	1.047	0.909
Rps21	-0.822	1.044	0.111	-1.373	1.151	-0.111
Rps7	-0.950	-0.865	-0.908	0.743	1.073	0.908
Slc26a2	-1.100	-0.692	-0.896	0.673	1.119	0.896
Snrpa1	-0.913	-0.904	-0.908	0.754	1.063	0.908
Slc15a3	0.978	0.846	0.912	-0.914	-0.910	-0.912
Pdlim5	-0.942	-0.880	-0.911	0.819	1.003	0.911
Pdxdc1	-1.295	-0.419	-0.857	0.531	1.182	0.857
Pip5k1a	-0.906	-0.906	-0.906	0.706	1.105	0.906
Plxnb2	-1.011	-0.809	-0.910	0.835	0.985	0.910
Ppt2	-0.968	-0.840	-0.904	1.118	0.690	0.904
Psmc6	-1.474	0.193	-0.641	1.398	-0.117	0.641
Plcg2	0.561	1.223	0.892	-0.833	-0.951	-0.892
Arl6ip5	-0.842	-0.982	-0.912	0.898	0.926	0.912
Ppig	0.845	0.979	0.912	-0.928	-0.896	-0.912
Prdx4	-1.051	-0.764	-0.908	0.816	0.999	0.908
Pus10	-0.873	-0.946	-0.909	0.777	1.042	0.909
Rad21	-1.041	-0.777	-0.909	0.841	0.977	0.909
Rabgap1	0.459	1.256	0.858	-0.492	-1.224	-0.858
Riox2	-0.964	-0.856	-0.910	0.794	1.026	0.910
Rftn1	-0.926	-0.847	-0.886	0.509	1.263	0.886
Rps12	-1.036	0.856	-0.090	-1.168	1.347	0.090
Stat3	-1.017	-0.737	-0.877	0.461	1.293	0.877
Rtn4	-0.934	-0.878	-0.906	0.711	1.100	0.906
Slc39a7	-0.770	-0.770	-0.770	-0.080	1.620	0.770

Pnkp	-0.839	-0.927	-0.883	0.484	1.282	0.883
Ppp1r18	-0.921	-0.895	-0.908	0.751	1.066	0.908
Pygb	-0.931	-0.893	-0.912	0.861	0.964	0.912
Rab6a	-0.928	-0.886	-0.907	0.731	1.084	0.907
Rap1b	-0.854	-0.956	-0.905	0.707	1.104	0.905
Raet1c	-0.906	-0.906	-0.906	0.710	1.101	0.906
Rab32	-0.913	-0.911	-0.912	0.857	0.968	0.912
Rpl24	-0.998	-0.815	-0.906	0.743	1.070	0.906
Rras	-1.024	-0.783	-0.904	1.091	0.716	0.904
Rraga	-0.767	-0.826	-0.797	0.026	1.568	0.797
Ppp4c	-1.251	-0.526	-0.889	0.891	0.886	0.889
Rab7a	-0.892	-0.787	-0.839	0.220	1.459	0.839
Rps3	-0.970	-0.836	-0.903	0.684	1.123	0.903
Srrm1	0.782	1.038	0.910	-0.910	-0.910	-0.910
U2surp	-0.913	-0.913	-0.913	0.904	0.921	0.913
Srrm2	-0.872	-0.925	-0.899	0.621	1.176	0.899
Rps5	0.730	1.020	0.875	-1.301	-0.449	-0.875
Rps3a	-0.922	-0.902	-0.912	0.835	0.989	0.912
Nolc1	0.366	1.324	0.845	-0.488	-1.203	-0.845
Nutf2	-1.000	-0.791	-0.895	0.603	1.187	0.895
Ocrl	-0.519	-0.389	-0.454	1.824	-0.915	0.454
Pdxk	-0.881	-0.937	-0.909	0.775	1.044	0.909
Ppia	-0.920	-0.900	-0.910	0.787	1.033	0.910
Rasa3	-0.990	-0.833	-0.912	0.929	0.894	0.912
Ran	-0.928	-0.895	-0.912	0.840	0.984	0.912
Rab12	0.731	1.083	0.907	-0.926	-0.888	-0.907
Rab21	-0.993	-0.814	-0.903	0.693	1.114	0.903
Rps23	-0.880	-0.892	-0.886	0.507	1.266	0.886
Arhgap45	-0.955	-0.869	-0.912	0.840	0.983	0.912
Hmgcl	-1.051	-0.703	-0.877	0.476	1.279	0.877
Ifi44	-0.227	1.580	0.676	-1.235	-0.118	-0.676
Ide	-0.925	-0.887	-0.906	0.717	1.095	0.906
Iqgap1	-0.921	-0.904	-0.912	0.863	0.962	0.912
Prkar2a	-0.900	-0.924	-0.912	0.863	0.962	0.912
Mtx1	-1.393	-0.312	-0.852	0.682	1.023	0.852
Pofut2	-0.925	-0.891	-0.908	0.749	1.067	0.908
Pcbp2	-0.935	-0.876	-0.906	0.712	1.100	0.906
Pebp1	-0.948	-0.877	-0.913	0.905	0.920	0.913
Pdxp	-0.908	-0.908	-0.908	0.744	1.072	0.908
Pld3	-0.920	-0.852	-0.886	0.507	1.265	0.886
Pkn1	-0.806	-1.009	-0.908	0.774	1.042	0.908
Helz2	-0.912	-0.912	-0.912	0.984	0.840	0.912
Kif5b	-1.173	-0.617	-0.895	1.034	0.756	0.895
Prpsap2	-0.910	-0.892	-0.901	0.645	1.156	0.901

Slc25a3	-0.995	-0.828	-0.911	0.868	0.954	0.911
Msn	-0.908	-0.912	-0.910	0.778	1.041	0.910
Adi1	-0.895	-0.913	-0.904	0.679	1.128	0.904
Naa50	-0.931	-0.893	-0.912	0.846	0.978	0.912
Pals2	-1.106	-0.612	-0.859	1.333	0.386	0.859
Hprt1	-0.931	-0.886	-0.908	0.749	1.067	0.908
Hs2st1	-0.589	-1.074	-0.831	1.438	0.225	0.831
Hnrnpu	-1.124	-0.659	-0.892	0.644	1.139	0.892
Hk3	0.244	1.421	0.832	-1.108	-0.556	-0.832
Ireb2	-0.920	-0.904	-0.912	0.994	0.829	0.912
Isoc1	0.877	-0.156	0.360	-1.718	0.997	-0.360
Itgb5	-0.840	-0.906	-0.873	0.412	1.334	0.873
Rps6ka1	-1.117	-0.687	-0.902	0.785	1.018	0.902
Nucb1	-0.877	-0.640	-0.758	-0.114	1.630	0.758
Pip4k2a	-0.949	-0.870	-0.910	0.783	1.036	0.910
Myl6	-0.913	-0.891	-0.902	0.661	1.143	0.902
Myh14	0.856	0.968	0.912	-0.918	-0.906	-0.912
Myo1d	-0.952	-0.859	-0.906	0.715	1.096	0.906
Nif3l1	-0.585	-0.400	-0.492	-0.836	1.821	0.492
Nmral1	-0.898	-0.868	-0.883	0.482	1.284	0.883
Nrxn1	0.862	0.962	0.912	-0.904	-0.921	-0.912
Os9	0.385	1.301	0.843	-0.445	-1.241	-0.843
P2rx7	-0.942	-0.877	-0.909	0.776	1.043	0.909
Ppm1f	-0.920	-0.896	-0.908	1.072	0.744	0.908
Hikeshi	-0.798	-0.798	-0.798	1.565	0.032	0.798
Hdac2	-0.911	-0.898	-0.904	0.689	1.119	0.904
Hspa14	-1.699	0.272	-0.714	0.688	0.739	0.714
Hspbp1	0.786	1.034	0.910	-0.913	-0.907	-0.910
Hspa1a	-1.276	-0.478	-0.877	1.055	0.700	0.877
Eif4a1	-0.948	-0.867	-0.907	0.741	1.074	0.907
Itm2b	-1.302	-0.418	-0.860	0.569	1.152	0.860
Mki67	-0.970	-0.847	-0.908	1.051	0.765	0.908
Mapk14	-0.922	-0.893	-0.907	0.730	1.084	0.907
Mrto4	-0.909	-0.911	-0.910	0.778	1.041	0.910
Pabpc1	-0.918	-0.907	-0.912	0.858	0.967	0.912
Hspg2	0.835	0.989	0.912	-0.912	-0.912	-0.912
Itga4	-0.945	-0.880	-0.913	0.917	0.909	0.913
H3c1	-0.911	-0.906	-0.908	0.752	1.065	0.908
Hpn	0.869	0.956	0.912	-0.949	-0.876	-0.912
Camk2d	-0.897	-0.840	-0.868	0.382	1.355	0.868
Mtrex	-0.915	-0.898	-0.907	0.726	1.088	0.907
Mthfr	0.338	1.384	0.861	-0.824	-0.898	-0.861
Nampt	-0.931	-0.895	-0.913	0.889	0.937	0.913
Ncl	-0.931	-0.894	-0.912	0.867	0.958	0.912

Dbt	0.384	0.938	0.661	0.395	-1.716	-0.661
Pcyox1l	-0.901	-0.901	-0.901	0.650	1.152	0.901
Pacs1	-1.036	-0.658	-0.847	0.289	1.405	0.847
Ppp6r3	-0.866	-0.953	-0.909	0.772	1.046	0.909
Ppwd1	-1.004	-0.687	-0.846	0.271	1.420	0.846
Hnrnpll	-1.451	-0.234	-0.842	0.877	0.808	0.842
Ccl18	-0.913	-0.913	-0.913	0.930	0.896	0.913
Inf2	0.398	1.267	0.832	-1.315	-0.350	-0.832
Itga4	-0.913	-0.906	-0.909	0.774	1.045	0.909
Kpna1	-0.623	0.425	-0.099	-1.383	1.581	0.099
Itpa	-0.945	-0.864	-0.905	0.697	1.113	0.905
Lpxn	-1.373	-0.353	-0.863	0.800	0.927	0.863
Lrrc40	-0.908	-0.900	-0.904	0.679	1.128	0.904
Man2c1	-0.913	-0.908	-0.910	0.787	1.033	0.910
Milr1	-0.911	-0.911	-0.911	0.823	1.000	0.911
Pofut1	-0.202	0.317	0.057	1.499	-1.614	-0.057
Lrrc25	-0.891	-0.922	-0.906	0.719	1.094	0.906
Lamtor1	-0.907	-0.907	-0.907	0.725	1.088	0.907
Lyz2	1.095	0.507	0.801	-0.101	-1.501	-0.801
Nedd8	-0.910	-0.910	-0.910	0.795	1.026	0.910
Matr3	-0.892	-0.912	-0.902	0.660	1.144	0.902
Sell	0.560	1.179	0.870	-1.238	-0.501	-0.870
Nploc4	-0.969	-0.826	-0.898	0.619	1.176	0.898
Prcp	-0.707	-0.947	-0.827	1.486	0.168	0.827
P4hb	-0.939	-0.876	-0.907	0.739	1.076	0.907
Pdia6	-0.910	-0.915	-0.912	0.848	0.976	0.912
Pgam5	-0.992	-0.828	-0.910	0.824	0.996	0.910
Ltv1	-0.911	-0.911	-0.911	0.811	1.011	0.911
Mon2	-1.048	-0.713	-0.881	1.261	0.500	0.881
Myo1e	-0.979	-0.824	-0.902	0.668	1.136	0.902
Oas1a	0.402	0.798	0.600	-1.775	0.574	-0.600
Parp4	-0.915	-0.908	-0.911	0.820	1.003	0.911
Gsr	-0.909	-0.916	-0.912	0.865	0.960	0.912
Mdc1	-1.361	-0.373	-0.867	0.867	0.867	0.867
Wdr77	-1.070	-0.271	-0.670	-0.325	1.666	0.670
Msr1	-0.925	-0.885	-0.905	0.699	1.111	0.905
Myo10	-0.906	-0.906	-0.906	1.099	0.713	0.906
Ncstn	-0.951	-0.863	-0.907	0.731	1.082	0.907
Pfdn6	-0.794	-1.026	-0.910	0.871	0.949	0.910
Pdcl3	-0.978	-0.781	-0.879	1.292	0.466	0.879
Pak1	-1.569	0.052	-0.759	0.419	1.099	0.759
Pds5a	-1.076	-0.739	-0.907	0.871	0.944	0.907
Rps6ka3	-1.070	-0.661	-0.865	0.404	1.326	0.865
Spn	-0.894	-0.921	-0.908	0.743	1.073	0.908

Mcm3	-0.978	-0.758	-0.868	0.392	1.344	0.868
Nop58	-0.977	-0.841	-0.909	0.784	1.035	0.909
Nme1	-0.904	-0.918	-0.911	0.815	1.007	0.911
Npl	-1.022	-0.763	-0.893	0.588	1.197	0.893
Lypla1	0.607	1.185	0.896	-0.986	-0.806	-0.896
Metap1	0.569	0.971	0.770	-1.596	0.056	-0.770
Nt5c	0.804	1.013	0.908	-1.023	-0.794	-0.908
Mix23	-0.893	-0.922	-0.908	0.738	1.077	0.908
Mpi	-0.874	-0.874	-0.874	0.420	1.329	0.874
Mob1b	-0.965	-0.859	-0.912	0.868	0.956	0.912
Morf4l1	-0.875	-0.875	-0.875	1.328	0.421	0.875
Mta2	-0.934	-0.886	-0.910	0.787	1.033	0.910
Oga	-0.905	-0.920	-0.912	0.863	0.962	0.912
Papss1	-0.970	-0.845	-0.908	0.753	1.063	0.908
Parp10	-1.251	-0.527	-0.889	0.889	0.889	0.889
Nfkb1	-0.925	-0.900	-0.913	0.873	0.952	0.913
Rps16	-0.957	-0.831	-0.894	0.578	1.210	0.894
Rps9	-0.922	-0.901	-0.911	0.825	0.998	0.911
Srpra	-0.948	-0.875	-0.912	0.837	0.986	0.912
Nln	-0.817	-0.982	-0.900	0.646	1.154	0.900
Niban2	-0.890	-0.928	-0.909	0.771	1.048	0.909
Nek9	-1.407	0.360	-0.523	-0.424	1.471	0.523
Oard1	-0.944	-0.864	-0.904	0.691	1.118	0.904
Pla2g4a	-0.921	-0.148	-0.535	-0.688	1.757	0.535
Psmc3	-0.952	-0.856	-0.904	0.689	1.119	0.904
Ppie	-0.861	-0.935	-0.898	0.618	1.178	0.898
Gart	-1.076	-0.618	-0.847	0.303	1.391	0.847
Pip4k2c	1.055	0.758	0.906	-1.021	-0.792	-0.906
Plod1	0.803	1.019	0.911	-0.912	-0.909	-0.911
Pak1ip1	0.743	1.072	0.907	-0.955	-0.860	-0.907
Prpf38a	0.558	1.226	0.892	-0.924	-0.860	-0.892
Ppm1b	-0.851	-0.939	-0.895	0.588	1.202	0.895
Ptpra	-0.895	-0.929	-0.912	0.848	0.976	0.912
Rab8a	-0.926	-0.841	-0.883	0.486	1.280	0.883
Rbbp7	-1.038	-0.768	-0.903	0.720	1.087	0.903
Rae1	-0.944	-0.860	-0.902	0.663	1.141	0.902
Rfc3	-0.515	-1.163	-0.839	0.307	1.371	0.839
Hnrnpa3	-1.004	-0.792	-0.898	0.637	1.160	0.898
Elac2	-0.893	-0.862	-0.877	0.441	1.314	0.877
Rpap1	-0.863	-0.953	-0.908	0.756	1.060	0.908
Naa35	0.048	1.556	0.802	-0.757	-0.847	-0.802
Mvp	-0.921	-0.904	-0.913	0.871	0.954	0.913
Nisch	-0.919	-0.895	-0.907	1.087	0.727	0.907
Nup160	-1.010	-0.238	-0.624	-0.464	1.712	0.624

Pgrmc2	-1.012	-0.808	-0.910	0.973	0.847	0.910
Ppt1	-0.878	-0.910	-0.894	0.573	1.215	0.894
Psmb4	-1.352	-0.377	-0.865	0.724	1.005	0.865
Ptpn23	-1.657	0.188	-0.734	0.557	0.912	0.734
Rap2a	-0.907	-0.913	-0.910	0.779	1.041	0.910
Lsm4	-1.173	-0.572	-0.872	0.516	1.229	0.872
Lsm2	-0.809	-0.809	-0.809	1.542	0.075	0.809
Lrrc59	-0.893	-0.923	-0.908	1.071	0.745	0.908
Lrch4	-0.901	-0.901	-0.901	0.652	1.151	0.901
Mettl26	0.838	0.986	0.912	-0.908	-0.915	-0.912
Psmc5	-1.122	-0.670	-0.896	0.696	1.096	0.896
Prrc2c	-1.081	-0.680	-0.880	0.512	1.248	0.880
Ptpn6	-0.946	-0.879	-0.912	0.878	0.947	0.912
Psmd4	0.679	0.967	0.823	-1.492	-0.154	-0.823
Psme4	-0.879	-0.904	-0.891	0.550	1.232	0.891
Upf1	-1.007	-0.790	-0.899	0.644	1.154	0.899
Prkaca	-0.921	-0.891	-0.906	0.719	1.094	0.906
Med23	-1.197	-0.578	-0.887	1.091	0.684	0.887
Pbdc1	-0.901	-0.901	-0.901	0.650	1.153	0.901
Parp1	-1.010	-0.810	-0.910	0.989	0.831	0.910
Pfdn2	-0.948	-0.865	-0.907	0.726	1.087	0.907
Plpp1	1.542	-0.017	0.763	-1.148	-0.377	-0.763
Cfp	-0.426	-1.157	-0.792	0.095	1.488	0.792
Pttg1ip	-0.055	1.523	0.734	-0.222	-1.246	-0.734
Ptpn2	1.545	0.035	0.790	-1.031	-0.549	-0.790
Rpl17	-0.709	1.464	0.377	-1.322	0.567	-0.377
Rpl13a	-0.953	-0.864	-0.909	0.764	1.053	0.909
Rpl10a	-0.933	-0.881	-0.907	0.733	1.081	0.907
Rpl27a	-0.908	-0.872	-0.890	0.540	1.241	0.890
Nectin1	-0.880	-0.880	-0.880	0.463	1.298	0.880
Ntmt1	-0.896	-0.896	-0.896	0.592	1.200	0.896
Nrbp1	-0.817	-0.981	-0.899	1.162	0.635	0.899
Prps2	-0.951	-0.841	-0.896	0.595	1.196	0.896
Psmc1	0.809	1.012	0.911	-0.945	-0.877	-0.911
Rgn	-0.946	-0.851	-0.898	1.176	0.620	0.898
Rpl21	-0.909	-0.891	-0.900	0.636	1.164	0.900
Hsph1	-0.943	-0.870	-0.906	0.718	1.094	0.906
Prex1	0.457	-1.754	-0.648	0.520	0.776	0.648
Vbp1	-0.920	-0.906	-0.913	0.937	0.889	0.913
Npepps	1.131	0.582	0.856	-1.331	-0.381	-0.856
Psme2	-1.101	-0.698	-0.900	0.722	1.077	0.900
Rab8b	-0.809	-0.989	-0.899	0.637	1.160	0.899
Ranbp1	-1.061	-0.754	-0.907	0.985	0.830	0.907
Rasa4	-0.905	-0.905	-0.905	0.699	1.111	0.905

Rfc5	-0.910	-0.910	-0.910	1.045	0.774	0.910
Rplp0	-0.983	-0.832	-0.907	0.750	1.065	0.907
Pzp	-1.032	-0.389	-0.710	-0.229	1.650	0.710
Rdx	-0.933	-0.889	-0.911	0.812	1.010	0.911
Rrm1	-0.934	-0.891	-0.913	0.880	0.945	0.913
Rhoa	-0.938	-0.856	-0.897	0.606	1.188	0.897
Riox1	-0.925	-0.890	-0.907	0.734	1.080	0.907
Rpl28	-1.374	-0.179	-0.777	0.199	1.354	0.777
Rpl7l1	0.965	0.859	0.912	-0.885	-0.940	-0.912
Rock2	0.829	0.994	0.911	-0.942	-0.881	-0.911
Tfrc	0.827	0.996	0.912	-0.921	-0.902	-0.912
Tnfaip8	0.862	0.916	0.889	-1.248	-0.530	-0.889
Tmem214	0.840	0.984	0.912	-0.913	-0.911	-0.912
Vcpip1	0.995	0.823	0.909	-1.020	-0.799	-0.909
Stat6	0.913	0.912	0.913	-0.902	-0.924	-0.913
Srpk1	0.732	1.083	0.907	-0.907	-0.907	-0.907
Stk3	0.717	1.089	0.903	-1.040	-0.766	-0.903
Sephs2	0.820	1.002	0.911	-0.915	-0.907	-0.911
Iars1	-1.008	-0.801	-0.905	0.719	1.090	0.905
Mars1	-1.044	-0.749	-0.896	0.634	1.159	0.896
Tax1bp1	-0.913	-0.867	-0.890	1.240	0.540	0.890
Thoc5	1.017	0.777	0.897	-0.628	-1.165	-0.897
Txnip	-1.092	-0.691	-0.892	1.165	0.618	0.892
Atp6v1c1	-0.968	-0.852	-0.910	0.796	1.024	0.910
Vamp4	-1.165	-0.629	-0.897	1.019	0.775	0.897
Rp2	-1.206	-0.465	-0.835	1.354	0.316	0.835
Rpl34	-0.882	-0.941	-0.911	0.828	0.995	0.911
Srsf10	-1.260	0.937	-0.162	-0.940	1.263	0.162
Ubtd1	-0.777	-0.980	-0.879	1.295	0.462	0.879
Them6	-0.897	-0.929	-0.913	0.898	0.928	0.913
U2af2	-1.445	-0.204	-0.824	0.550	1.099	0.824
Tdp2	-0.836	-0.985	-0.910	1.004	0.816	0.910
Rps20	-1.119	1.778	0.329	-0.606	-0.053	-0.329
Rbm17	0.724	1.089	0.907	-0.899	-0.915	-0.907
Supt4h1a	-0.877	-0.936	-0.906	0.716	1.096	0.906
Strn3	-1.072	-0.727	-0.899	1.109	0.690	0.899
Trrap	-0.646	-1.155	-0.900	0.967	0.834	0.900
Tmem106a	0.681	1.126	0.903	-0.962	-0.844	-0.903
Tubg1	1.215	0.567	0.891	-1.006	-0.776	-0.891
Tmod3	0.727	1.066	0.896	-1.143	-0.650	-0.896
Tsn	1.014	0.807	0.911	-0.911	-0.911	-0.911
Usp9x	0.653	1.138	0.895	-1.085	-0.706	-0.895
Tor2a	0.841	0.980	0.910	-1.006	-0.815	-0.910
Usp19	-0.922	-0.902	-0.912	0.980	0.844	0.912

Ubxn7	-0.895	-0.895	-0.895	0.584	1.206	0.895
Ube2l3	-0.925	-0.869	-0.897	0.604	1.190	0.897
Vcl	-0.994	-0.819	-0.906	1.073	0.740	0.906
Zdhhc17	0.833	0.981	0.907	-0.748	-1.066	-0.907
Tm9sf2	-1.025	-0.771	-0.898	0.644	1.152	0.898
Tnfsf9	-0.910	-0.910	-0.910	0.790	1.031	0.910
Ube2f	-0.929	-0.890	-0.909	0.775	1.044	0.909
Vamp7	-0.950	-0.872	-0.911	0.830	0.993	0.911
Vps13a	-0.899	-0.919	-0.909	0.766	1.053	0.909
Xrn2	-1.104	-0.685	-0.894	0.657	1.132	0.894
Washc5	1.394	-1.070	0.162	0.789	-1.112	-0.162
Xpo7	-0.919	-0.906	-0.913	0.873	0.952	0.913
Xpnpep1	-0.946	-0.867	-0.906	0.720	1.093	0.906
Wnk1	-0.833	-0.964	-0.898	0.626	1.171	0.898
Vps11	0.892	0.924	0.908	-1.070	-0.746	-0.908
Wasf2	0.859	0.966	0.912	-0.906	-0.918	-0.912
Nelfcd	0.998	0.821	0.909	-1.018	-0.800	-0.909
Nsdhl	0.805	1.017	0.911	-0.895	-0.926	-0.911
Osbpl8	0.796	1.025	0.910	-0.926	-0.895	-0.910
Pepd	0.758	1.059	0.908	-0.948	-0.869	-0.908
Uggt1	-1.133	-0.648	-0.891	0.645	1.137	0.891
Tsr1	-0.931	-0.875	-0.903	0.672	1.134	0.903
Ubash3b	-0.908	-0.908	-0.908	0.746	1.070	0.908
Wdfy1	0.828	0.995	0.911	-0.882	-0.941	-0.911
Ugt1a7	-0.921	-0.904	-0.913	0.918	0.907	0.913
Txndc5	-0.934	-0.877	-0.906	1.102	0.709	0.906
Tyrobp	-0.907	-0.907	-0.907	0.730	1.084	0.907
Ube2k	0.110	1.460	0.785	-0.340	-1.229	-0.785
Rrp9	-0.942	-0.870	-0.906	0.713	1.099	0.906
Uxt	-0.814	-0.985	-0.900	1.151	0.649	0.900
Wdr91	0.932	0.892	0.912	-0.968	-0.856	-0.912
Wdr1	-0.913	-0.907	-0.910	0.781	1.039	0.910
Wdr33	-0.889	-0.920	-0.904	0.686	1.122	0.904
Slc7a6	-0.835	-0.982	-0.909	1.041	0.776	0.909
Zdhhc20	-0.657	-0.633	-0.645	-0.473	1.764	0.645
Slc30a1	0.117	-0.130	-0.007	-1.570	1.583	0.007
Vdac2	-0.914	-0.905	-0.910	0.780	1.040	0.910
Vps13c	-0.927	-0.894	-0.911	0.810	1.012	0.911
Vps26b	-0.900	-0.924	-0.912	0.836	0.987	0.912
Wipi2	-1.036	-0.743	-0.889	0.565	1.214	0.889
Wfikkn1	0.023	1.438	0.730	-1.362	-0.098	-0.730
Yipf3	-0.913	-0.909	-0.911	0.799	1.022	0.911
Wdr18	0.774	1.043	0.908	-0.995	-0.821	-0.908
Vps28	-0.937	-0.887	-0.912	0.846	0.978	0.912

Vps41	-0.608	-0.667	-0.637	1.769	-0.494	0.637
Zc3h15	-0.686	-1.122	-0.904	0.872	0.936	0.904
Pus1	0.913	0.913	0.913	-0.952	-0.873	-0.913
Ubr4	-1.281	-0.116	-0.698	1.533	-0.136	0.698
Flt1	-0.693	-0.547	-0.620	-0.538	1.778	0.620
Atp6v1g1	-0.911	-0.911	-0.911	0.799	1.022	0.911
Plscr1	0.484	1.258	0.871	-1.143	-0.600	-0.871
Usp24	-0.934	-0.876	-0.905	0.695	1.114	0.905
Vrk1	-0.937	-0.885	-0.911	0.815	1.007	0.911
Vps4a	-0.905	-0.895	-0.900	0.636	1.164	0.900
Vapa	-0.929	-0.893	-0.911	1.022	0.799	0.911
Xpo1	-0.994	-0.826	-0.910	0.814	1.006	0.910
Map2k1	-1.021	-0.800	-0.911	0.924	0.897	0.911
Ogg1	-0.449	1.756	0.653	-0.724	-0.582	-0.653
Nup37	-0.002	0.262	0.130	1.429	-1.690	-0.130
Parg	-0.916	-0.909	-0.912	0.856	0.969	0.912
Plek	0.611	1.184	0.897	-0.938	-0.857	-0.897
Ptgr1	-0.967	-0.308	-0.637	-0.445	1.720	0.637
Rab5c	-0.915	-0.907	-0.911	0.807	1.015	0.911
Rcc2	-0.942	-0.872	-0.907	0.734	1.081	0.907
Rab4b	-0.930	-0.895	-0.913	0.873	0.952	0.913
Rangap1	-0.881	-0.936	-0.909	0.765	1.053	0.909
Rhbdf2	-1.038	-0.665	-0.852	0.314	1.390	0.852
Lamp2	0.864	0.873	0.868	-1.356	-0.380	-0.868
Myo1f	-1.026	-0.795	-0.910	0.879	0.942	0.910
Ndufs1	-0.913	-0.913	-0.913	0.888	0.937	0.913
Niban1	-0.897	-0.635	-0.766	-0.084	1.616	0.766
Ogfr	-0.873	-0.910	-0.892	0.554	1.230	0.892
Pycr1	-0.870	-0.929	-0.899	0.631	1.168	0.899
Ppp4r3a	-1.139	-0.666	-0.902	0.879	0.926	0.902
Otulin	0.024	1.492	0.758	-1.247	-0.269	-0.758
Pfdn1	0.403	1.341	0.872	-0.872	-0.872	-0.872
Macroh2a1	-0.961	-0.862	-0.911	0.836	0.986	0.911
Prpsap1	-0.972	-0.835	-0.903	0.685	1.121	0.903
Tmpo	-1.713	0.366	-0.674	0.433	0.915	0.674
M6pr	-1.314	0.272	-0.521	-0.507	1.549	0.521
Mndal	-0.935	-0.878	-0.907	0.729	1.085	0.907

Table S6. List of DEP from proteomics analysis (IE vs CIE)

Gene	IE1	IE2	IE3	CIE1	CIE2	CIE3
**SOX9**	-0.904	-0.921	-0.913	0.955	0.940	0.913
TFRC	0.848	0.974	0.911	-0.850	-0.973	-0.911
TSPYL2	0.751	1.066	0.908	-0.907	-0.910	-0.908

CLIC4	-0.893	-0.932	-0.912	0.974	0.851	0.912
MARCKS	-0.925	-0.897	-0.911	1.007	0.815	0.911
SLIT2	-0.887	-0.887	-0.887	0.517	1.258	0.887
HSPG2	-0.814	-1.002	-0.908	0.771	1.045	0.908
SERPINB4	0.920	0.906	0.913	-0.938	-0.888	-0.913
CALD1	-0.885	-0.939	-0.912	0.980	0.844	0.912
CLIC1	-0.892	-0.933	-0.913	0.896	0.930	0.913
RPL13A	-0.881	-0.939	-0.910	0.792	1.029	0.910
UQCRC2	0.991	0.821	0.906	-0.730	-1.082	-0.906
LAMB1	-0.973	-0.851	-0.912	0.911	0.913	0.912
RPL7	1.111	0.698	0.904	-0.974	-0.835	-0.904
RAB21	0.903	0.922	0.913	-0.903	-0.922	-0.913
FBLN1	1.110	0.686	0.898	-0.706	-1.090	-0.898
RTN4	-0.938	-0.886	-0.912	0.953	0.872	0.912
PTMS	0.951	0.874	0.913	-0.902	-0.924	-0.913
NOP56	-0.917	-0.904	-0.911	1.017	0.805	0.911
TAGLN2	-0.637	-1.148	-0.893	0.683	1.102	0.893
RPS18	-0.908	-0.908	-0.908	0.748	1.068	0.908
ANXA1	-0.868	-0.953	-0.910	1.020	0.801	0.910
AFP	1.643	-0.137	0.753	-0.827	-0.679	-0.753
RAP1B	0.878	0.947	0.913	-0.939	-0.886	-0.913
HSP90AB1	-0.968	-0.856	-0.912	0.889	0.936	0.912
PHGDH	-0.914	-0.908	-0.911	1.022	0.799	0.911
ALCAM	-0.907	-0.902	-0.904	1.120	0.688	0.904
RPL17	0.951	0.874	0.913	-0.905	-0.920	-0.913
COL1A2	-0.912	-0.912	-0.912	0.847	0.977	0.912
TMX1	1.016	0.806	0.911	-0.885	-0.937	-0.911
GBE1	1.075	0.741	0.908	-0.908	-0.908	-0.908
SLC16A3	0.980	0.844	0.912	-0.926	-0.898	-0.912
KRT1	-0.902	-0.920	-0.911	1.005	0.818	0.911
PLS3	0.993	0.829	0.911	-0.853	-0.969	-0.911
H3-3A	-0.923	-0.900	-0.911	0.820	1.003	0.911
PARP1	-1.083	-0.728	-0.906	0.993	0.819	0.906
CLEC3B	-0.910	-0.915	-0.913	0.949	0.876	0.913
CAV1	-0.903	-0.895	-0.899	1.174	0.624	0.899
S100A13	-0.903	-0.903	-0.903	0.674	1.133	0.903
CPPED1	1.052	0.755	0.903	-1.077	-0.730	-0.903
IARS1	1.025	0.796	0.910	-0.941	-0.880	-0.910
RDX	-0.916	-0.910	-0.913	0.908	0.918	0.913
DPP4	-0.912	-0.912	-0.912	0.969	0.856	0.912
IGHA1	-0.898	-0.927	-0.912	0.957	0.868	0.912
GJA1	0.943	0.882	0.913	-0.924	-0.902	-0.913
CAP1	-0.909	-0.909	-0.909	1.057	0.761	0.909
MANF	-0.908	-0.915	-0.912	0.990	0.834	0.912

RPLP0	-0.902	-0.922	-0.912	0.847	0.977	0.912
PARVA	0.911	0.915	0.913	-0.913	-0.913	-0.913
RPL36AL	0.619	1.178	0.898	-0.887	-0.909	-0.898
CAPN5	-0.911	-0.914	-0.913	0.949	0.876	0.913
H2BC21	-1.051	-0.758	-0.905	0.749	1.060	0.905
CKM	0.758	1.059	0.909	-0.892	-0.925	-0.909
RAB19	1.129	0.676	0.903	-0.832	-0.973	-0.903
RO60	-0.853	-0.938	-0.896	0.592	1.199	0.896
GLA	-0.918	-0.908	-0.913	0.934	0.892	0.913
ITGA6	-0.906	-0.919	-0.913	0.952	0.873	0.913
RRAS	0.761	1.056	0.908	-0.857	-0.959	-0.908
CNPY2	0.358	1.371	0.865	-0.879	-0.851	-0.865
NPM1	-0.955	-0.857	-0.906	1.089	0.724	0.906
ACO1	-0.854	-0.970	-0.912	0.949	0.875	0.912
ENG	-0.903	-0.919	-0.911	1.013	0.809	0.911
GNAI2	-0.770	-1.045	-0.908	0.807	1.008	0.908
PVR	-0.906	-0.918	-0.912	0.972	0.852	0.912
ANXA2	-0.824	-0.991	-0.908	1.051	0.764	0.908
COL1A1	-0.881	-0.935	-0.908	1.064	0.753	0.908
DDOST	0.816	1.006	0.911	-0.911	-0.911	-0.911
PKM	-0.891	-0.929	-0.910	0.786	1.034	0.910
GSTO1	-0.880	-0.945	-0.913	0.919	0.906	0.913
Runx2	-0.915	-0.911	-0.913	0.906	0.919	0.913
DDX5	-0.890	-0.917	-0.904	0.678	1.129	0.904
EDIL3	-0.903	-0.922	-0.912	0.852	0.972	0.912
CPN1	-0.915	-0.910	-0.913	0.948	0.877	0.913
C1QBP	1.186	0.595	0.891	-1.071	-0.710	-0.891
GLIPR2	0.687	1.121	0.904	-0.868	-0.940	-0.904
CCT7	0.965	0.860	0.912	-0.895	-0.930	-0.912
NCL	-0.943	-0.874	-0.909	0.758	1.059	0.909
CD59	-0.891	-0.935	-0.913	0.899	0.927	0.913
CTSZ	-0.918	-0.908	-0.913	0.915	0.910	0.913
RCN3	0.868	0.957	0.912	-0.890	-0.935	-0.912
GRIA3	0.988	0.835	0.912	-0.912	-0.912	-0.912
LAMTOR1	1.027	0.793	0.910	-0.875	-0.945	-0.910
BHMT	-0.934	-0.891	-0.913	0.913	0.912	0.913
RAB8A	-0.913	-0.913	-0.913	0.951	0.874	0.913
FOXO1	-0.879	-0.946	-0.913	0.922	0.903	0.913
FKBP1A	0.823	1.000	0.911	-0.911	-0.911	-0.911
HEYL	1.127	0.680	0.904	-0.897	-0.910	-0.904
NIBAN2	0.904	0.922	0.913	-0.921	-0.905	-0.913
LTF	1.068	0.748	0.908	-0.925	-0.891	-0.908
RAB11A	-0.903	-0.921	-0.912	0.969	0.856	0.912
SRSF10	-0.922	-0.898	-0.910	1.032	0.789	0.910

GNAQ	-0.880	-0.946	-0.913	0.901	0.924	0.913
P4HB	-0.915	-0.910	-0.913	0.923	0.902	0.913
PCDHGB6	-0.905	-0.916	-0.911	0.801	1.020	0.911
RNH1	-0.891	-0.934	-0.913	0.939	0.887	0.913
CD151	1.064	0.753	0.908	-0.893	-0.924	-0.908
RPL15	0.973	0.852	0.912	-0.921	-0.903	-0.912
RHOA	-0.913	-0.913	-0.913	0.895	0.930	0.913
PPIB	-0.905	-0.918	-0.912	0.992	0.831	0.912
ITGA3	0.956	0.869	0.913	-0.917	-0.908	-0.913
LDHA	-0.906	-0.913	-0.910	0.776	1.043	0.910
GPX3	1.083	0.731	0.907	-0.874	-0.940	-0.907
YWHAB	0.859	0.936	0.898	-1.183	-0.612	-0.898
MYOF	1.123	0.684	0.903	-0.844	-0.962	-0.903
RPS9	-0.884	-0.896	-0.890	0.535	1.244	0.890
KPRP	-0.907	-0.918	-0.913	0.909	0.916	0.913
SH3BGRL3	-0.913	-0.913	-0.913	0.868	0.957	0.913
DSTN	0.829	0.994	0.912	-0.913	-0.910	-0.912
SF3B2	0.512	1.232	0.872	-0.573	-1.171	-0.872
MEP1A	0.703	1.107	0.905	-0.872	-0.938	-0.905
ERO1A	0.928	0.898	0.913	-0.895	-0.931	-0.913
ACTN4	-0.912	-0.912	-0.912	0.969	0.855	0.912
MFGE8	-0.910	-0.913	-0.912	0.828	0.996	0.912
EML2	-0.914	-0.908	-0.911	1.014	0.807	0.911
IMPDH2	1.036	0.784	0.910	-0.910	-0.909	-0.910
CALHM5	0.922	0.904	0.913	-0.913	-0.913	-0.913
PTGES3	-0.897	-0.929	-0.913	0.907	0.919	0.913
EIF2S2	-0.898	-0.928	-0.913	0.914	0.911	0.913
BZW1	1.012	0.810	0.911	-0.902	-0.920	-0.911
DDAH2	-0.888	-0.937	-0.913	0.927	0.898	0.913
YWHAE	0.998	0.823	0.910	-0.834	-0.987	-0.910
GDF5	-0.945	-0.880	-0.912	0.966	0.928	0.912
CPA4	1.027	0.794	0.910	-0.921	-0.899	-0.910
RPL27	0.902	0.921	0.912	-0.829	-0.994	-0.912
RSL1D1	-0.920	-0.906	-0.913	0.949	0.877	0.913
RPL8	-0.884	-0.939	-0.912	0.840	0.983	0.912
KRT10	-0.910	-0.910	-0.910	1.032	0.788	0.910
VIM	-0.842	-0.982	-0.912	0.921	0.903	0.912
ITGB3	-0.865	-0.950	-0.907	0.738	1.077	0.907
PDCD6IP	1.030	0.790	0.910	-0.875	-0.945	-0.910
GSN	0.950	0.876	0.913	-0.894	-0.931	-0.913
C3	-0.907	-0.918	-0.912	0.863	0.962	0.912
ANXA7	1.116	0.694	0.905	-0.901	-0.909	-0.905
ALDOC	0.814	0.981	0.898	-1.171	-0.624	-0.898
RPL18A	-0.845	-0.977	-0.911	0.842	0.980	0.911

SNRPD3	-0.910	-0.915	-0.912	0.971	0.854	0.912
GNA13	1.202	0.585	0.893	-0.997	-0.790	-0.893
S100A6	-0.904	-0.921	-0.912	0.967	0.858	0.912
PDIA4	-0.912	-0.913	-0.913	0.938	0.887	0.913
OCT4	-0.944	-0.878	-0.911	1.010	0.812	0.911
SLC9A3R1	-0.850	-0.867	-0.858	0.318	1.398	0.858
ITGB1	0.882	0.944	0.913	-0.908	-0.917	-0.913
ATP2B4	0.913	0.912	0.913	-0.899	-0.927	-0.913
NACA	1.052	0.764	0.908	-0.832	-0.984	-0.908
LGALSL	0.835	0.983	0.909	-0.791	-1.027	-0.909
SRC	1.020	0.794	0.907	-0.767	-1.047	-0.907
RBM25	-0.877	-0.948	-0.912	0.876	0.948	0.912
RPS23	1.078	0.737	0.907	-0.883	-0.932	-0.907
B2M	-0.863	-0.936	-0.900	1.166	0.633	0.900
FKBP2	-0.804	-1.016	-0.910	0.964	0.857	0.910
NAP1L1	1.492	0.161	0.826	-0.738	-0.915	-0.826
PRKDC	1.106	0.704	0.905	-0.887	-0.924	-0.905
ACTBL2	-0.913	-0.913	-0.913	0.912	0.914	0.913
COL6A3	1.088	0.725	0.907	-0.918	-0.896	-0.907
VTN	0.764	1.054	0.909	-0.895	-0.923	-0.909
SERPINC1	0.947	0.878	0.913	-0.939	-0.886	-0.913
NME1	1.231	0.540	0.886	-0.723	-1.049	-0.886
APRT	1.008	0.814	0.911	-0.926	-0.896	-0.911
BASP1	-0.912	-0.912	-0.912	0.992	0.831	0.912
ZYX	-0.892	-0.919	-0.906	1.105	0.706	0.906
TXNDC5	0.188	1.478	0.833	-0.898	-0.767	-0.833
APOC3	1.344	0.398	0.871	-0.869	-0.873	-0.871
HNRNPDL	-0.922	-0.900	-0.911	1.013	0.809	0.911
COL6A1	0.935	0.890	0.912	-0.958	-0.867	-0.912
KRT2	0.867	0.957	0.912	-0.944	-0.881	-0.912
SUPT16H	0.998	0.818	0.908	-0.773	-1.043	-0.908
TAGLN	-0.905	-0.883	-0.894	1.216	0.571	0.894
CALU	-0.812	-0.974	-0.893	1.210	0.576	0.893
PSMA3	-0.913	-0.913	-0.913	0.884	0.942	0.913
HSP90AA1	0.864	0.961	0.912	-0.903	-0.922	-0.912
U2AF2	-0.911	-0.911	-0.911	1.024	0.798	0.911
GSTM2	-0.959	-0.860	-0.910	1.029	0.791	0.910
KIDINS220	-0.893	-0.925	-0.909	0.767	1.051	0.909
FLNC	-0.911	-0.911	-0.911	1.009	0.814	0.911
H2BC18	-0.929	-0.896	-0.913	0.890	0.936	0.913
KRT16	-0.922	-0.904	-0.913	0.893	0.933	0.913
SLC4A1	-0.890	-0.925	-0.908	0.738	1.077	0.908
SRSF6	0.737	1.078	0.907	-0.941	-0.874	-0.907
PSMB5	1.216	0.570	0.893	-0.820	-0.965	-0.893

VCL	-0.955	-0.870	-0.913	0.921	0.904	0.913
SERPINF2	-0.897	-0.923	-0.910	0.781	1.039	0.910
PHB2	0.970	0.855	0.912	-0.904	-0.921	-0.912
YAP1	1.003	0.819	0.911	-0.911	-0.911	-0.911
HNRNPU	-0.913	-0.913	-0.913	0.906	0.920	0.913
YWHAQ	0.359	1.371	0.865	-0.865	-0.865	-0.865
CALR	-0.858	-0.965	-0.911	0.842	0.981	0.911
CTNNB1	-0.772	-0.843	-0.807	0.071	1.544	0.807
LAMC1	0.596	1.197	0.896	-0.920	-0.872	-0.896
SERPINB12	0.880	0.933	0.907	-0.727	-1.087	-0.907
GPI	-0.902	-0.923	-0.912	0.964	0.860	0.912
RNF11	0.807	1.014	0.911	-0.893	-0.929	-0.911
LTA4H	-0.887	-0.906	-0.897	1.194	0.599	0.897
TECPR2	-0.920	-0.901	-0.911	0.801	1.020	0.911
PGAM1	-0.902	-0.923	-0.913	0.933	0.893	0.913
ITGA5	-0.912	-0.913	-0.913	0.949	0.876	0.913
DCD	-0.957	-0.868	-0.912	0.895	0.930	0.912
FSTL1	0.997	0.826	0.912	-0.912	-0.911	-0.912
APOB	-0.850	-0.974	-0.912	0.855	0.968	0.912
MYL12B	-0.942	-0.883	-0.913	0.910	0.916	0.913
AP3D1	0.788	1.032	0.910	-0.930	-0.890	-0.910
RPS15A	0.795	1.026	0.910	-0.902	-0.919	-0.910
F10	1.168	0.629	0.899	-0.828	-0.969	-0.899
H4C1	-0.977	-0.844	-0.911	1.002	0.819	0.911
RPS3A	-0.914	-0.911	-0.913	0.896	0.929	0.913
RHOG	0.986	0.835	0.911	-0.995	-0.826	-0.911
COL3A1	1.203	0.584	0.893	-0.990	-0.797	-0.893
FN1	-0.946	-0.878	-0.912	0.976	0.848	0.912
MYL6	-0.907	-0.918	-0.913	0.899	0.926	0.913
VCP	-0.928	-0.897	-0.912	0.962	0.863	0.912
ARPC5	-0.896	-0.896	-0.896	1.201	0.590	0.896
CDC42	0.882	0.942	0.912	-0.851	-0.973	-0.912
G6PD	0.902	0.924	0.913	-0.943	-0.882	-0.913
CTNNA1	0.934	0.888	0.911	-0.810	-1.012	-0.911
P2RX1	-0.913	-0.913	-0.913	0.912	0.913	0.913
COTL1	-0.890	-0.933	-0.912	0.982	0.841	0.912
ABI1	-0.916	-0.861	-0.889	0.528	1.249	0.889
DSG2	-0.750	-1.065	-0.907	0.840	0.975	0.907
PSMB6	0.967	0.858	0.912	-0.910	-0.915	-0.912
FSCN1	-0.906	-0.913	-0.909	0.769	1.050	0.909
HSPA5	-0.910	-0.910	-0.910	1.041	0.779	0.910
CTSB	0.892	0.934	0.913	-0.905	-0.921	-0.913
UCHL1	-0.904	-0.919	-0.911	1.008	0.815	0.911
SPP2	-0.910	-0.916	-0.913	0.925	0.901	0.913

PRDX6	1.081	0.733	0.907	-0.885	-0.930	-0.907
ARPC2	-0.901	-0.913	-0.907	0.730	1.084	0.907
CPNE2	1.020	0.789	0.904	-1.086	-0.723	-0.904
ACTN1	-0.817	-1.004	-0.911	0.958	0.864	0.911
ALDOA	-0.912	-0.912	-0.912	0.834	0.990	0.912
MYL9	0.196	1.474	0.835	-0.853	-0.817	-0.835
